# Prevalence of and risk factors for microscopic and submicroscopic malaria infections in pregnancy: a systematic review and meta-analysis

**DOI:** 10.1016/S2214-109X(23)00194-8

**Published:** 2023-06-02

**Authors:** Anna Maria van Eijk, Kasia Stepniewska, Jenny Hill, Steve M Taylor, Stephen J Rogerson, Gilles Cottrell, R Matthew Chico, Julie R Gutman, Halidou Tinto, Holger W Unger, Stephanie K Yanow, Steven R Meshnick, Feiko O ter Kuile, Alfredo Mayor, the Subpatent Malaria in Pregnancy Group

**Affiliations:** Department of Clinical Sciences, Liverpool School of Tropical Medicine, Liverpool, UK (A M van Eijk PhD, J Hill PhD, Prof F O ter Kuile PhD, H W Unger PhD); Centre for Tropical Medicine and Global Health, Nuffield Department of Clinical Medicine, University of Oxford, Oxford, UK (K Stepniewska PhD); Division of Infectious Diseases and Duke Global Health Institute, Duke University, Durham, NC, USA (S M Taylor MD); Department of Infectious Diseases, Doherty Institute, The University of Melbourne, Melbourne, VIC, Australia (Prof S J Rogerson PhD); Université Paris-Cité, IRD, MERIT, F-75006 Paris, France (G Cottrell PhD); Department of Disease Control, London School of Hygiene & Tropical Medicine, London, UK (R M Chico PhD); Malaria Branch, Division of Parasitic Diseases and Malaria, Center for Global Health, Centers for Disease Control and Prevention, Atlanta, GA, USA (J R Gutman MD); Institut de Recherche en Sciences de la Sant-Unité de Recherche Clinique de Nanoro, Ouagadougou, Burkina Faso (Prof H Tinto PhD); Menzies School of Health Research, Charles Darwin University, Darwin, NT, Australia (H W Unger); School of Public Health, Department of Medical Microbiology and Immunology, University of Alberta, Edmonton, AB, Canada (Prof S K Yanow PhD); ISGlobal, Barcelona Institute for Global Health, Hospital Clínic-Universitat de Barcelona, Barcelona, Spain (Prof A Mayor PhD); Centro de Investigação em Saúde de Manhiça, Maputo, Mozambique (Prof A Mayor); Institute for Global Health and Infectious Diseases, School of Medicine, University of North Carolina, Chapel Hill, NC, USA (Prof S R Meshnick†)

## Abstract

**Background:**

Malaria infections during pregnancy can cause adverse birth outcomes, yet many infections are undetected by microscopy. We aimed to describe the epidemiology of submicroscopic malaria infections in pregnant women in Asia, the Americas, and Africa using aggregated and individual participant data (IPD).

**Methods:**

For this systematic review and meta-analysis, studies (published Jan 1, 1997 to Nov 10, 2021) with information on both microscopic and submicroscopic infections during pregnancy from Asia, the Americas, or Africa, identified in the Malaria-in-Pregnancy Library, were eligible. Studies (or subgroups or study groups) that selected participants on the basis of the presence of fever or a positive blood smear were excluded to avoid selection bias. We obtained IPD (when available) and aggregated data. Estimates of malaria transmission intensity and sulfadoxine–pyrimethamine resistance, matched by study location and year, were obtained using publicly available data. One-stage multivariable logit and multinomial models with random intercepts for study site were used in meta-analysis to assess prevalence of and risk factors for submicroscopic infections during pregnancy and at delivery. This study is registered with PROSPERO, number CRD42015027342.

**Findings:**

The search identified 87 eligible studies, 68 (78%) of which contributed to the analyses. Of these 68 studies, 45 (66%) studies contributed IPD (48 869 participants) and 23 (34%) studies contributed aggregated data (11 863 participants). During pregnancy, median prevalence estimates were 13·5% (range 0·0–55·9, 66 substudies) for submicroscopic and 8·0% (0·0–50·6, 66 substudies) for microscopic malaria. Among women with positive *Plasmodium* nucleic acid amplification tests (NAATs), the median proportion of submicroscopic infections was 58·7% (range 0·0–100); this proportion was highest in the Americas (73·3%, 0·0–100), followed by Asia (67·2%, 36·4–100) and Africa (56·5%, 20·5–97·7). In individual patient data analysis, compared with women with no malaria infections, those with submicroscopic infections were more likely to present with fever in Africa (adjusted odds ratio 1·32, 95% CI 1·02–1·72; p=0·038) but not in other regions. Among women with NAAT-positive infections in Asia and the Americas, *Plasmodium vivax* infections were more likely to be submicroscopic than *Plasmodium falciparum* infections (3·69, 2·45–5·54; p<0·0001). Risk factors for submicroscopic infections among women with NAAT-positive infections in Africa included older age (age ≥30 years), multigravidity, and no HIV infection.

**Interpretation:**

During pregnancy, submicroscopic infections are more common than microscopic infections and are associated with fever in Africa. Malaria control in pregnancy should target both microscopic and submicroscopic infections.

**Funding:**

Bill & Melinda Gates Foundation through the Worldwide Antimalarial Resistance Network.

## Introduction

Malaria infection during pregnancy causes adverse maternal and pregnancy outcomes.^1^ In 2020, approximately 122 million pregnancies occurred in areas of malaria transmission.^2^ *Plasmodium falciparum* is the most common and pernicious malaria species; the effects of *Plasmodium vivax* on pregnancy outcomes in Asia and the Americas are considerable, although generally less severe than *P falciparum*.^3–6^

The increased use of sensitive nucleic acid amplification tests (NAATs) such as PCR and loop-mediated isothermal amplification (LAMP) has shown that many malaria infections in pregnancy remain asymptomatic and below the limit of detection by microscopy. Malaria microscopy typically detects infections with at least 50–500 parasites per μL. PCR can detect fewer than five parasites per μL and has the added advantage of more accurate species identification than microscopy.^7–9^ However, PCR can also detect gametocytes or non-viable asexual parasites after malaria therapy, neither of which cause clinical harm.^10^ The diagnosis of malaria in pregnancy is further challenged by the accumulation of parasites in the placenta, with low or undetectable parasite densities in peripheral blood.^11^

Insights into the epidemiology of submicroscopic infections in pregnancy can guide the design of strategies to detect, manage, and prevent malaria in pregnancy, including in the first trimester.^12^ Furthermore, understanding the contribution of submicroscopic infections in pregnancy to the infectious reservoir is important for malaria elimination efforts,^13^ such as during mass drug administration campaigns, which often exclude pregnant women because of concerns about drug safety for the developing fetus. Furthermore, understanding patterns of submicroscopic infections supports the interpretation of parasite prevalence data collected at antenatal clinics for surveillance.^14^

We conducted a systematic review to describe the epidemiology of submicroscopic and microscopic infections during pregnancy and at delivery. We aimed to assess the relationship between malaria infections, species, and fever, and any association with age, gravidity, geographical region, and intensity of malaria transmission. Furthermore, we aimed to investigate the progression of submicroscopic malaria.

## Methods

### Search strategy and selection criteria

In this systematic review and meta-analysis, we used aggregated and individual participant data (IPD). The Malaria in Pregnancy Library was the main search engine used. The Malaria in Pregnancy Library combines data from over 20 sources including Medline, Web of Knowledge, Scopus, Cumulative Index to Nursing and Allied Health Literature, Bioline, the Cochrane Library databases, the WHO Global Health Library, and grey literature (ie, reports, unpublished studies, and theses) and is updated every 4 months.^15^ We searched for records published from Jan 1, 1997 (a year before the first mention of PCR in the Malaria in Pregnancy Library) to Nov 10, 2021, using the search terms “polymerase chain reaction” OR “PCR” OR “submicroscopic” OR “sub-microscopic” OR “sub-patent” OR “subpatent” OR “Lamp” OR “loop-mediated isothermal amplification” without language restrictions (appendix p 6). The search was repeated in Pubmed, Google Scholar, and the Global Health database with “AND pregnan* AND malaria” added to the search terms to ensure completeness. Studies were eligible if they contained data from both a NAAT-based diagnostic (either PCR or LAMP) and microscopy from the same women during pregnancy or at delivery. Studies (or subgroups or study groups) that selected participants on the basis of the presence of fever or a positive blood smear were excluded to avoid selection bias. Two authors (AmvE and AM) reviewed the potential studies. Any discrepancies were resolved after further discussion until consensus was reached. The protocol is available through the WorldWide Antimalarial Resistance Network.

### Data analysis

AmvE, AM, JH, and FotK contacted investigators of source studies to request primary data. Prespecified variables (appendix p 7) were obtained and standardised. For studies for which IPD were unavailable, data about number of infections and number of women examined were extracted from the published articles as aggregated data when there was sufficient information. Estimates of malaria transmission intensity and sulfadoxine–pyrimethamine resistance, matched by study location and year, were extracted from publicly available data (appendix p 8).^16–18^ The risk of bias in primary studies was assessed using a modified version of the Newcastle-Ottawa quality assessment tool for observational studies (appendix pp 7–8). Two authors (AmvE and AM) independently assessed the risk of bias across six domains. Disagreements were resolved by discussion until consensus was reached.

Submicroscopic malaria was defined as microscopy-negative infections detected by NAAT-based methods.^19^ Infections that were microscopy-positive but NAAT-negative were excluded from the analyses. Fever was defined as documented fever (≥37·5°C) or a history of fever in the previous 7 days as per the definition used in the source studies. Reported antimalarial use and intermittent preventive treatment in pregnancy (IPTp) were combined into one variable, indicating a history of antimalarial use in pregnancy. PCR results were used for species identification. We presented results by region (Americas [central and South America], Asia [Asia and the Pacific], and Africa) because of differences in species distribution, transmission intensity, prevention strategies, and sulfadoxine–pyrimethamine resistance patterns. Because of the observed interactions between moderate-to-low and high transmission areas for gravidity, results in Africa were presented stratified by high (*P falciparum* prevalence for children aged 2–10 years [*Pf*PR_2–10_] of at least 35%) and moderate-to-low transmission areas (*Pf*PR_2–10_ lower than 35%). Potential predictors and confounders included age, gravidity, season, year of study, gestational age, bednet or insecticide-treated net (ITN) use, HIV status, residence (rural or urban), antimalarial use for treatment or prevention, and study design (survey, cohort study or trial).

The risk of microscopic and submicroscopic malaria was evaluated in peripheral maternal blood during pregnancy and delivery and in placental blood. Studies were split by location when possible. Weighted pooled prevalence estimates for submicroscopic and microscopic malaria and weighted pooled odds ratios (ORs) were obtained using random-effects two-stage models using IPD and aggregated data (metaprop and meta, Stata 17). Because of the high observed heterogeneity between studies (which we assessed with the *I*^2^ statistic), prevalence results were also summarised as study median and range. Ranges were used instead of IQRs because a range provides a fast overview of all the possible values, whereas an IQR does not provide this information.

One-stage IPD analyses were conducted for presence of fever at enrolment with submicroscopic and microscopic malaria as exposures (outcome 1); submicroscopic infection at enrolment with malaria species as the exposure among women with infection (outcome 2); malaria infection status (microscopic, submicroscopic, or none) in pregnancy and at delivery and associated risk factors (outcome 3); and infection status (microscopic, submicroscopic, or none) at the second scheduled study visit for women who had submicroscopic malaria at enrolment (outcome 4). Logistic regression models (xtlogit, Stata version 17) for outcomes 1 and 2 and multinomial logistic regression models (gsem, Stata version 17) for outcomes 3 and 4 were fitted with a random intercept for study site.

For outcome 3 (risk factors for malaria infection), a base model was first developed by region (Africa [high or moderate-to-low transmission], Asia, or the Americas) that included covariates with at least 95% overall availability: age, gravidity, malaria season, an indicator of malaria transmission by study year, an indicator for first antenatal clinic visit (yes *vs* no or unknown; during pregnancy only), and study design (survey *vs* trial or cohort; at delivery only). This base model was then used to assess the effect of variables when a considerable proportion (generally >5%) was missing (not collected or reported): gestational age, HIV status, rural versus urban residence, ITN and bednet use, and history of antimalarial use. For outcome 1 (fever), models were adjusted for base model covariates and gestational age. For outcome 2 (species as a risk factor for submicroscopic malaria), submicroscopic infection among NAAT-positive infections was the outcome of interest with microscopic malaria as the reference group and species as the exposure variable of interest, with *P falciparum* as the reference category. For outcome 4 (infection status at the second scheduled study visit for women who had submicroscopic malaria at enrolment), the covariates explored were base model variables, the interval between study visits in days, markers of sulfadoxine–pyrimethamine resistance, and antimalarial treatment at enrolment. High sulfadoxine–pyrimethamine resistance was defined as a prevalence of Lys540Glu of at least 30% in east and southern Africa and Ala437Gly of at least 90% in central and west Africa, whereas low resistance was defined as Lys540Glu of less than 30% in east and southern Africa, and Ala437Gly of less than 90% in central and west Africa. Factors with a p value of at least 0·1 (Wald test) in the multivariate model were removed. To explore the robustness of findings, we used alternative measures of transmission intensity based on malaria infection prevalence by PCR assessed in the first trimester among all gravidae and established whether the data source (IPD *vs* aggregated) affected prevalence or whether study quality or quality of blood slide reading affected the risk factor analysis. All analyses were conducted using Stata (version 17). Further methodological details are described in the appendix (p 8). The study is registered with PROSPERO, CRD42015027342.

### Role of the funding source

The funder had no role in the study design, data collection, analysis, interpretation, or writing of the report.

## Results

**Figure 1:**
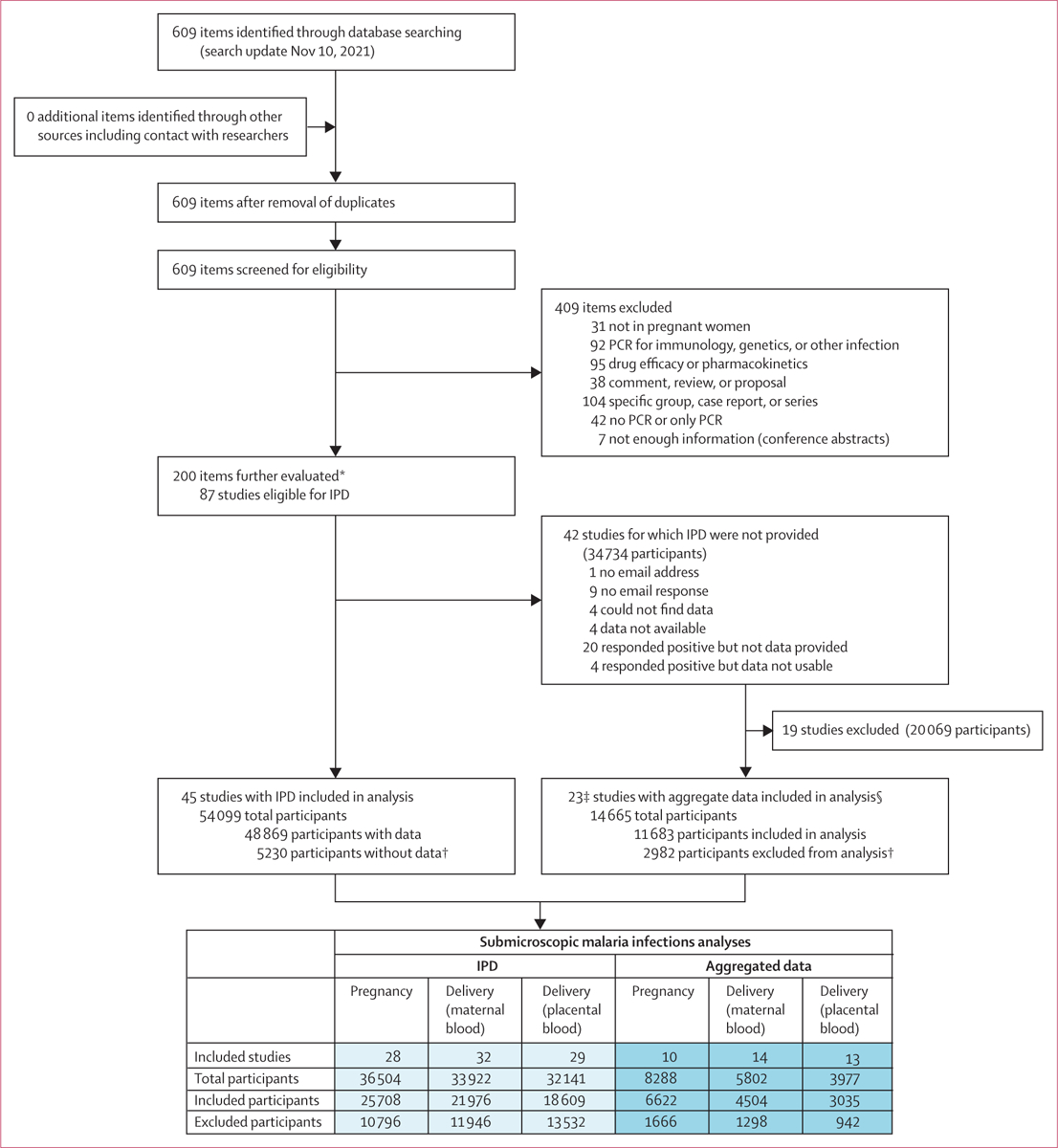
PRISMA flow diagram showing study selection process IPD=individual participant data. *Items refer to articles, abstracts, reports, or theses. Items could publish from the same study. From the 200 items, 87 studies were identified that were eligible for IPD. †Reasons for non-availability of submicroscopic infections at the individual scale (excluded participants): nucleic acid amplification tests not conducted or not available or blood smear not available; submicroscopic information not available at all available timepoints (cohort studies); or only submicroscopic information for a random sample of participants but not for the full set. ‡One study had two publications which covered different time periods from the same study. §Studies from which data could be extracted.

The search identified 87 eligible studies, 68 (78%) of which contributed to the analyses. Of these 68 studies, 45 (66%) studies had available IPD (48 869 participants) and 23 (34%) had aggregated data (11 683 participants; figure 1). The included studies were conducted between 1995 and 2017 in 27 countries and included 15 trials, 39 surveys (ie, single observations), and 14 cohort studies (appendix pp 15–21). 54 (79%) studies (35 with IPD) were from Africa, eight (12%) studies (six with IPD) were from Asia, and seven (10%) studies (five with IPD) were from the Americas. One (1%) study was conducted in both Asia and the Americas. The 68 studies provided 83 datapoints by location (appendix p 58). Over half of the included participants (54–63%, depending on test and outcome) were enrolled in clinical trials. The risk of bias was scored as moderate to low in 46 (68%) studies and high in 22 (32%) studies (appendix pp 22–24). Four (6%) studies used LAMP, and the remaining 64 (94%) studies used PCR. Of the 64 studies that used PCR, 30 (47%) studies used quantitative real-time PCR, and 31 (48%) studies used nested PCR. The remaining three studies used both methods, a PCR-based ligase detection reaction–fluorescent microsphere assay, or did not have details on molecular method (appendix p 12). In studies that used PCR, the 18S ribosomal RNA gene was most commonly targeted (47 [73%] of 64 studies; appendix p 12). Most studies (44 [65%] of 68) reported malaria infection data from maternal peripheral blood at delivery and were conducted in moderate and high transmission areas (51 [75%] of 68; appendix p 25). The availability of data on age and gravidity ranged from 87% to more than 99% of participants, depending on the outcome, whereas the availability of other covariables ranged from 4% to 96% (appendix p 26). Data on antimalarial use was available for 8371 (33%) of 25 401 women during pregnancy and for 20 295 (93%) of 21 820 participants at delivery with maternal blood testing and 17 693 (96%) of 18 451 participants with placental blood testing. Antimalarials were reported to be used by 1054 (13%) of 8371 women during pregnancy, 15 794 (78%) of 20 295 participants with maternal blood testing at delivery, and 13 680 (77%) of 17 693 participants with placental blood testing (42% and 48% received ≥2 courses at delivery, respectively). Malaria test results and the proportion of participants with microscopy positive and NAAT negative results are shown in the appendix (pp 27–30).

**Figure 2:**
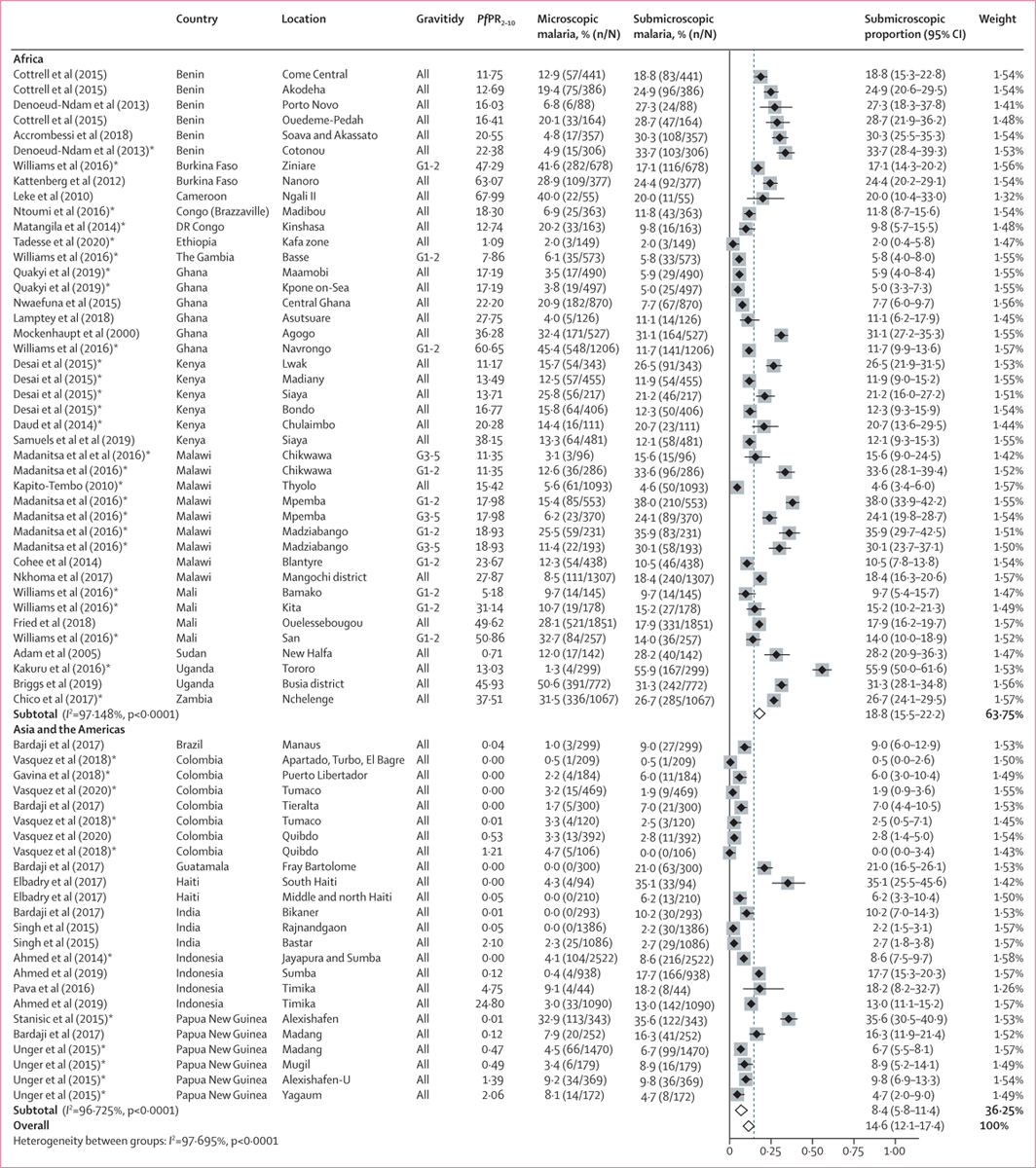
Prevalence of submicroscopic malaria in pregnancy, by study, 1998–2019 The pooled estimate was obtained using metaprop combining individual patient data and aggregated data, using information during pregnancy. Studies in Asia and the Pacific were included under Asia and studies in central or South America were included under the Americas. G1=primigravidae. G2=secundigravidae. G3+=multigravidae. *Pf*PR_2–10_=*Plasmodium falciparum* prevalence among children aged 2–10 years at the year of study visit, as estimated by the Malaria Atlas Project. *Enrolment criteria could have affected parasite prevalence (appendix p 28).

The pooled prevalence estimates for peripheral blood in pregnancy (at enrolment for cohorts or trials or during surveys in pregnancy) were 14·6% (95% CI 12·1–17·4) for submicroscopic malaria (figure 2) and 9·7% (6·9–13·0) for microscopic malaria. Median prevalence estimates during pregnancy were similar: 13·5% (range 0·0–55·9) for submicroscopic and 8·0% (0·0–50·6) for microscopic malaria (table 1). At delivery, when maternal peripheral blood was tested, pooled prevalence estimates were 10·1% (95% CI 8·0–12·4) for submicroscopic and 4·0% (2·9–5·4) for microscopic infection. The prevalence of submicroscopic and microscopic malaria was highest in Africa within high transmission areas (table 1).

**Figure 3:**
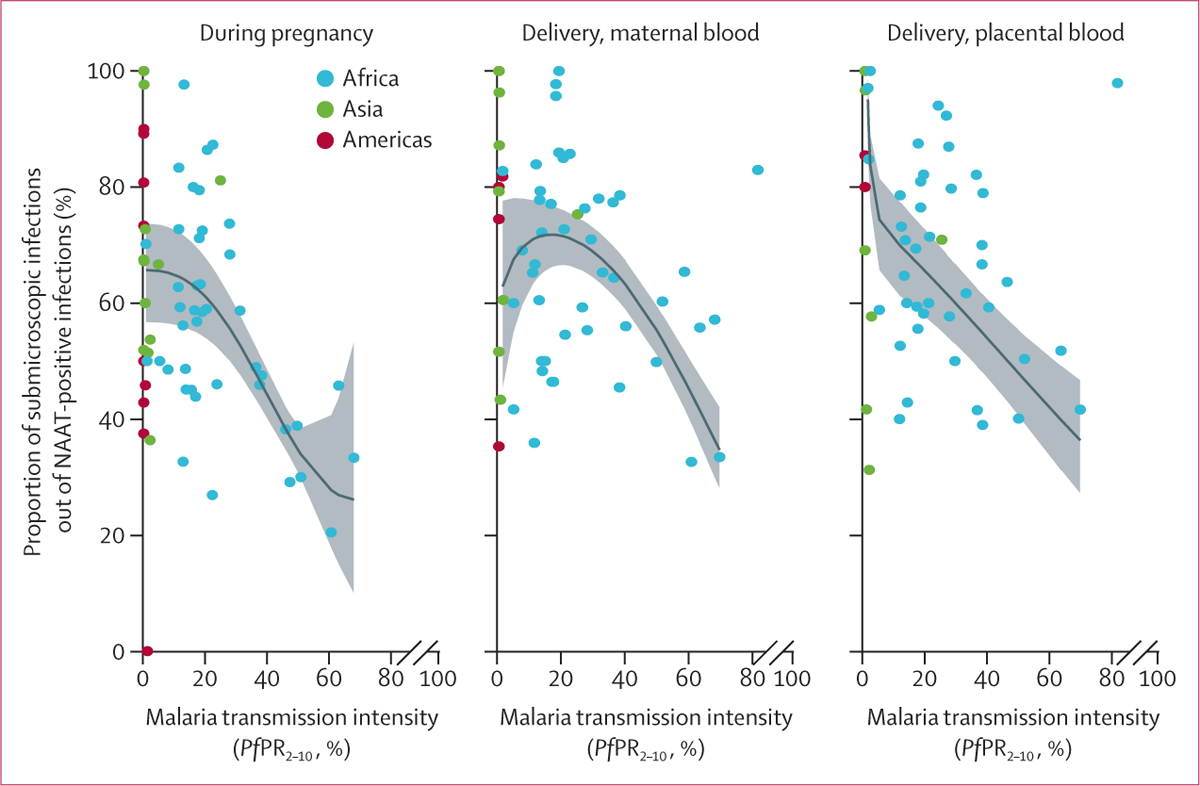
Proportion of submicroscopic malaria among women with NAAT-positive test results by malaria transmission rate Studies in Asia and the Pacific were combined under Asia; studies in central or South America were combined under the Americas. The relationship and 95% CI were estimated for Africa only using fractional polynomials logistic regression with robust variance (p<0·0001 for comparison with linear and non-covariate model); for modelling the graphs at delivery, one outlier at *Pf*PR_2–10_ of 81% was omitted (for the plot for first antenatal clinic visit and by gravidity, see appendix pp 59–60). NAAT=nucleic acid amplification test (PCR or loop-mediated isothermal amplification). *Pf*PR_2–10_=*Plasmodium falciparum* prevalence among children aged 2–10 years at the year of study visit, as estimated by the Malaria Atlas Project.

Among participants with NAAT-positive infections, the pooled proportion of submicroscopic infections was 62·5% (95% CI 56·5–68·3) during pregnancy, and at delivery, it was 72·3% (67·1–77·2) in peripheral blood and 72·7% (66·8–78·3) in placental blood (table 1). The proportion of submicroscopic infections at delivery among women who had NAAT-positive infections was highest in the Americas. In Africa, the proportion of women with submicroscopic infection was lower in areas of high transmission than in areas of low transmission during pregnancy, but not at delivery (figure 3; appendix pp 59–60). During pregnancy, 413 (9%) of 4856 participants with a NAAT-positive infection had a documented fever or a history of fever in the previous 7 days, with a median of 1·2% (range 0·0–36·4) of participants with submicroscopic infections having fever (39 substudies, 165 [5·6%] women had fever among 2926 women with submicroscopic infection). As expected, the risk of fever was higher among women with microscopic infections (median 9·0% [0·0–100]) than among women without infections (3·8% [0·0–27·3], adjusted OR [aOR] 2·84, 95% CI 2·30–3·51; p<0·0001; appendix p 31). The risk of fever was also higher among women with submicroscopic malaria than among women without infections (1·29, 1·04–1·60; p=0·018), but when analysed by region, this finding was only evident in Africa (1·32, 1·02–1·72; p=0·038). In Asia and the Americas, compared with women without infections, fever was more common among women with microscopic infection (4·07, 2·91–5·69; p<0·0001) but not among women with submicroscopic infections (1·11, 0·76–1·61; p=0·59; appendix p 31).

Conversely, among women with fever, the median proportion of participants with submicroscopic malaria was 0·0% (range 0·0–33·3) in the Americas (11 substudies, four [2%] had submicroscopic malaria out of 171 with fever), 2·7% (0·0–34·5) in Asia (12 substudies, 34 [6%] had submicroscopic malaria out of 578 with fever), and 25·3% (0·0–100) in Africa (17 substudies, 127 [25%] had submicroscopic malaria out of 513 with fever).

14 (31%) of 45 studies that provided IPD reported species-specific results based on PCR, including five studies from Africa. In Africa, the proportion of infections due to *P falciparum* during pregnancy was similar among microscopic (median 98·1%, range 84·6–100) and submicroscopic infections (94·9%, 76·9–100; appendix p 32). In Asia and the Americas, *P falciparum* was responsible for a median of 75% (range 0·0–100) microscopic infections and 52·3% (0·0–100) submicroscopic infections during pregnancy. In Asia and the Americas, the risk that an infection during pregnancy was submicroscopic and not microscopic was higher for *P vivax* (aOR 3·69, 95% CI 2·45–5·54; p<0·0001) or other monoinfections (7·86, 2·92–21·15; p<0·0001) than for *P falciparum* monoinfection (appendix p 33). Similar results were seen in the peripheral blood at delivery for *P vivax* (appendix p 33).

Microscopic infections were more prevalent than no infection in younger women (age <30 years) and in paucigravidae (ie, primigravidae and secundigravidae) in Africa (at any transmission level) but not in Asia (table 2). Microscopic infections were also more common than no infection in the rainy season compared with the dry season in high transmission areas in Africa (aOR 1·30, 95% CI 1·14–1·48).

Submicroscopic infections were more prevalent among younger women (adjusted for gravidity, age <20 *vs* age ≥30 years: aOR 1·59, 95% CI 1·21–2·10), but primigravid women were less likely to have submicroscopic infections than multigravid women (adjusted for age, primigravidae *vs* multigravidae: 0·65, 0·51–0·83) in high transmission areas in Africa. By contrast, in moderate-to-low transmission areas in Africa, both women younger than 20 years (1·94, 1·54–2·45) and primigravid women (1·23, 1·01–1·51) had an increased risk of submicroscopic malaria infection.

Among participants with NAAT-positive infections, women younger than 20 years were less likely to carry submicroscopic infections than women 30 years and older in all regions (aOR 0·49, 95% CI 0·37–0·66 for women aged <20 years in high transmission areas *vs* 0·59, 0·41–0·85 for women younger than 20 years in moderate-to-low transmission areas); the same pattern was seen for primigravid women in Africa (0·35, 0·28–0·45 for high transmission areas *vs* 0·51, 0·38–0·68 for moderate-to-low transmission areas) and Asia (0·73, 0·51–1·04; table 2).

When results were stratified by gravidity, women younger than 30 years were more at risk for microscopic infections than women who were 30 years and older in high transmission areas in Africa for all gravida groups, whereas women younger than 20 years in their second pregnancy were more at risk than women who were 30 years and older in moderate-to-low transmission areas in Africa and in Asia and the Americas (appendix p 34). First antenatal clinic visit was a protective factor for malaria infections in Africa whereas it was associated with a higher risk of malaria infection in the Americas and Asia (table 2). However, this finding could be a distortion: in high transmission areas in Africa, the only study that included first antenatal clinic visits included women in their first and second pregnancy only. The only studies outside of Africa that included first antenatal clinic visits were two studies in Asia with a higher malaria prevalence compared with the other studies.

When assessing predictors by species in Asia and the Americas, for *P falciparum*, microscopic infections were more common among women in their first and second pregnancy than women in their third pregnancy or more (aOR 1·39, 95% CI 0·95–2·04), whereas for *P vivax*, microscopic infections were less common among women in their first or second pregnancy (0·54, 0·27–1·09; appendix p 37). Subgroup analysis for first antenatal clinic visits only, or the effect of inclusion of variables with a more restricted sample size (eg, HIV, gestational age, and antimalarial use) on the models, and for models at delivery are shown in the appendix (pp 38–48).

Nine cohort studies (five of which were trials) provided information on submicroscopic malaria at a consecutive scheduled follow-up visit; seven (78%) in Africa and two (22%) in Asia and the Americas (appendix pp 50, 62–65). In Asia and the Americas combined, only one (1%) of 70 women with submicroscopic infection at enrolment presented with a submicroscopic infection at the subsequent visit (median 0·0% [range 0·0–11·1], interval 27 days; appendix p 66) whereas no women had microscopic infections (≥95% used an antimalarial). In Africa, among 1009 women with a submicroscopic infection at enrolment, a median of 18·5% (range 7·7–56·0; median interval 50 days [range 14–125]) presented with submicroscopic infections at the subsequent visit, and 8·5% (range 0·0–37·7, median interval 28 days [range 14–101]) presented with microscopic infections at the subsequent visit. A median of 76·6% (range 0·0–95·0 by sublocation) of women received an antimalarial at enrolment (receipt of sulfadoxine–pyrimethamine, median 36·8% [range 0·0–54·2]).

Factors associated with submicroscopic and microscopic malaria at the consecutive visit after submicroscopic malaria at enrolment were high sulfadoxine–pyrimethamine resistance and transmission level in the region (submicroscopic malaria at subsequent visit, aOR 2·36, 95% CI 1·04–5·36 for high sulfadoxine–pyrimethamine resistance in moderate-to-low transmission areas, and 28·72, 9·67–85·29 for high sulfadoxine–pyrimethamine resistance in high transmission areas compared with high transmission areas with low sulfadoxine–pyrimethamine resistance; table 3). Submicroscopic malaria at enrolment and also at subsequent visits was seen more frequently in cohort studies than in trials (table 3). Submicroscopic malaria at subsequent visits was less likely in women who received dihydroartemisinin–piperaquine at enrolment compared with women who received other or no treatment (0·44, 0·26–0·72). Rainy season at enrolment visit was a risk factor for microscopic malaria at subsequent visits after submicroscopic infection at enrolment (table 3).

Results among aggregated data and IPD did not show significant differences, except for the proportion of submicroscopic malaria among women with NAAT-positive tests when testing maternal blood at delivery (median 77·2% [range 32·6–100] for IPD, n=3631 *vs* 53·7% [16·7–100] for aggregated data, n=797; appendix p 50). Studies of higher quality in Asia were less likely to report submicroscopic malaria than studies in Asia with lower quality, but no other associations were noted by study quality in multivariable models (appendix p 51). Further results of sensitivity analyses are provided in the appendix (pp 12, 53–57).

## Discussion

In this systematic review and meta-analysis of 68 studies, including 45 IPD and 23 aggregated datasets, most NAAT-detected infections were submicroscopic, ranging from 59% during pregnancy to 71–74% at delivery. Infections were more likely to be submicroscopic in Asia and the Americas than in Africa. Submicroscopic infections were predictive of future episodes of submicroscopic or microscopic infections in areas of high malaria transmission and high sulfadoxine–pyrimethamine resistance. Submicroscopic infections were also associated with fever in Africa, albeit less than among women with microscopic malaria.

In Africa, consistent with previous observations among non-pregnant populations,^20^ the proportion of submicroscopic malaria in pregnancy decreased with increasing transmission intensity. The higher proportion of submicroscopic malaria in pregnancy among individuals with malaria infections in lower transmission settings in Africa and in all settings in Asia and the Americas compared with high transmission areas in Africa is consistent with earlier studies among non-pregnant populations in low-endemic settings.^20,21^ Factors proposed to explain why parasite densities in non-pregnant populations are lower in low transmission settings could also apply to pregnant women. These factors are likely to be complex^19,21^ and could include differences in the predominant species, with *P vivax* and other non-falciparum species typically having lower parasite densities than *P falciparum*;^19^ the lower genetic diversity of parasite populations, enabling individuals to rapidly acquire immunity; and low infectious biting rates among *Anopheles* mosquitoes reducing the chance of superinfection.^19,21,22^ Submicroscopic infections have been reported to be more likely in settings that have had a decline of malaria in the past 15 years than in settings where malaria is stable, probably due to the persistence of previously acquired immunity.^21^ Parasite characteristics such as low virulence might also contribute if they result in fewer clinical manifestations, making diagnosis and effective treatment of these infections less likely and resulting in chronic infections and a transmission advantage.^22^ Others have suggested zoonotic transmission of malaria outside of established malaria transmission areas as a reason for submicroscopic malaria in pregnancy.^23^

It is generally believed that in areas of low or unstable malaria transmission, such as in most of Asia and the Americas, where pregnant women have acquired less immunity than in higher transmission areas, infected women are more likely to develop microscopic and symptomatic malaria or severe disease when infected.^24^ This belief is not supported by our findings. Indeed, in areas of high malaria transmission in Africa, women with submicroscopic infections were more likely to have fever than women with submicroscopic infections in Asia and the Americas or women living in areas of low malaria transmission in Africa. Higher parasite densities have been associated with clinical fever^25^ but submicroscopic infections generally have low parasite densities. However, by PCR, submicroscopic parasite densities were higher in high transmission areas than in areas of lower transmission.^19^ Submicroscopic infections have been associated with fever among children aged 2–10 years in high-malaria transmission areas of Uganda.^26^ It is possible that in moderate-to-high transmission areas, the more frequent exposure to infected mosquito bites results in malaria superinfections that fluctuate around the amount required for microscopic detection, whereas more virulent and new genetic strains could result in fever.

Women younger than 30 years and primigravid women were least likely to have submicroscopic densities when infected, as reported previously.^27^ This finding concurs with observations from a cohort study of individuals who were not pregnant in Malawi, in whom persistent submicroscopic infections were more common and longer lasting among older people.^28^ Malaria infections in women with HIV infection were also more likely to be detectable by microscopy. HIV is known to affect the development of immunity to malaria in pregnancy.^29^

The main advantage of IPD analysis is greater analytical flexibility. In IPD analysis, the effect of gravidity and age could be explored in greater depth than in an aggregated meta-analysis. The analyses included studies from all endemic regions and all parasite species, and the sample size was considerable. Of 68 studies, the majority were from Africa (54 [79%]), and 39 (57%) studies contributed cross-sectional data, which might have been more likely to capture infections of longer duration compared with cohort studies. There are several limitations to our study. 19 (22%) of 87 eligible studies identified did not contribute to the analyses, which could have introduced bias. The majority of participants came from trials and these participants might not represent women in real-life conditions because they could benefit from frequent follow-up, early detection, and prompt treatment of malaria infection.^30^ Additionally, PCR tests can overestimate parasite prevalence for a few weeks after treatment.^10^ Limits of detection for molecular methods of participating studies were generally not available. Furthermore, even though this analysis involved 68 studies, the numbers of participants in some subgroups were still low (eg, by species and for fever). Because of high heterogeneity when pooling prevalence data, we also presented the median and range; median and pooled estimates were generally similar (eg, table 1). Availability of follow-up information on submicroscopic malaria was scarce, with variation in follow-up schedules; there was no molecular confirmation at follow-up visits to discriminate new from persisting infections. NAAT and microscopy methods differed between studies, and there was a paucity of quantitative data on parasite density. Additionally, some covariates were only available in a small number of studies (eg, HIV infection) or were measured or defined differently across studies (eg, use of antimalarials or fever), restricting their use to broad categories only. The number of studies with information on submicroscopic malaria in low transmission areas in Africa was low (three in pregnancy, and two and three at delivery for women with maternal and placental blood testing, respectively), limiting the analyses that could be conducted. Finally, the analysis of microscopic infections was restricted to studies that assessed both microscopic and submicroscopic infections.

This systematic review and meta-analysis showed that NAAT-positive infections during pregnancy and at delivery were more likely to be submicroscopic than microscopic. Submicroscopic infections were more often seen in women with malaria infection in Asia and the Americas, where malaria control in pregnancy mainly relies on screening pregnant women at the first antenatal clinic visit. Thus, many of these low-density infections will not be detected and remain untreated and could contribute to the onward transmission of malaria to mosquitoes.^13,19,31^ The use of more sensitive diagnostic methods could increase the proportion of women with low-density infection receiving appropriate treatment and eventually reduce the transmission reservoir. Further analysis of submicroscopic malaria is needed to better understand the effect of these infections on adverse pregnancy outcomes.^32–34^

## Supplementary Material

Supplement

## Figures and Tables

**Table 1:** Summary estimates for prevalence and proportion of submicroscopic and microscopic malaria infection using individual participant data and aggregated data

	Microscopic malaria infection					Submicroscopic malaria infection			Proportion with submicroscopic malaria infection among participants with NAAT-positive infections				
	Substudies, N*	Study participants, N	Pooled estimate†, % (95% Cl)	*I*^2^, %	Median, % (range)	Pooled estimate†, % (95% Cl)	*I*^2^, %	Median, % (range)	Substudies, N*	Study participants, N	Pooled estimate†, % (95% Cl)	*I*^2^, %	Median, % (range)
**During pregnancy**													
Overall	66	31934	9·(6·9–13·0)	98·8	8·0 (0·0–50·6)	14·6 (12·1–17·4)	97·7	13·5 (0·0–55·9)	66	8983	62·5 (56·5–68·3)	96·6	58·7 (0·0–100)
By region													
The Americas	11	2683	1·6 (0·7–2·9)	76·2	2·2 (0·0–4·7)	6·1 (2·6·10·9)	94·9	6·0 (0·0–35·1)	11	246	72·5 (49·8–91·1)	90·5	73·3 (0·0–100)
Asia	13	10 144	4·5 (2·0·7·9)	97·8	4·1 (0·0–32·9)	10·5 (6·8·14·9)	97·5	9·8 (2·2–35·6)	13	1366	73·6 (61·2–84·5)	95·3	67·2 (36·4–100)
Africa	42	19 107	14·9 (11·1·19·1)	98·4	12·8 (1·3–50·6)	18·8 (15·5–22·2)	97·1	18·6 (2·0–55·9)	42	7371	56·5 (49·9–62·9)	96·6	56·5 (20·5–97·7)
Africa, by transmission intensity													
Low (P*f*PR_2–10_ <10%)	4	1009	6·8 (3·4·11·2)	79·4	7·9 (2·0–12·0)	9·6 (2·5–20·3)	94·9	7·8 (2·0–28·2)	4	159	56·4 (42·9–69·5)	55·6	50·0 (48·5–70·2)
Moderate (P*f*PR_2–10_ 10–34%)	28	10 827	10·7 (8·3·13·3)	94·2	11·9 (1·3–25·8)	19·7 (15·2–24·7)	97·4	19·8 (4·6–55·9)	28	3208	63·6 (55·9–70·9)	94·8	61·0 (26·9–97·7)
High (P*f*PR_2–10_ ≥ 35%)	10	7271	33·9 (27·2·40·9)	97·3	32·6 (13·3–50·6)	20·2 (15·6–25·2)	95·9	19·0 (11·7–31·3)	10	4004	37·6 (31·0–44·4)	94·4	38·5 (20·5–49·0)
**Delivery, maternal peripheral blood**													
Overall	72	26 264	4·0 (2·9·5·4)	96·0	2·9 (0·0–42·9)	10·1 (8·0·12·4)	97·0	7·8 (0·0–57·1)	70	4428	72·3 (67·1–77·2)	91·2	73·6 (16·7–100)
By region													
The Americas	10	1777	0·9 (0·0–8)	86·7	0·0 (0·0–12·6)	6·2 (2·4·11·6)	93·5	5·1 (0·0–36·8)	8	176	89·2 (70·2–99·8)	87·2	90·9 (35·3–100)
Asia	10	5264	2·0 (0·9·3·5)	90·1	1·9 (0·0–9·4)	7·8 (4·2·12·3)	96·8	6·4 (1·9–35·9)	10	518	78·1 (64·4–89·5)	90·3	78·5 (43·3–100)
Africa	52	18 863	5·3 (37·7·2)	96·2	3·9 (0·0–42·9)	11·4 (8·8·14·3)	97·2	8·8 (0·5–57·1)	52	3734	68·5 (62·7–73·9)	90·9	67·9 (16·7–100)
Africa, by transmission intensity													
Low (P*f*PR_2–10_ <10%)	2	754	1·7 (0·9–2·8)	NA	1·8 (1·7–1·8)	1·8 (0·9–2·9)	NA	2·0 (1·2–2·7)	2	27	51·9 (32·4–71·2)	NA	50·8 (41·7–60·0)
Moderate (P*f*PR_2–10_ 10–34%)	37	11 245	3·6 (2·5–4·9)	90·3	3·2 (0·0–21·4)	10·1 (7·6–12·9)	95·4	8·7 (0·5–27·8)	37	1715	73·2 (66·8–79·2)	85·1	72·7 (16·7–100)
High (P*f*PR_2–10_ ≥ 35%)	13	6864	12·8 (7·7–19·0)	97·9	11·0 (1·5–42·9)	17·9 (10·8–26·3)	98·5	18·7 (3·1–57·1)	13	1992	58·2 (48·1–67·9)	94·4	57·1 (32·6–82·9)
**Delivery, placental blood**													
Overall	70	21 478	3·5 (2·1–5·2)	96·8	2·5 (0·0–35·2)	9·0 (7·1–11·1)	95·8	7·5 (0·0–49·5)	66	3753	72·7 (66·8–78·3)	91·1	70·9 (31·3–100)
By region													
The Americas	10	934	0·0 (0·0–0·9)	59·8	0·3 (0·0·8·4)	4·7 (0·1–13·4)	93·2	4·2 (0·0–49·5)	7	103	91·4 (82·8–97·8)	0·0	85·5 (66·7–100)
Asia	11	4457	1·5 (0·3–3·3)	92·2	1·1 (0·0·15·0)	5·2 (2·4–9·0)	95·3	3·2 (0·0–33·6)	10	313	78·0 (61·3–91·5)	87·0	73·0 (31·3–100)
Africa	49	16 087	5·1 (3·2–74)	97·2	3·4 (0·0·35·2)	10·8 (8·6–13·2)	95·4	10·9 (1·0–35·7)	49	3337	69·6 (63·0–75·9)	92·1	66·7 (39·0–100)
Africa, by transmission intensity													
Low (P*f*PR_2–10_ <10%)	3	924	1·5 (0·7–2·4)	0·0	1·5 (0·6–2·1)	1·6 (0·6–3·1)	50·7	1·2 (1·0–2·9)	3	30	53·5 (33·6–72·9)	0·0	58·8 (40·0–66·7)
Moderate (P*f*PR_2–10_ 10–34%)	33	8545	3·6 (2·4–5·1)	90·1	2·8 (0·1–4·5)	10·2 (7·6–13·1)	94·1	9·0 (1·5–30·8)	33	1285	74·4 (67·9–80·4)	80·4	73·2 (40·0–100)
High (P*f*PR_2–10_ ≥ 35%)	13	6618	11·5 (5·8–18·7)	98·5	8·7 (0·3–35·2)	15·4 (11·5–19·8)	95·2	16·2 (4·6–35·7)	13	2022	61·0 (49·8–71·6)	95·6	59·3 (39·0–97·9)

Substudies included different locations within a study, or groups if stratified enrolment (appendix p 25). Studies were split by location when possible. Studies in Asia and the Pacific were included under Asia; studies in central or South America were included under the Americas. LAMP=loop-mediated isothermal amplification. NA=not applicable due to number of substudies being too low for an *I*^2^. NAAT=nucleic acid amplification test (PCR or LAMP). *Pf*PR_2–10_=*Plasmodium falciparum* prevalence among children aged 2–10 years at the year of study visit, as estimated by the Malaria Atlas Project.

* Because some studies did not have submicroscopic or microscopic malaria, the number of substudies do not always match between this column and the second column of substudies.

† Data were pooled using random effects meta-analysis in metaprop (Stata).

**Table 2:** Multivariable analyses of factors associated with malaria infections in pregnancy by type of infection and region, using individual participant data

	Available data, %	Any malaria infection *vs* no malaria		Microscopic infection *vs* no malaria		Submicroscopic infection *vs* no malaria		Risk of submicroscopic infections among women with NAAT-positive infections	
		aOR (95% CI)	p value	aOR (95% CI)	p value	aOR (95% CI)	p value	aOR (95% CI)	p value
Africa, high transmission intensity* **(base model, N=6351, 94·1% of available data, 8 sublocations**^†^**)**									
Age, years									
<20	94·1%	2·43 (1·96–3·00)	<0·0001	3·24 (2·53–4·15)	<0·0001	1·59 (1·21–2·10)	0·0009	0·49 (0·37–0·66)	<0·0001
20–29	94·1%	1·34 (1·14–1·57)	0·0003	1·64 (1·35–2·01)	<0·0001	1·13 (0·94–1·36)	0·20	0·69 (0·55–0·86)	0·0008
≥30	94·1%	1 (ref)	..	1 (ref)	..	1 (ref)	..	1 (ref)	..
Gravidity									
Primigravidae	94·1%	1.24 (1·03–1·49)	0·026	1·84 (1·50–2·27)	<0·0001	0·65 (0·51–0·83)	0·0007	0·35 (0·28–0·45)	<0·0001
Secundigravidae	94·1%	0·95 (0·80–1·11)	0·50	1·21 (1·00–1·46)	0·044	0·73 (0·59–0·90)	0·0035	0·60 (0·49–0·75)	<0·0001
Multigravidae or more	94·1%	1 (ref)	..	1 (ref)	..	1 (ref)	..	1 (ref)	..
P*f*PR_2–10_	94·1%	1·02 (1·00–1·03)	0·0094	1·03 (1·02–1·04)	<0·0001	1·00 (0·99–1·02)	0·64	0·98 (0·97–0·98)	<0·0001
Rainy vs dry season	94·1%	1·18 (1·05–1·33)	0·0072	1·30 (1·14–1·48)	0·0001	0·98 (0·83–1·15)	0·80	0·75 (0·64–0·89)	0·0007
First antenatal clinic visit‡	94·1%	0·50 (0·26–0·98)	0·045	0·50 (0·26–0·97)	0·039	0·48 (0·22–1·04)	0·06	0·96 (0·76–1·21)	0·71
**Africa, high transmission intensity (base model with additional variables of interest with restricted sample size)**									
Gestational age, weeks§	94·4%	1·00 (0·99–1·01)	0·88	1·00 (0·99–1·01)	0·99	1·00 (0·99–1·01)	0·75	1·00 (0·99–1·01)	0·84
HIV infection	58·8%	1·65 (1·13–2·41)	0·010	1·82 (1·17–2·83)	0·0075	1·47 (0·94–2·20)	0·090	0·81 (0·51–1·28)	0·37
Rural setting§	24·3%	1·18 (0·76–1·82)	0·45	1·05 (0·76–1·45)	0·76	1·37 (0·92–2·04)	0·13	1·43 (0·91–2·24)	0·12
Antimalarial use§	20·0%	0·32 (0·23–0·45)	<0·0001	0·27 (0·18–0·40)	<0·0001	0·56 (0·36–0·88)	0·011	2·08 (1·42–3·05)	0·0002
ITN use	695%	0·99 (0·87–1·12)	0·84	1·02 (0·89–1·18)	0·76	0·91 (0·77–1·09)	0·33	0·90 (0·75–1·08)	0·25
Any net use	86·1%	0·94 (0·84–1·06)	0·33	0·97 (0·85–1·10)	0·59	0·90 (0·77–1·04)	0·16	0·96 (0·82–1·12)	0·63
IRS	46.4%	0·98 (0·91–1·04)	0·46	0·95 (0·87–1·03)	0·21	1.00 (0·93–1·08)	0·90	1·06 (0·97–1·16)	0·21
Africa, moderate-to-low transmission intensity* **(base model, N=8199, 98·2% of available data, 24 sublocations**^†^**)**									
Age, years									
<20	98·2%	2·29 (1·87–2·81)	<0·0001	3·31 (2·37–4·62)	<0·0001	1·94 (1·54–2·45)	<0·0001	0·59 (0·41–0·85)	0·0050
20–29	98·2%	1·26 (1·08–1·47)	0·0033	1·62 (1·23–2·14)	0·0007	1·15 (0·97–1·36)	0·11	0·71 (0·52–0·96)	0·0268
≥30	98·2%	1 (ref)	..	1 (ref)	..	1 (ref)	..	1 (ref)	..
Gravidity									
Primigravidae	98·2%	1·58 (1·34–1·88)	<0·0001	2·43 (1·88–3·14)	<0·0001	1·23 (1·01–1·51)	0·039	0·51 (0·38–0·68)	<0·0001
Secundigravidae	98·2%	1·23 (1·06–1·43)	0·0078	1·42 (1·11–1·81)	0·0045	1·15 (0·96–1·36)	0·13	0·81 (0·61–1·06)	0·12
Multigravidae or more	98·2%	1 (ref)	..	1 (ref)	..	1 (ref)	..	1 (ref)	..
P*f*PR_2–10_	98·2%	0·97 (0·95–0·98)	0·0015	0·97 (0·94–1·00)	0·031	0·97 (0·94–0·99)	0·0042	0·99 (0·97–1·02)	0·74
Rainy *vs* dry season	98·2%	0·96 (0·86–1·07)	0·42	0·95 (0·81–1·11)	0·48	0·96 (0·85–1·09)	0·52	1·02 (0·85–1·21)	0·86
First antenatal clinic visit}	98·2%	0·70 (0·37–1·30)	0·25	0·61 (0·32–1·16)	0·13	0·79 (0·39–1·58)	0·50	1·30 (0·70–2·40)	0·40
**Africa, moderate-to-low transmission intensity (base model with additional variables of interest with restricted sample size)**									
Gestational age, weeks	88.7%	0·98 (0·96–0·99)	0·0002	0·97 (0·96–0·99)	0·0043	0·98 (0·96–0·99)	0·0053	1·00 (0·98–1·03)	0·65
HIV infection	90’6%	1·19 (0·84–1·67)	0·32	1·72 (1·04–2·86)	0·036	0·95 (0·63–1·43)	0·80	0·55 (0·30–1·00)	0·049
Rural setting	37·0%	1·41 (0·64–3·13)	0·39	2·58 (1·06–6·29)	0·037	1·13 (0·49–2·61)	0·78	0·44 (0·22–0·87)	0·018
Antimalarial use	17·7%	0·61 (0·25–3·51)	0·015	1·34 (0·80–2·26)	0·27	0·40 (0·23–0·68)	0·0007	0·39 (0·20–0·79)	0·0085
ITN use	14·5%	0·90 (0·63–1·29)	0·57	0·85 (0·49–1·48)	0·57	0·96 (0·62–1·47)	0·85	1·14 (0·59–2·20)	0·70
Any net use	66·3%	0·88 (0·76–1·02)	0·092	0·96 (0·78–1·19)	0·72	0·84 (0·71–1·00)	0·0447	0·87 (0·69–1·11)	0·26
IRS	50·2%	1·14 (0·82–1·58)	0·43	1·13 (0·71–1·82)	0·60	1·15 (0·79–1·68)	0·46	1·02 (0·60–1·71)	0·95
**The Americas and Asia (base model, N=10 068, 97·7% of available data, 22 sublocations** † **)**									
Age, years									
<20	97·7%	1·58 (1·23–2·03)	0·0003	2·76 (1·73–4·39)	<0·0001	1·31 (0·99–1·75)	0·061	0·48 (0·28–0·80)	0·0049
20·29	97·7%	1·18 (0·99–1·42)	0·071	1·66 (1·13–2·43)	0·0093	1·09 (0·90–1·33)	0·37	0·66 (0·44–0·99)	0·046
≥30	97·7%	1 (ref)	..	1 (ref)	..	1 (ref)	..	1 (ref)	..
Gravidity									
Primigravidae	97·7%	0·95 (0·80–1·14)	0·61	1·20 (0·87–1·64)	0·27	0·87 (0·71–1·07)	0·18	0·73 (0·51–1·04)	0·077
Secundigravidae	97·7%	0·99 (0·83–1·19	0·95	1·03 (0·73–1·45)	0·86	0·99 (0·81–1·20)	0·90	0·96 (0·66–1·39)	0·81
Multigravidae or more	97·7%	1 (ref)	..	1 (ref)	..	1 (ref)	..	1 (ref)	..
P*f*PR_2–10_	97·7%	1·04 (1·02–1·07)	0·0014	1·00 (0·96–1·04)	0·90	1·06 (1·03–1·09)	0·0004	1·06 (1·01–1·12)	0·021
Rainy *vs* dry season	97·7%	1·05 (0·90–1·21)	0·56	1·24 (0·96–1·61)	0·10	0·98 (0·83–1·16)	0·83	0·79 (0·59–1·06)	0·11
First antenatal clinic visit}	97·7%	2·73 (0·86–8·68)	0·089	11·15 (2·26–54·97)	0·0031	1·93 (0·49–7·55)	0·35	0·15 (0·02–0·86)	0·034
The Americas *vs* Asia	97·7%	0·87 (0·32–2·32)	0·78	1·57 (0·39–6·31)	0·53	0·71 (0·22–2·29)	0·57	0·38 (0·08–1·84)	0·23
**The Americas and Asia (base model with additional variables of interest with restricted sample size** ¶ **)**									
Gestational age, weeks	96·1%	1·00 (0·99–1·01)	0·51	0·99 (0·97–1·01)	0·17	1·00 (0·99–1·01)	0·98	1·01 (0·99–1·04)	0·22
Rural setting	80·7%	1·46 (1·20–1·76)	<0·001	1·76 (1·25–2·46)	0·0010	1·34 (1·07–1·67)	0·012	0·77 (0·52–1·13)	0·18
Antimalarial use	52·5%	1·24 (0·90–1·70)	0·18	1·35 (0·88–2·09)	0·17	1·17 (0·80–1·72)	0·41	0·87 (0·52–1·45)	0·59
ITN use	53·6%	1·08 (0·86–1·37)	0·50	1·10 (0·78–1·55)	0·58	1·09 (0·83–1·44)	0·52	0·98 (0·65–1·48)	0·94
Any net use	88·2%	1·12 (0·96–1·31)	0·16	1·16 (0·88–1·54)	0·29	1·10 (0·92–1·31	0·29	0·95 (0·69–1·29)	0·73
IRS	36·6%	1·03 (0·76–1·39)	0·86	0·86 (0·39–1·91)	0·71	1·04 (0·75–1·44)	0·80	1·21 (0·52–2·83)	0·66

Multigravidae excluded secundigravidae. Available data refer to proportion of participants with an outcome on submicroscopic, microscopic, and no malaria. Studies in Asia and the Pacific were included under Asia; studies in central or South America were included under the Americas. In the multinomial model, the parameter estimates are relative to the referent group; for a unit change in the covariate the logit of the outcome is expected to change by its respective parameter estimate given the variables in the model are held constant (eg, for the probability of having microscopic malaria during pregnancy in high transmission areas in Africa, one increase in unit of PfPR_2–10_ resulted in an increase of 0·029 logit of microscopic malaria, which translates to an OR of 1·3 [exponent of 0·029]; this OR means that for 1 percentage point of increase in transmission there is a 3% increase in the odds of having microscopic malaria among pregnant women). aOR=adjusted OR. ITN=insecticide treated net. IRS=indoor residual spraying. LAMP=loop-mediated isothermal amplification. NAAT=nucleic acid amplification test (PCR or LAMP). OR=odds ratio. *Pf*PR_2–10_=*Plasmodium falciparum* prevalence among children aged 2–10 years at the year of study visit, as estimated by the Malaria Atlas Project.

* Africa, high transmission: PfPR_2–10_ ≥35%; Africa, moderate-to-low transmission: PfPR_2–10_ <35% (only 1 study [two sublocations] had PfPR_2–10_ <10%). In the model for Asia and the Americas, one study in Indonesia (9% of data), had PfPR_2–10_ of 25%, all other studies had a PfPR_2–10_ in the range of 0–5%, with 81% of studies having PfPR_2–10_ of <2%. PfPR_2–10_ was added as a continuous variable in all models.

† Available data (data with information on microscopic infection, submicroscopic infection, and no malaria infection) in Africa, in high transmission areas: N=6746, nine sublocations (microscopic infection n=2455 [36·3%], submicroscopic infection n=1406 [20·8%], and no malaria infection n=2885 [42·8%]); in Africa, in moderate-to-low transmission areas: N=8350, 25 sublocations (microscopic infection n=949 [11·4%], submicroscopic infection n=1858 [22·3%], and no malaria infection n=5543 [66·4%]); and in the Americas and Asia: N=10 305, 23 sublocations (microscopic infection n=373 [3·6%], submicroscopic infection n=919 [8·9%], and no malaria infection n=9013 [87·5%]).

‡ Comparison group for first antenatal clinic visit was not first antenatal clinic visit or unknown whether first visit or not. For Africa, in high transmission areas, the protective effect of first antenatal clinic visit for microscopic malaria comes from one study that only included women in their first or second pregnancy compared with six studies in which visits were not first antenatal clinic visit, or this information was unknown. Exclusion of first antenatal clinic visits from this model did not result in meaningful changes for the other covariates (data not shown).

§ In Africa, in high transmission areas, models for gestational age were run without first antenatal clinic visit because of non-convergence; *Pf*PR_2–10_ was not included in the model for submicroscopic *vs* microscopic malaria. In Africa, in high transmission areas: models for setting and antimalarial use did not include first antenatal clinic visit because all involved studies included women at any antenatal clinic visit, or it was unknown. *Pf*PR_2–10_ was not included in the models of antimalarial use, rural setting, and any net use because of non-convergence.

¶ There was insufficient information on HIV infection in studies in the Americas and Asia.

**Table 3:** Factors in studies in Africa associated with presence of submicroscopic malaria over two scheduled study visits or transition of submicroscopic malaria into microscopic malaria at a consecutive scheduled study visit, 2010–17

	Factors associated with submicroscopic infection at enrolment and at subsequent study visit*		Factors associated with submicroscopic infection at enrolment and microscopic infection at subsequent study visit*	
	aOR (95% CI)	p value	aOR (95% CI)	p value
Age	1·01 (0·97–1·05)	0·55	0·95 (0·90–1·00)	0·074
Gravidity				
Primigravidae	1·51 (0·96–2·35)	0·072	1·31 (0·72–2·36)	0·38
Multigravidae (secundigravidae or more)	1 (ref)	..	1 (ref)	..
Sulfadoxine–pyrimethamine resistance markers and transmission rate†				
High transmission, low sulfadoxine–pyrimethamine resistance	1 (ref)	..	1 (ref)	..
Moderate-to-low transmission, high sulfadoxine–pyrimethamine resistance	2·36 (1·04–5·36)	0·040	0·49 (0·24–1·02)	0·056
High transmission, high sulfadoxine–pyrimethamine resistance	28·72 (9·67–85·29)	<0·0001	21·04 (8·07–54·83)	<0·0001
Rainy season *vs* dry	0·95 (0·68–1·33)	0·78	1·79 (1·14–2·81)	0·011
Study design				
Cohort study	4·68 (2·15–10·17)	0·0001	3·37 (1·27–8·95)	0·015
Trial	1 (ref)	..	1 (ref)	..
Antimalarial use reported at enrolment				
None or unknown	1 (ref)	..	1 (ref)	..
Sulfadoxine–pyrimethamine	0·81 (0·52–1·25)	0·35	1·04 (0·58–1·89)	0·89
Dihydroartemisinin–piperaquine	0·44 (0·26–0·72)	0·0011	0·68 (0·36–1·30)	0·24

High sulfadoxine-pyrimethamine resistance: Lys540Glu ≥30% in eastern and southern Africa and Ala437Gly ≥90% in central and western Africa; low sulfadoxine–pyrimethamine resistance: Ala437Gly <90% in central and western Africa, or Lys540Glu <30% in eastern and southern Africa. High malaria transmission: *Pf*PR_2–10_ ≥35%. Moderate-to-low malaria transmission: *Pf*PR_2–10_ <35%. The corresponding aORs when using high transmission and high sulfadoxine–pyrimethamine resistance regions as baseline for submicroscopic malaria were 0·03 (95% CI 0·01–0·11; p<0·0001) for high transmission and low sulfadoxine–pyrimethamine resistance areas and 0·08 (0·04–0·19; p<0·0001) for moderate-to-low transmission and high sulfadoxine–pyrimethamine resistance areas. The corresponding aORs when using high transmission and high sulfadoxine–pyrimethamine resistance areas as baseline for microscopic malaria were 0·05 (0·02–0·13; p<0·0001) for high transmission and low sulfadoxine–pyrimethamine resistance areas and 0·02 (0·01–0·05; p<0·0001) for moderate-to-low transmission and high sulfadoxine–pyrimethamine resistance regions. For repeated submicroscopic malaria and submicroscopic malaria developing into microscopic malaria infection, the reference was no malaria at the consecutive scheduled study visit. Five studies had information on fever at the subsequent scheduled study visit (815 participants): fever was not associated with microscopic or submicroscopic infection (OR 1·19, 95% CI 0·45–3·16; p=0·73 and 0·93, 0·42–2·06; p=0·87, respectively, no malaria infection as reference). aOR=adjusted odds ratio. IPTp=intermittent preventive treatment in pregnancy. OR=odds ratio. *Pf*PR_2–10_=*Plasmodium falciparum* prevalence among children aged 2–10 years at the location and year of study visit, as estimated by the Malaria Atlas Project.

* This analysis included 1009 participants in Africa from seven studies with submicroscopic malaria at enrolment (276 [27·4%] participants with submicroscopic infections and 126 [12·5%] participants with microscopic infections at the consecutive scheduled study visit, *Pf*PR_2–10_ range 11–74%); two cohorts with IPTp with sulfadoxine–pyrimethamine provision, one cohort before IPTp policy, and four trials (IPTp with sulfadoxine–pyrimethamine group and alternative groups).

† All locations in moderate-tolow transmission areas were in areas of high sulfadoxine–pyrimethamine resistance; for this reason, a combination variable of sulfadoxine–pyrimethamine resistance and transmission rate was created, indicating low-to-moderate-transmission and high sulfadoxine–pyrimethamine resistance, high transmission and low sulfadoxine–pyrimethamine resistance, and high-transmission and high sulfadoxine–pyrimethamine resistance.

## Research in context

### Evidence before this study

We conducted a literature search for studies reviewing submicroscopic malaria infection among pregnant women in malarious areas with no language restriction using the search terms (“Polymerase chain reaction” OR “PCR” OR “submicroscopic” OR “sub-microscopic” OR “sub-patent” OR “sub-microscopic” OR “Lamp” OR “loop-mediated isothermal amplification”) AND “malaria” AND “Review” AND “pregnan*”. The following databases were searched: PubMed and the Malaria in Pregnancy Library, which consists of references from Web of Knowledge, Scopus, Cumulative Index to Nursing and Allied Health Literature, Bioline, the Cochrane Library databases, the WHO Global Health Library, grey literature (ie reports, unpublished studies, and theses) and conference abstracts. The initial search was from Jan 1, 1997, to Sept 6, 2020, and updated on Nov 10, 2021. A systematic review among pregnant women of 16 studies in Africa conducted before 2010 reported that a weighted mean of 36% of all infections were submicroscopic; results were not separated by timepoint in pregnancy (pregnancy or at delivery, placental or maternal blood at delivery) and no meta-analysis was presented. Systematic reviews among populations who were not pregnant suggested there is more submicroscopic malaria among older people and fewer submicroscopic infections in the rainy season. Previous meta-analyses on this topic had only been conducted in non-pregnant populations.

### Added value of this study

This pooled analysis of 45 individual participant and 23 aggregated datasets provides further detailed information on the epidemiology of submicroscopic malaria in pregnancy. Submicroscopic malaria in pregnancy occurred in all malarious regions (ie, Asia, the Americas, and Africa) and was more common than microscopic malaria in most settings. Similar to reports among populations who were not pregnant, the proportion of submicroscopic malaria was higher in Asia and the Americas than in Africa, where it was more common among malaria species other than *Plasmodium falciparum*. This study was also able to explore the relationship between malaria infections with fever and found that submicroscopic malaria was associated with fever in Africa, but not in Asia and the Americas. The study additionally explored factors associated with submicroscopic malaria by region and noted that, among women with parasitaemia in Africa, submicroscopic infections were more common in multigravid women and older women (aged ≥30 years) than in primigravid women and younger women (aged <30 years), whereas in Asia and the Americas, only older age was a risk factor. Furthermore, this study also showed a role of sulfadoxine–pyrimethamine resistance in the risk of submicroscopic infections and the effectiveness of antimalarial treatment such as dihydroartemisinin–piperaquine for the reduction of submicroscopic malaria in cohort studies in Africa.

### Implications of all the available evidence

This systematic review and meta-analysis provides new evidence on the extent of submicroscopic malaria in pregnancy and the potential effect of sulfadoxine–pyrimethamine resistance. Submicroscopic infections can contribute to onward malaria transmission, and research is needed on how the burden of submicroscopic malaria can be decreased in women who are pregnant. Studies have suggested that submicroscopic infections could have harmful effects on the mother and infant; further investigation of the role of submicroscopic malaria in pregnancy in causing adverse pregnancy outcomes and potential protective factors in different malaria settings will be important.

## Box

For the **Malaria in Pregnancy Library** see https://mip.wwarn.org

## Box

For the **protocol** see https://www.wwarn.org/tools-resources/subpatent-malaria-study-group-protocol

## Box

See **Online** for appendix

## References

[1] Rogerson SJ, Desai M, Mayor A, Sicuri E, Taylor SM, van Eijk AM. Burden, pathology, and costs of malaria in pregnancy: new developments for an old problem. Lancet Infect Dis 2018; 18: e107–18. 29396010 10.1016/S1473-3099(18)30066-5

[2] Reddy V, Weiss DJ, Rozier J, Ter Kuile FO, Dellicour S. Global estimates of the number of pregnancies at risk of malaria from 2007 to 2020: a demographic study. Lancet Glob Health 2023; 11: e40–47. 36521951 10.1016/S2214-109X(22)00431-4 PMC9764451

[3] Moore KA, Simpson JA, Scoullar MJL, McGready R, Fowkes FJI. Quantification of the association between malaria in pregnancy and stillbirth: a systematic review and meta-analysis. Lancet Glob Health 2017; 5: e1101–12. 28967610 10.1016/S2214-109X(17)30340-6

[4] Moore KA, Simpson JA, Wiladphaingern J, Influence of the number and timing of malaria episodes during pregnancy on prematurity and small-for-gestational-age in an area of low transmission. BMC Med 2017; 15: 117. 28633672 10.1186/s12916-017-0877-6 PMC5479010

[5] Rijken MJ, McGready R, Boel ME, Malaria in pregnancy in the Asia-Pacific region. Lancet Infect Dis 2012; 12: 75–88. 22192132 10.1016/S1473-3099(11)70315-2

[6] Bardaji A, Martinez FE. Epidemiology of malaria during pregnancy: burden and impact of *P. vivax* malaria on maternal and infant health. In: Hommel M, Kremsner PG, eds. Encyclopedia of malaria. New York, USA: Springer, 2015: 1–7.

[7] Mathison BA, Pritt BS. Update on malaria diagnostics and test utilization. J Clin Microbiol 2017; 55: 2009–17. 28404673 10.1128/JCM.02562-16 PMC5483902

[8] Kasetsirikul S, Buranapong J, Srituravanich W, Kaewthamasorn M, Pimpin A. The development of malaria diagnostic techniques: a review of the approaches with focus on dielectrophoretic and magnetophoretic methods. Malar J 2016; 15: 358. 27405995 10.1186/s12936-016-1400-9 PMC4942956

[9] Moody A Rapid diagnostic tests for malaria parasites. Clin Microbiol Rev 2002; 15: 66–78. 11781267 10.1128/CMR.15.1.66-78.2002 PMC118060

[10] Vafa Homann M, Emami SN, Yman V, Detection of malaria parasites after treatment in travelers: a 12-months longitudinal study and statistical modelling analysis. EBioMedicine 2017; 25: 66–72. 29050948 10.1016/j.ebiom.2017.10.003 PMC5704054

[11] Kattenberg JH, Ochodo EA, Boer KR, Schallig HD, Mens PF, Leeflang MM. Systematic review and meta-analysis: rapid diagnostic tests versus placental histology, microscopy and PCR for malaria in pregnant women. Malar J 2011; 10: 321. 22035448 10.1186/1475-2875-10-321 PMC3228868

[12] Walker PGT, Cairns M, Slater H, Modelling the incremental benefit of introducing malaria screening strategies to antenatal care in Africa. Nat Commun 2020; 11: 3799. 32732892 10.1038/s41467-020-17528-3 PMC7393377

[13] Gonçalves BP, Walker PG, Cairns M, Tiono AB, Bousema T, Drakeley C. Pregnant women: an overlooked asset to *Plasmodium falciparum* malaria elimination campaigns? Trends Parasitol 2017; 33: 510–18. 28359609 10.1016/j.pt.2017.03.001

[14] Mayor A, Menéndez C, Walker PGT. Targeting pregnant women for malaria surveillance. Trends Parasitol 2019; 35: 677–86. 31395496 10.1016/j.pt.2019.07.005 PMC6708786

[15] van Eijk AM, Hill J, Povall S, Reynolds A, Wong H, Ter Kuile FO. The Malaria in Pregnancy Library: a bibliometric review. Malar J 2012; 11: 362. 23110589 10.1186/1475-2875-11-362 PMC3522037

[16] Flegg JA, Humphreys GS, Montanez B, Spatiotemporal spread of *Plasmodium falciparum* mutations for resistance to sulfadoxine-pyrimethamine across Africa, 1990–2020. PLoS Comput Biol 2022; 18: e1010317. 35951528 10.1371/journal.pcbi.1010317 PMC9371298

[17] World Wide Antimalarial Resistance Network. Molecular surveyor. 2023. http://www.wwarn.org/dhfr-dhps-surveyor/#0 (accessed Jan 23, 2023).

[18] Malaria Atlas Project. Maps. 2023. https://data.malariaatlas.org/maps?layers=Malaria:202206_Global_Pf_Parasite_Rate (accessed April 5, 2023).

[19] Slater HC, Ross A, Felger I, The temporal dynamics and infectiousness of subpatent *Plasmodium falciparum* infections in relation to parasite density. Nat Commun 2019; 10: 1433. 30926893 10.1038/s41467-019-09441-1 PMC6440965

[20] Okell LC, Bousema T, Griffin JT, Ouédraogo AL, Ghani AC, Drakeley CJ. Factors determining the occurrence of submicroscopic malaria infections and their relevance for control. Nat Commun 2012; 3: 1237. 23212366 10.1038/ncomms2241 PMC3535331

[21] Whittaker C, Slater H, Nash R, Global patterns of submicroscopic *Plasmodium falciparum* malaria infection: insights from a systematic review and meta-analysis of population surveys. Lancet Microbe 2021; 2: e366–74. 34382027 10.1016/S2666-5247(21)00055-0 PMC8332195

[22] jörkman A, Morris U. Why asymptomatic *Plasmodium falciparum* infections are common in low-transmission settings. Trends Parasitol 2020; 36: 898–905. 32855077 10.1016/j.pt.2020.07.008

[23] Hristov AD, Sanchez MC, Ferreira JJ, Malaria in pregnant women living in areas of low transmission on the southeast Brazilian coast: molecular diagnosis and humoural immunity profile. Mem Inst Oswaldo Cruz 2014; 109: 1014–20. 25494466 10.1590/0074-0276140229 PMC4334161

[24] Desai M, ter Kuile FO, Nosten F, Epidemiology and burden of malaria in pregnancy. Lancet Infect Dis 2007; 7: 93–104. 17251080 10.1016/S1473-3099(07)70021-X

[25] Oakley MS, Gerald N, McCutchan TF, Aravind L, Kumar S. Clinical and molecular aspects of malaria fever. Trends Parasitol 2011; 27: 442–49. 21795115 10.1016/j.pt.2011.06.004

[26] Katrak S, Nayebare P, Rek J, Clinical consequences of submicroscopic malaria parasitaemia in Uganda. Malar J 2018; 17: 67. 29402282 10.1186/s12936-018-2221-9 PMC5800031

[27] Walker-Abbey A, Djokam RR, Eno A, Malaria in pregnant Cameroonian women: the effect of age and gravidity on submicroscopic and mixed-species infections and multiple parasite genotypes. Am J Trop Med Hyg 2005; 72: 229–35. 15772312

[28] Buchwald AG, Sorkin JD, Sixpence A, Association between age and *Plasmodium falciparum* infection dynamics. Am J Epidemiol 2019; 188: 169–76. 30252032 10.1093/aje/kwy213 PMC6321803

[29] González R, Ataíde R, Naniche D, Menéndez C, Mayor A. HIV and malaria interactions: where do we stand? Expert Rev Anti Infect Ther 2012; 10: 153–65. 22339190 10.1586/eri.11.167

[30] Walker PG, Griffin JT, Cairns M, A model of parity-dependent immunity to placental malaria. Nat Commun 2013; 4: 1609. 23511473 10.1038/ncomms2605 PMC3615483

[31] Andolina C, Rek JC, Briggs J, Sources of persistent malaria transmission in a setting with effective malaria control in eastern Uganda: a longitudinal, observational cohort study. Lancet Infect Dis 2021; 21: 1568–78. 34146476 10.1016/S1473-3099(21)00072-4 PMC8554388

[32] Cottrell G, Moussiliou A, Luty AJ, Submicroscopic *Plasmodium falciparum* infections are associated with maternal anemia, premature births, and low birth weight. Clin Infect Dis 2015; 60: 1481–88. 25694651 10.1093/cid/civ122

[33] Mockenhaupt FP, Rong B, Till H, Submicroscopic *Plasmodium falciparum* infections in pregnancy in Ghana. Trop Med Int Health 2000; 5: 167–73. 10747278 10.1046/j.1365-3156.2000.00532.x

[34] Adegnika AA, Verweij JJ, Agnandji ST, Microscopic and submicroscopic *Plasmodium falciparum* infection, but not inflammation caused by infection, is associated with low birth weight. Am J Trop Med Hyg 2006; 75: 798–803. 17123968

