# Supplementary material for: Prevalence of and risk factors for microscopic and submicroscopic malaria infections in pregnancy: a systematic review and meta-analysis
Source: Lancet Glob Health. Author manuscript; Available in PMC 2024 Jul 1. (PMC10880462; doi:10.1016/S2214-109X(23)00194-8)
Supplement: Supplement [file NIHMS1965929-supplement-Supplement.pdf]

THE LANCET
Global Health

Supplementary appendix

This appendix formed part of the original submission and has been peer reviewed. We post it as supplied by the authors.

Supplement to: van Eijk AM, Stepniewska K, Hill J, et al. Prevalence of and risk factors for microscopic and submicroscopic malaria infections in pregnancy: a systematic review and meta-analysis. *Lancet Glob Health* 2023; published online June 2. https://doi.org/10.1016/S2214-109X(23)00194-8.

Supplement to: van Eijk et al., Prevalence of and risk factors for microscopic and submicroscopic malaria infections in pregnancy: a systematic review and meta-analyses

Authors

Anna Maria van Eijk, Kasia Stepniewska, Jenny Hill, Professor Steve M. Taylor, Professor Stephen J. Rogerson, Gilles Cottrell, R. Matthew Chico, Julie R. Gutman, Halidou Tinto, Holger W. Unger, Professor Stephanie K. Yanow, Manfred Accrombessi, Professor Ayola A. Adegnika, Rukhsana Ahmed, , Eliana María Arango Flórez, Professor Myriam Arévalo-Herrera, Emmanual Arinaitwe, Paulo Arnaldo, Professor Per Ashorn, Ulla Ashorn, Azucena Bardaji, Inoni Betuela, Praveen K. Bharti, Francis Bohissou, Professor Camila Bôtto-Menezes, Vera Braun, Valerie Briand, Jessica Briggs, Maria Eugenia Castellanos, Professor Daniel Chandramohan, Enesia Banda Chaponda, Professor Chetan E. Chitnis, Laura Cohee, Michel Cot, Professor Umberto d'Alessandro, Lise Denoeud-Ndam, Meghna Desai, Professor Alassane Dicko, Xavier Ding, Professor Grant Dorsey, Patrick E. Duffy, Professor Maha A. Elbadry, Sonia M. Enosse, Yue-Mei Fan, Nadine Fievet, Michal Fried, Professor Blaise Genton, Professor Raquel Gonzalez, Professor Brian Greenwood, Linda Kalilani, Johanna H. Kattenberg, Professor Kassoum Kayentao, Carole Khairallah, Professor Christopher L. King, Professor Dhanpat Kumar Kochar, Professor Swati Kochar, Felix Koukouikila-Koussounda, Sarah H. Landis, Professor Miriam K. Laufer, Professor Rose F. G. Leke, Eusebio Macete, Sonia Maculuve, Mwayiwawo Madanitsa, Almahamoudou Mahamar, Professor Ken Maleta, Indu Malhotra, Rella Zoleko Manego, Professor Flor Ernestina Martínez-Espinosa, Achille Massougbodji, Professor Don Mathanga, Michela Menegon, Professor Clara Menendez, Petra Mens, Professor Martin Meremikwu, Professor Frank P. Mockenhaupt, Professor Ghyslain Mombo-Ngoma, Dominic Mosha, Professor Ivo Müeller, Alain Nahum†, Paul Natureeba, Professor Nicaise Ndam, Professor Francine Ntoumi, Professor Olabisi A. Oduwole, Professor Bernard A. Okech, Maria Ome-Kaius, Kephas Otieno, Norma Padilla, Professor Michael Ramharter, Professor Rosemary Rochford, Professor Anna Rosanas-Urgell, Maria Ruperez, Katherine R. Sabourin, Sergi Sanz, Henk D. Schallig, Susana Scott, Professor Esperanca Sevene, Carlo Severini, Professor Harry Tagbor, Professor Diane Wallace Taylor, Professor Maminata Traore Coulibaly, Professor Ana-Maria Vasquez, Annie Walker-Abbey, Blair J. Wylie, Professor Djimon M. Zannou, Professor Steven R. Meshnick†, Professor Feiko O. ter Kuile, Professor Alfredo Mayor
†Deceased

Affiliations

Department of Clinical Sciences, Liverpool School of Tropical Medicine, Liverpool, Liverpool L3 5QA, UK (A M van Eijk PhD, J Hill PhD, Prof F O ter Kuile PhD, C Khairallah MSc, H W Unger PhD); Centre for Tropical Medicine and Global Health, Nuffield Department of Clinical Medicine, University of Oxford, Oxford, UK (K Stepniewska PhD); Division of Infectious Diseases and Duke Global Health Institute, Duke University, Durham, NC, USA (S M Taylor MD); Department of Infectious Diseases, Doherty Institute, The University of Melbourne, Melbourne, Australia (Prof S J Rogerson PhD); Université de Paris, IRD, MERIT, F-75006 Paris, France (G Cottrell PhD, M Cot MD, N Fievet PhD, Prof N Ndam PhD); London School of Hygiene & Tropical Medicine, London, UK (R M Chico PhD, Prof D Chandramohan PhD, Prof B Greenwood MD, Prof U d'Alessandro PhD, S Scott PhD); Malaria Branch, Division of Parasitic Diseases and Malaria, Center for Global Health, Centers for Disease Control and Prevention (CDC), Atlanta, GA, USA (J R Gutman MD); Institut de Recherche en Sciences de la Santé - Unité de Recherche Clinique de Nanoro, Burkina Faso (Prof H Tinto PhD, Prof M Traore PhD); Menzies School

of Health Research, Charles Darwin University, Darwin, Australia (H W Unger PhD); School of Public Health, Department of Medical Microbiology and Immunology, University of Alberta, Edmonton, Alberta, Canada (Prof S K Yanow PhD); Institut de Recherche Clinique du Benin, Abomey-Calavi, Benin (M Accrombessi MD); Centre de Recherches Médicales de Lambaréné, Lambaréné, Gabon (Prof A A Adegnika PhD, R Z Manego MD, Prof G Mombo-Ngoma PhD); Eijkman Institute for Molecular Biology, Jakarta, Indonesia (R Ahmed PhD); Grupo Salud y Comunidad, Grupo Malaria Facultad de Medicina, Universidad de Antioquia, Medellin, Colombia (E M Arango-Flórez PhD, Prof A-M Vasquez PhD); Caucaseco Scientific Research Center/Universidad del Valle, Cali, Colombia (Prof Myriam Arévalo-Herrera PhD); Infectious Diseases Research Collaboration, Kampala, Uganda (E Arinaitwe PhD); Instituto Nacional de Saúde, Maputo, Mozambique (P Arnaldo PhD, S M Enosse PhD); Center for Child, Adolescent and Maternal Health Research, Faculty of Medicine and Health Technology, Tampere University, Tampere, Finland (Prof P Ashorn PhD, U Ashorn PhD, Y Fan PhD); ISGlobal, Barcelona Institute for Global Health, Hospital Clínic-Universitat de Barcelona, Barcelona, Spain (A Bardaji PhD, Prof A Mayor PhD, Prof R Gonzalez PhD, Prof C Menendez PhD, M Ruperez Phd, S Sanz MSc); Papua New Guinea Institute of Medical Research, Madang, Papua New Guinea (I Betuela MD, M Ome-Kaius PhD); ICMR-National Institute of Malaria Research, New Delhi, India (P K Bharti PhD); Centre de Recherche Entomologique de Cotonou, Cotonou, Benin (F Bohissou MD, A Nahum PhD); Tropical Medicine Foundation Dr. Heitor Vieira Dourado, Manaus, Brazil (Prof C Bôtto-Menezes PhD, F E Martinez-Espinosa); Institute of Tropical Medicine and International Health, Charité-University Medicine Berlin, Berlin, Germany (V Braun PhD, Prof F P Mockenhaupt MD); IRD, Inserm, Université de Bordeaux, GHiGS team, UMR 1219, Bordeaux, France (V Briand MD); Department of Medicine, University of California, San Francisco, CA, USA (J Briggs MD, Prof G Dorsey MD); Public Health and Tropical Medicine, College of Public Health, Medical and Veterinary Sciences, James Cook University, Townsville, Queensland, Australia (M E Castellanos PhD); Department of Biological Sciences, University of Zambia, Lusaka, Zambia (E B Chaponda PhD); Malaria Parasite Biology and Vaccines, Department of Parasites & Insect Vectors, Institut Pasteur, Paris, France (Prof C Chitnis PhD); Center for Vaccine Development and Global Health, University of Maryland School of Medcine, Baltimore, MD USA (L Cohee MD, Prof M K Laufer MD); Epicentre, Paris, France (L Denoeud-Ndam MD); U.S. Centers for Disease Control and Prevention, New Delhi, India (M Desai PhD); Malaria Research & Training Center, Faculty of Medicine, Pharmacy and Dentistry, University of Sciences Techniques and Technologies of Bamako, Bamako, Mali (Prof A Dicko MD, A Mahamar PhD); FIND, Geneva, Switzerland (X Ding PhD); National Institute of Allergy and Infectious Diseases, Bethesda, United States (P E Duffy MD, M Fried PhD, A Walker-Abbey PhD); Department of Environmental and Global Health and Emerging Pathogens Institute, University of Florida, Gainesville, Florida, USA (Prof M A Elbadry PhD, Prof B A Okech PhD); Swiss Tropical and Public Health Institute, Basel, Switzerland (Prof B Genton PhD); College of Medicine, University of Malawi, Blantyre, Malawi (L Kalilani PhD); Department of Biomedical Sciences, Institute of Tropical Medicine Antwerp, Antwerp, Belgium (J H Kattenberg PhD, Prof A Rosanas-Urgell PhD); University of Sciences, Techniques, and Technologies of Bamako, Bamako, Mali (Prof K Kayentao PhD); Center for Global Health and Diseases, Department of Pathology, Case Western Reserve University School of Medicine, Cleveland, Ohio, USA (Prof C L King PhD, I Malhotra PhD); Sardar Patel Medical College and Associated Group of Hospitals, Bikaner, Rajasthan, India (Prof D K Kochar MD, Prof S Kochar MD); Fondation Congolaise pour la Recherche Médicale, Brazzaville, République du Congo (F Koukouikila-Koussounda PhD); Epidemiology and Real World Evidence, Biomarin, London, UK (M K Landis PhD); Faculty of Medicine and Biomedical Sciences, University of Yaounde 1, Yaounde, Cameroon (Prof R F G Leke PhD); Centro de Investigação em Saúde de Manhiça, Maputo, Mozambique (E Macete PhD, S Maculuve MD, Prof E Sevene PhD, Prof A Mayor PhD); Department of Clinical Sciences, Malawi University of Science and Technology, Thyolo, Malawi (M Madanitsa PhD);

School of Global and Public Health, Kamuzu University of Health Sciences, Blantyre, Malawi (Prof K Maleta PhD, Prof D Mathanga PhD); Leônidas & Maria Deane Institute FIOCRUZ, Manaus, Brazil (Prof F E Martinez-Espinosa PhD); Faculté des Sciences de la Santé, Université d'Aboméy Calavi, Cotonou, Benin (A Massougbodji PhD); Department of Infectious Diseases, Istituto Superiore di Sanità, Rome, Italy (M Menegon MSc, C Severini PhD); Department of Medical Microbiology and Infection Prevention, Amsterdam University Medical Centers, Amsterdam, the Netherlands (P Mens PhD, H D Schallig PhD); Department of Paediatrics, University of Calabar, Calabar, Nigeria (Prof M Meremikwu MD); Ifakara Health Institute, Ifakara, Tanzania (D Mosha PhD); Population Health & Immunity Division, Walter and Eliza Hall Institute, Melbourne, Australia (Prof I Mueller PhD); Makerere University-John Hopkins University collaboration, Kampala, Uganda (P Natureeba MD); Noguchi Memorial Institute for Medical Research, University of Ghana, Legon, Accra, Ghana (Prof N Ndam PhD); Institute for Tropical Medicine, University of Tübingen, Germany (Prof F Ntoumi PhD, Prof A A Adegnika PhD); Department of Medical Laboraory Science, Achievers University Owo, Nigeria (Prof O A Oduwole PhD); Malaria Branch, KEMRI/Centre for Global Health Research, Kisumu, Kenya (K Otieno MSc); Universidad del Valle de Guatemala, Guatemala City, Guatemala (N Padilla PhD); Department of Tropical Medicine, Bernhard Nocht Institute for Tropical Medicine & University Medical Center Hamburg-Eppendorf, Hamburg, Germany (Prof M Ramharter MD, R Z Manego, Prof G Mombo-Ngoma); Department of Immunology and Microbiology, University of Colorado Anschutz Medical Campus, Aurora, CO, USA (Prof R Rochford PhD, K R Sabourin PhD), University of Health and Allied Sciences, Ho, Ghana (Prof H Tagbor); Department of Tropical Medicine, Medical Microbiology & Pharmacology, John A. Burns School of Medicine University of Hawaii, Honolulu, Hawaii, USA (Prof D W Taylor PhD); Columbia University Irving Medical Center, New York, United States (B J Wylie MD); Faculté des sciences de la santé de Cotonou, Université d'Abomey-Calavi, Bénin (Prof D M Zannou MD); Institute for Global Health and Infectious Diseases, School of Medicine, University of North Carolina, Chapel Hill, NC, USA (Prof S R Meshnick PhD).

Table of Contents Supplement

Table of Contents Supplement ... 4

Supplemental Methods... 6

Supplement 1: Abbreviation list (alphabetical order) ... 6

Supplement 2: Supplemental information on search ... 6

Supplement 3: Requested variables for IPD analysis ... 7

Supplement 4: Supplemental information on quality assessment ... 7

Supplement 5: Supplemental information on variables and multivariable models ... 8

Supplement 6: Differences with protocol ... 11

Supplemental Results ... 12

Supplement 7: Molecular methods used, proportion of microscopy positive/NAAT-negative results and sensitivity of microscopy ... 12

Supplement 8: Additional information on fever and malaria ... 12

Supplement 9: Additional analyses ... 12

Supplemental Tables ... 15

Table S1: Characteristics of included studies, in order of continent and time period of study ... 15

Table S2: Description of malaria treatments, IPTp and study arms of trials in IPD studies (by continent and year of study) ... 19

Table S3: Risk of bias assessment of included IPD studies ... 22

Table S4: Risk of bias assessment of included aggregated data studies ... 24

Table S5: Characteristics of participants by timing of test and source of blood, IPD and aggregated data ... 25

Table S6: Availability of co-factors and characteristics of combined IPD set ... 26

Table S7: Pregnancy: Prevalence of malaria by microscopy compared to NAATs by study (IPD, order by region and country) ... 27

Table S8: Delivery: Prevalence of malaria by microscopy compared to NAATs by study (IPD, order by region and country) ... 29

Table S9: Median and interquartile range of microscopy positive/NAAT negative test results overall and by region ... 30

Table S10: Pregnancy: Pooled prevalence and odds ratio of fever by malaria test results ... 31

Table S11: Pooled prevalence of species as assessed by PCR among microscopic and submicroscopic infections by region, meta-analysis ... 32

Table S12: Malaria species as assessed by PCR as risk factor for submicroscopic malaria among PCR-positive infections, IPD* ... 33

Table S13: Multivariable analyses of factors associated with submicroscopic and microscopic infections by gravidity, base model ... 34

Table S14: Multivariable analyses of factors associated with submicroscopic and microscopic *Plasmodium falciparum* and *Plasmodium vivax* infection in Asia and America, IPD base model ... 37

Table S15: Multivariable analyses of factors associated with submicroscopic and microscopic malaria infections in pregnancy by region, first ANC visits only, IPD base model 38
Table S16: Multivariable models by region to assess the effect of inclusion of the variable "Gestational age" into the analyses of submicroscopic and microscopic malaria infections, IPD ... 40
Table S17: Multivariable models in Africa to assess the effect of inclusion of the variable "HIV infection" into the analyses of submicroscopic and microscopic malaria infections, IPD 42
Table S18: Multivariable models in Africa to assess the effect of inclusion of the variable "Antimalarial use" into the analyses of submicroscopic and microscopic malaria infections, IPD .. 44
Table S19: Delivery: Multivariable analyses of factors associated with submicroscopic and microscopic malaria infections peripheral blood, IPD: Base model and variables of interest with limited sample size 46
Table S20: Delivery: Multivariable analyses of factors associated with placental submicroscopic and microscopic malaria infections, IPD: Base model and variables of interest with limited sample size 48
Table S21: Microscopy and fever results at second scheduled study visit in pregnancy after submicroscopic malaria at enrolment 50
Table S22: Sensitivity analysis: Comparing IPD and aggregated data for study outcomes 50
Table S23: Sensitivity analysis: Effect of quality assessment factor in multivariable models 51
Table S24: Sensitivity analysis: Effect of quality of blood smear reading on risk factors for submicroscopic and microscopic malaria, multivariable models in pregnancy, IPD 53
Table S25: Sensitivity analysis: Multivariable analyses of factors associated with submicroscopic and microscopic malaria infections in pregnancy by region, using an alternative measure of transmission level*, IPD 55
Supplemental Figures 58
Figure S1: Map of included studies 58
Figure S2A: Proportion of submicroscopic malaria among NAAT-positive test results by malaria transmission level among first ANC attendees 59
Figure S2B: Proportion of submicroscopic malaria among NAAT-positive test results in pregnancy by malaria transmission level by gravidity 60
Figure S3: Probability of fever by malaria status, gestational age and region 61
Figure S4: Prevalence of submicroscopic and microscopic malaria in studies with regular follow up visit in pregnancy by gestational age 62
Figure S5: Proportion of submicroscopic malaria (forest plot), microscopic malaria (column) and no malaria (column) at a subsequent scheduled follow up visit in pregnancy among women with submicroscopic malaria at enrolment, by region 66
Figure S6: Probability and 95% confidence interval of microscopic and submicroscopic malaria by gestational age 67
Figure S7: Funnel plot for studies with information on submicroscopic malaria infection during pregnancy 68
References 69

Supplemental Methods

Supplement 1: Abbreviation list (alphabetical order)

ANC	Antenatal clinic
BS	Blood smear
CI	Confidence interval
HIV	Human immunodeficiency virus
IPD	Individual participant data
IPTp	Intermittent preventive treatment of malaria in pregnancy
IQR	Interquartile range
IRS	Indoor residual spraying
IST	Intermittent screening and treatment of malaria
ITN	Insecticide treated net
NAAT	Nucleic acid amplification test (PCR or LAMP)
NA	Not available or not applicable
NR	Not reported
PCR	Polymerase chain reaction
LAMP	Loop-mediated isothermal amplification
LLIN	Long-lasting insecticide treated nets
cRDT	Conventional Rapid diagnostic malaria test
SD	Standard deviation

Supplement 2: Supplemental information on search

The first mention of PCR for malaria identification in the Malaria in Pregnancy library was in 1998; we used 1997 to detect additional materials that might have been missed in the library using the terms "Polymerase chain reaction" OR PCR OR subpatent OR submicroscopic OR sub-patent OR sub-microscopic OR Lamp OR "loop-mediated isothermal amplification". The search was repeated in Pubmed (450 results), Google Scholar (446 results) and the Global Health database with “AND pregnan* AND malaria” added to the search terms. No new studies were identified relative to the search using the Malaria in Pregnancy Library database. We also searched reference lists and review articles identified in the primary search. We contacted researchers with relevant publications to identify any further, unpublished studies. Note that in some studies, PCR was used on samples that were preserved from older studies, with the inclusion of studies conducted before 1998 as a result. The last search was conducted on November 10, 2021. An update of the search using the Malaria in Pregnancy Library and PubMed from November 10, 2021, to 26 November 2022, resulted in 36 and 33 entries, respectively; after deduplication of the combined file, 48 entries from 40 studies remained and five new studies with potential information on submicroscopic malaria were identified. There was insufficient information in these five studies for data-extraction. These five studies were not included in the analyses described in this paper.

This study was registered in Prospero-CRD42015027342 as a systematic review and not as an individual participant data analysis. This will be corrected at a later stage.

Pubmed search: ("Polymerase chain reaction" OR PCR OR subpatent OR submicroscopic OR sub-patent OR sub-microscopic OR Lamp OR "loop-mediated isothermal amplification") AND pregnan* AND Malaria AND 1997/01/01:2021/10/01[dp]

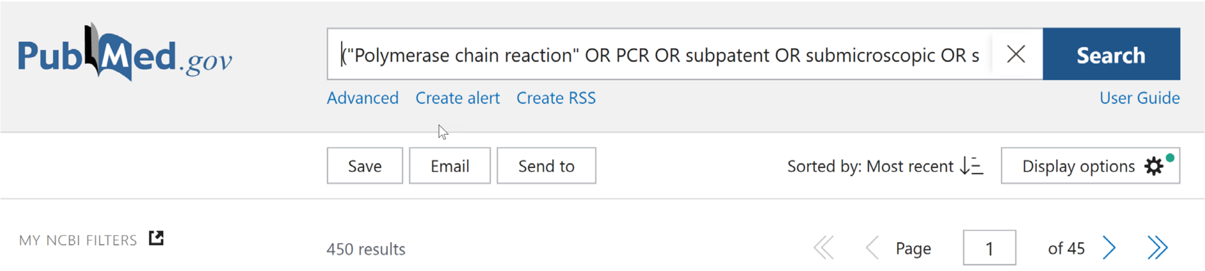

Supplement 3: Requested variables for IPD analysis

We emailed authors of eligible studies to inform about the study and inquire about their interest. Authors were emailed at least three times before deciding not to include the study as IPD, unless they informed us about their interest. When authors were not interested to take part, or could not be contacted, or for other reasons did not take part, an attempt was made to extract the data needed. The following variables were requested from IPD studies: Study identity number, date of visit, malaria test (microscopy, PCR, cRDT, LAMP), malaria test result, species and country where available, source of blood for malaria test at delivery (maternal or placental), gravidity, age, history of fever, body temperature, history of recent antimalarial treatment, gestational age, net use, ITN use, IPTp use or other type of malaria prevention, haemoglobin, and treatment arm if part of a trial. If at delivery: singleton, birth outcome (live or stillbirth), birthweight, newborn sex. Where available: HIV status, rural residence, IRS, smoker, iron and folate supplementation, maternal anthropometry and placental histology. These last four variables were not used in the current analyses. Each individual dataset was checked for ranges and consistency; information was compared with publications where possible, and variables were created or reformatted for merging. No irregularities were identified when checking the IPD data. For aggregated data, the study information was extracted independently by AMvE and AM using excel sheets and compared, and only used if both persons agreed on the extracted information.

Supplement 4: Supplemental information on quality assessment

In this individual patient data analysis, participating studies were surveys, trials and cohorts. Although some included studies were trials, these were not selected because of their research question but because of the availability of the exposure and outcomes of interest (submicroscopic malaria). For this reason, we used an adaptation of the Newcastle Ottawa Scale. We assessed the availability of primary exposures (malaria by test and compartment) and outcomes of interest (submicroscopic malaria prevalence, birthweight, gestational age, haemoglobin), and if the assessment of the outcomes was blind for the exposure, as far as could be assessed. Confounders were defined as factors that were associated with the exposure and the outcome but were not on the causal pathway. We assessed whether important known confounders were available such as age, gravidity, setting (rural or urban place of living) and HIV status. We recorded the availability of co-variates such as recent malaria treatment and malaria prevention (net use, ITN, IRS, IPTp). In the current study, submicroscopic malaria prevalence was our outcome of interest, and we used the criteria as described in Table S0. Studies could attain one point for each question, with a maximum of six points. We considered scores of 5 or 6 as indicators of high quality of the contributing data. Studies were not excluded from the analyses based on their quality score, but a sensitivity analysis was conducted to assess the impact of low-quality studies. The quality score information was included in the sensitivity analysis as a dichotomy (score of 5 or 6 vs. <5) or continuous score, to assess if results would differ by quality of the study (Table S23). We used the same criteria for studies where data was extracted from the articles when we were not able to retrieve the data set.

Table S0: Criteria for quality assessment

	Focus area	Category options
1	**Representativeness of the population**	**a)** Truly representative of women in the community (e.g. random selection in community) * **b)** Somewhat representative of the average woman in the community (e.g. ANC at enrolment) * **c)** Selected group of women (e.g. women who were enrolled from a maternity population or delivered in a maternity) **d)** No description of the derivation of the group
2	**Inclusion/exclusion criteria: exclusion of group that may affect outcome?**	**a)** No clear inclusion/exclusion criteria that may affect exposure or outcome* **b)** Inclusion/exclusion criteria may affect exposure (e.g. HIV-status, certain age groups, only live borns, only newborns with certain weights, certain gravidity groups) **c)** No description of criteria
3	**Ascertainment of exposure**	**a)** Microscopy, RDT, PCR or LAMP appropriately described and valid, reliable* If 50% or more of tests well described, 1 point **b)** Incomplete description or doubtful methods **c)** No description
4	**Availability of potential confounding variables (age, gravidity, HIV, setting)**	**a)** All mentioned potential confounders available* (if 3 or 4 out of 4 available) **b)** Some available, incomplete **c)** All not available
5	**Availability of co-variates (IPT, ITN, IRS, malaria treatment)**	**a)** Malaria treatment and prevention available* (1 point if treatment and prevention are available) **b)** Only partially available* **c)** Not available
6	**Attrition outcome**	**a)** Complete - all subjects accounted for* **b)** Outcome not available for all subjects but unlikely to introduce bias - small number lost - <20%, or description provided of those lost * If majority not available but random selection, still 1 point. **c)** Outcome for less than 80% of people with exposure data and no description of those lost **d)** No statement

Supplement 5: Supplemental information on variables and multivariable models

We added information on geospatial coordinates of the study sites (longitude and latitude obtained from Google Earth: https://www.google.com/earth/), and the prevalence of sulfadoxine-pyrimethamine (SP) resistance markers for studies in Africa only (Ala437Gly and Lys540Glu substitutions in the *dhps* gene), obtained from the publication, study authors, or existing prevalence maps of *P. falciparum dhps* mutations).[1,2]

The following covariates were included in the analyses as appropriate and available:

- Maternal covariates: Age, gravidity, HIV infection, use of malaria prevention (ITNs, IPTp, IRS) or antimalarials, gestational age or trimester in pregnancy or at delivery
- Location or study site co-variates: malaria season, location of living (rural vs. urban), indicator of malaria transmission intensity for study site at the time of study, prevalence of molecular markers of SP resistance in the study area around the time of the study (*Pfdhps*-A437G and *Pfdhps*-K540E), species, region
- Study level covariates: study design, risk of bias assessment, details of blood smear reading.

Random effects models were used to allow for heterogeneity in risk across studies. The I^2 was used as indicator of heterogeneity.

Studies in Asia and the Pacific were combined as “Asia”. An indicator of malaria transmission intensity was obtained from the Malaria Atlas Project (2020-layer, https://malariaatlas.org) for the mid-year of the study for meta-analyses and for the study year for the one-stage IPD analyses. The Malaria Atlas Project estimates reflect the average prevalence of *P. falciparum* infection among children 2-10 years of age to within five kilometres of any location. We defined low malaria transmission as a $Pf\mathrm{PR}_{2\text{-}10}$ <10%, moderate transmission as $\mathrm{PfPR}_{2\text{-}10}$ 10-34%, and high transmission as $Pf\mathrm{PR}_{2\text{-}10}$ ≥35%. Low transmission can be considered similar to hypo-endemic malaria.[3] Hyper-endemic and holo-endemic malaria are officially defined as a $Pf\mathrm{PR}_{2\text{-}10}$ of 50-75%, and >75%, respectively; however, malaria transmission has declined and in our participating studies only five were in high and one in a holo-endemic transmission area.[4] To obtain meaningful sample sizes, we reduced the cut-off for high transmission to $Pf\mathrm{PR}_{2\text{-}10}$ ≥35%.

Age was used in three groups, defined as <20 years, 20-29 years, and 30+ years, which was used as baseline. Gravidity was used in three groups (primigravidae, secundigravidae and gravidae 3+). A few studies had age data and gravidity only as categories available and not as a continuous variable, and for this reason, the total number available in categories is higher than for age or gravidity as continuous variables.

We checked collinearity between age and gravidity before including these variables in multivariate analysis. We evaluated collinearity using the variance inflation factor (VIF) and tolerance, defined as 1/VIF. A tolerance value lower than 0.1 is comparable to a VIF of 10 and may merit further investigation.[5] Results were as follows:

Analysis	Correlation coefficient, p-value	Variance inflation factor	Tolerance
During pregnancy	0.5908, p<0.0001	1.54	0.6509
Delivery, dataset for maternal blood	0.6137, p<0.0001	1.60	0.6233
Delivery, dataset for placental blood	0.6049, p<0.0001	1.58	0.6341

To indicate net use, we defined “any net” to indicate a net of any type that was used as defined by the study, e.g. in the previous night, during pregnancy, or available in the household, and ITN use as reported by the participant. Several trials and cohorts provided ITNs at the time of enrolment in pregnancy; if this was reported, we considered at the time of delivery that these participants used ITNs. This information was not included in the variable “any net use” at the time of delivery. There was wide variety in report of antimalarials for time period, number of times and type of antimalarial. Additionally, trials used several different treatments as IPTp or IST. We combined reported antimalarial use and IPTp in one variable, indicating a history of antimalarial use in pregnancy. For malaria prevalence during pregnancy, gestational age was included in multivariable models as a continuous variable, whereas at the time of delivery we included the variable “preterm”, to indicate if the gestational age was before 37 weeks of gestation at the time of delivery. Rainy season was defined according to the dataset where this was available or using the date variable and information from the source article or author where available, or from the internet for the location of the study when first-mentioned sources were not available. For studies where no visit date was available, we assigned the midyear of the study as study year. To assess the effect of quality of blood smear reading, variables were created for each study indicating the number of high power fields examined before declaring a slide negative (100 high power fields versus 200 or more), and a variable

indicating number of persons reading the slides (1 person and 10% quality control versus 2 persons and a third person in case of disagreements).

Prevalence. Estimates for prevalence by study were generated within the merged dataset for the IPD analyses. A dataset was created with prevalence by study, and this was merged with the dataset from individual studies from the extracted data for the two-stage analysis. The Freeman-Tukey double arcsine transformation was used for metaprop.[6] The variance stabilizing transformation of the proportions as proposed by Freeman and Tukey normalizing the outcomes of proportions before pooling,[6] is defined as;

$$\sin^{-1}\sqrt{\frac{r_i}{n_i+1}} + \sin^{-1}\sqrt{\frac{r_i+1}{n_i+1}}$$

The asymptotic variance of the transformed variable is defined as $\frac{1}{n_i+0.5}$.[7]

This transformation is intended to achieve approximate normality. This method has the advantage that the variances of the arcsine-based transformations depend only on the sample sizes, which are typically fixed, known values.[8] A continuity correction of 0.5 was used for cells with 0-values in the meta-analysis of odds ratios.[9]

Because of the high heterogeneity, the pooled summary estimate of the prevalence of submicroscopic and microscopic malaria was reported in combination with the median and range, allowing the readers their own judgment, and providing a range that may cover the likely result. Individual participant data analysis was conducted to try to address some of the heterogeneity through the covariate adjustment for dataset where the individual participant data was available.

Fever. For the random-effect multivariable logit models of fever (xtlogit), the main interest was the relationship between malaria infection and fever, and the modifying effect of transmission level or gravidity (as potential indicators of background immunity) on this relationship. Adjustment was made for base model covariates and gestational age. Although we explored the effects of history of antimalarial use, ITN or any type of net use, HIV infection, and IRS, we did not use these variables in the models because of the drop in sample size; however, the most important results are described in supplement 8. We only presented the relationship between malaria and fever in pregnancy because at delivery, multiple additional causes of fever can be present due to the delivery process.

Species. Malaria species was not examined for placental blood because of the differences in ability to sequester in the placenta by species.

Factors associated with microscopic and submicroscopic malaria. We explored factors which are known to affect exposure to infected mosquito bites (incidence of infection: ITN use and net use, IRS, rainy season, level of malaria transmission, continent, rural vs. urban setting), factors associated with clearance of parasites in the blood (antimalarial treatment) and factors known to affect immunological response to malaria (age, gravidity, gestational age and HIV-status). Multiple imputations could not be conducted for missing information, as these variables were not missing at random but, in many cases, reflected the study design/protocol. The last group of factors may affect chronicity of the infection, once an infection has been acquired. Multivariable models were made for Africa and America/Asia separately. In areas of moderate and high malaria transmission, a gravidity specific pattern of malaria is well known, with primigravidae having the highest prevalence of malaria, and a decrease in prevalence with increasing pregnancy number which may be due to the development of gravidity specific immunity to malaria.[10,11] In most high transmission regions the prevalence does not differ much by gravidity among gravida 3+. However, in areas of low

transmission, such a pattern is not seen, with primi- and multigravidae having a similar prevalence of malaria. Because of these patterns, an interaction term between gravidity and transmission level was explored in models for Africa. The model improved with the interaction term, and for this reason in Africa analyses were stratified by by high (*Pf*PR$_{2-10}$ ≥35%) and moderate-to-low transmission areas (*Pf*PR$_{2-10}$ <35%); Gravidity was divided into primigravidae [G1], secundigravidae [G2], and multigravidae [G3+], except for analyses with non-convergence, when categories could be combined. . There were no known HIV-infected participants in America/Asia, so this covariate was not evaluated in this region. In some models at delivery, study design could not be included because of collinearity.

Outcome of submicroscopic malaria at enrolment in the consecutive scheduled visit. Nine studies had information on submicroscopic malaria at follow up visits. All studies had scheduled study visits every 4-6 weeks. We explored the outcome of submicroscopic malaria at enrolment at the subsequent scheduled study visit. Note that submicroscopic malaria at enrolment is generally detected too late to have clinical consequences in the form of treatment; however, in trials women generally will receive treatment at enrolment, and in cohort studies in Africa, IPTp with SP may have started. To avoid labelling inadvertent unscheduled as scheduled study visits, a minimum interval of 14 days was used between enrolment and consecutive scheduled study follow up visit. No maximum limit was set to allow the largest sample size available. We calculated prevalence of submicroscopic, microscopic and no malaria infection at the 2nd scheduled study visit when submicroscopic malaria was present at enrolment and calculated the time interval in days. We explored several definitions for time interval between visits, with or without upper or lower limits and these models gave similar results. Co-variates explored were base model variables, interval between study visits in days, markers of SP resistance and antimalarial treatment at enrolment, and all were initially included in the multivariable model. All locations in moderate-to-low transmission areas were in high transmission areas, and for this reason, a combination variable of SP resistance and transmission level was created, indicating low-moderate transmission-high SP resistance, high transmission-low SP resistance, and high transmission-high SP resistance. Factors with a p-value >=0.1 (Wald test) in both parts of the model (for submicroscopic and microscopic malaria) in the multivariate model were removed. Additionally, removed factors were one by one tested (and added) if they improved the subsequent multivariable model with the p-value <0.1 (in at least one part of the model).

Supplement 6: Differences with protocol

The protocol is available at https://www.wwarn.org/tools-resources/subpatent-malaria-study-group-protocol. In the main multivariable analyses, variables were not removed based on their p-values, because this would not allow comparison of the effect of variables across time points, level of malaria transmission or region. Because of the variability in availability of co-factors, the choice was made for a base model and additional factors with limited sample size. This study was restricted to microscopic and submicroscopic malaria; RDTs, placental histology and NAAT-test characteristics were not evaluated in the current article. To allow assessment of the different categories at the same time (microscopic, and submicroscopic malaria versus no infection), a multinomial model was used instead of xtlogit. Because of the high observed heterogeneity, prevalence results were also summarised as study median and range.

Supplemental Results

Supplement 7: Molecular methods used, proportion of microscopy positive/NAAT-negative results and sensitivity of microscopy

Of the 68 participating studies, quantititative real time PCR was used in 30 studies (44.1%), nested PCR in 31 studies (45.6%) and 4 studies used LAMP (5.9%). For the three remaining studies, the molecular method was unavailable for one study; one used both methods, and one used LDRFMA ("polymerase chain reaction /ligase detection reaction-fluorescent microsphere assay", Stanisic et al. 2015).[12] The 18S rRNA gene was most commonly (67·6%) targeted in studies using PCR.

The proportion of microscopy positive/PCR negative test results ranged from 0-5.9% in pregnancy (n=54, mean 1.0%, sd 1.3, median 0.6, IQR 0-1.6%; median of 0 in 11 subgroups in the Americas, 1.3%, IQR 0.1-2.0% in 11 subgroups in Asia, and 1.0%, IQR 0.2-2.0% in 32 subgroups in Africa; Table S9). The pooled sensitivity of microscopy to detect NAAT-positive infections was 36.7% (30.8-42.9, I^2=97%) during pregnancy, 27.4% (22.6-32.5, I^2=91%) for maternal and 26.7% (21.4-32.4, I^2=90%) for placental blood at delivery.

Supplement 8: Additional information on fever and malaria

Gravidity was not associated with fever in the overall model and in America/Asia, but the odds of fever were higher among primigravidae in the model for Africa overall (aOR=1.58, 1.12-2.23, p=0.0095 compared to gravidae 3+), and in high malaria transmission areas (aOR=2.29, 1.08-2.62, p=0.0222), but not in moderate-to-low transmission areas (aOR 1.42, 0.81-2.50, p=0.22). Only in the model for Africa overall was an interaction noted between transmission level and gravidity. An age <20 years was associated with fever in moderate-to-low transmission areas in Africa as a protective factor (aOR=0.38, 0.18-0.78, p=0.0092). Of the variables available for a limited sample size (HIV, antimalarial use for treatment or prevention, ITN or bednet use), antimalarial use was associated with fever in the overall model (aOR=2.02, 1.48-2.77, p<0.0001, N=8094), and ITN use was a protective factor (aOR 0.69, 0.51-0.94, p=0.0166, N=7967). Results for malaria tests at delivery were similar to pregnancy (data not shown). The probability of being febrile decreased with the progression of pregnancy but this trend was not malaria specific as it was evident among women with microscopic infections, submicroscopic infections and uninfected women (Figure S3); gestational age was associated with fever in the overall model (aOR 0.98, 0.97-0.99, p<0.0001), but the interaction terms between gestational age and malaria were not significant (p=0.29 and p=0.43 for interaction term of gestational age with submicroscopic and microscopic malaria, respectively).

Supplement 9: Additional analyses

Evaluation of predictors of infection that were restricted to data collected at the first ANC visit only, reduced the sample size to about one third (Africa) and a quarter (Americas/Asia) of the full sample. There was no data from 1st ANC visits among multigravidae in high transmission areas or the Americas (Table S15). Except for the model in moderate transmission areas, it was not possible to include *Pf*PR_{2-10} . Compared to the full model, less factors were associated with malaria infections, but that may have been due to the difference in sample size. In both transmission areas in Africa, young women (<20 years compared to 30+ years) and primigravidae (compared to gravidae 3+) were at risk for microscopic malaria, and young age remained a risk factor for submicroscropic malaria. In Asia, microscopic infections were more prevalent among women < 20 years (aOR 2.60, 95% CI 1.37-4.93) compared to women aged 30+ years.

Submicroscopic malaria was present throughout pregnancy and this was consistent by region, transmission intensity and gravidity (Figure S6). Only in primi- and secundigravidae in high

transmission areas in Africa was the proportion of submicroscopic malaria less than microscopic malaria; for multigravidae and all gravidities in other transmission levels, submicroscopic infections were more common than microscopic among NAAT-positive infections (Figure S6). Among studies with follow-up visits, the longest documented period of submicroscopic malaria was 161 days over five consecutive visits for a woman in Benin.

In Africa, compared to using 200+ high power fields before declaring a negative smear, using 100 power fields was associated with a decreased detection of malaria, whereas the use of 1 reader and 10% quality control compared to 2+ readers was associated with an increase in the detection of microscopic but not submicroscopic malaria (no malaria as reference group, Table S25). In this African model, rainy season changed from a significant into a non-significant factor for microscopic malaria; the association between other significant factors (age, gravidity and transmission level) strengthened or stayed the same. In the Americas and Asia, no effect of quality of slide reading was detected on the detection of malaria, and the association between malaria and other factors (age, gravidity, transmission) remained similar (Table S25).

In sensitivity analyses evaluating the quality of blood smear reading, only in moderate-to-low transmission areas in Africa an effect was seen, with definition of a negative slide as “no parasites in 100 high power fields” resulting in lower detection of microscopic malaria (aOR 0.32, 95%CI 0.19-0.55) compared to a definition of “no parasites in 200+ high power fields” (Table S25). Having 1 reader and 10% quality control of slides resulted in a higher prevalence of microscopic malaria (aOR of 4.31, 2.21-8.41) compared to 2+ readers. Indicators of the quality of blood smear reading were not predictive in high transmission areas in Africa or in the Americas/Asia.

Using a different indicator of transmission level (malaria infection by PCR in the first trimester among all gravidae) resulted in different sample size by transmission region in Africa. However, overall the same variables remained important as predictors of submicroscopic and microscopic malaria (Table S25). In Africa in moderate-to-low transmission areas, the aOR of $PfPR_{2-10}$ for submicroscopic malaria was 0.97, 0.94-0.99 (indicating a 3% decrease in submicroscopic malaria with 1% increase in transmission level); the corresponding aOR for the alternative transmission indicator was 1.12, 1.04-1.21 (indicating a 12% increase in submicroscopic malaria with a 1% increasing transmission level, which seemed high). In the Americas/Asia, the aOR of $\textit{Pf}PR_{2-10}$ for microscopic malaria was 1.00, 0.96-1.04 (p=0.98); the corresponding aOR for the alternative transmission level indicator for microscopic malaria was 1.04, 1.01-1.07 (p=0.0130) (Table S25).

A funnel plot for included studies for submicroscopic malaria infection in pregnancy can be seen in Figure S7; The graph looks reasonably symmetrical, suggesting no clear publication bias. The p-value for the Egger test was 0.43, suggesting there was no indication of a small study effect.

Information on existing versus newly diagnosed HIV infection as part of antenatal screening was not available to differentiate between women with known HIV infection on antiretroviral therapy and prophylaxis with daily cotrimoxazole (which has antimalarial properties)[13] with known HIV infection versus women with newly diagnosed HIV infections not yet on antiretrovirals and cotrimoxazole. It was encouraging to see that treatment with dihydroartemisinin-piperaquine was associated with protection from submicroscopic infections at the subsequent visit among the limited sample of women from whom information was available at enrolment and follow-up. This indicates that effective malaria treatment reduces subsequent risks for submicroscopic malaria.

References

1. Malaria Atlas Project. Maps. 2023. https://data.malariaatlas.org/maps?layers=Malaria:202206_Global_Pf_Parasite_Rate (accessed April 05, 2023).
2. World Wide Antimalarial Resistance Network (WWARN). Molecular Surveyor. 2023. http://www.wwarn.org/dhfr-dhps-surveyor/#0 (accessed January 23, 2023).
3. Hay SI, Smith DL, Snow RW. Measuring malaria endemicity from intense to interrupted transmission. *Lancet Infect Dis* 2008; **8**(6): 369-78.
4. Weiss DJ, Lucas TCD, Nguyen M, et al. Mapping the global prevalence, incidence, and mortality of Plasmodium falciparum, 2000-17: a spatial and temporal modelling study. *Lancet* 2019; **394**(10195): 322-31.
5. O'Brien MO. A caution regarding rules of thumb for variance inflation factors. *Quality & Quantity* 2007; **41**: 673-90.
6. Nyaga VN, Arbyn M, Aerts M. Metaprop: a Stata command to perform meta-analysis of binomial data. *Arch Public Health* 2014; **72**(1): 39.
7. Freeman MF, Tukey JW. Transformations related to the angular and the square root. *Ann Math Stat* 1950; **21**(4): 607-11.
8. Lin L, Xu C. Arcsine-based transformations for meta-analysis of proportions: Pros, cons, and alternatives. *Health Sci Rep* 2020; **3**(3): e178.
9. Ren Y, Lin L, Lian Q, Zou H, Chu H. Real-world performance of meta-analysis methods for double-zero-event studies with dichotomous outcomes using the cochrane database of systematic reviews. *J Gen Intern Med* 2019; **34**(6): 960-8.
10. Mayor A, Bardaji A, Macete E, et al. Changing trends in *P. falciparum* burden, immunity, and disease in pregnancy. *N Engl J Med* 2015; **373**(17): 1607-17.
11. Desai M, ter Kuile FO, Nosten F, et al. Epidemiology and burden of malaria in pregnancy. *Lancet Infect Dis* 2007; **7**(2): 93-104.
12. Stanisic DI, Moore KA, Baiwog F, et al. Risk factors for malaria and adverse birth outcomes in a prospective cohort of pregnant women resident in a high malaria transmission area of Papua New Guinea. *Trans R Soc Trop Med Hyg* 2015; **109**(5): 313-24.
13. Klement E, Pitche P, Kendjo E, et al. Effectiveness of co-trimoxazole to prevent *Plasmodium falciparum* malaria in HIV-positive pregnant women in sub-Saharan Africa: an open-label, randomized controlled trial. *Clin Infect Dis* 2014; **58**(5): 651-9.

Supplemental Tables

Table S1: Characteristics of included studies, in order of continent and time period of study

Study	Study period	Country	Study design	Timing available	Overall Sample size	Number with information on malaria			Malaria species PCR	Mean age (sd) N	Proportion primigravidae	Proportion HIV-infected	Proportion living rural	ITN (%)*
						Pregnancy	Delivery (maternal)	Delivery (placenta)						
**Americas, individual participant data**														
Bardaji 2017[1]	2009-2011	Brazil, Colombia, Guatemala	Cohort	Enrolment, delivery	5709	899	887	37	Yes	23.5 (6.2) 897	284/898 (31.6)	NA	NA	408/897 (45.5) †
Elbadry 2017[2]	2014-2015	Haiti	Survey	Enrolment	313	304	NA	NA	No	27.5 (7.1) 295	27/194 (13.9)	NA	313/313 (100.0)	193/296 (65.2)
Gavina 2018[3]	2014-2017	Colombia	Cohort	Enrolment, follow up, delivery	186	184	179	178	Yes	23.3 (6.6) 185	61/186 (32.8)	NA	NA	90/170 (52.9) †
Vasquez 2018[4]	2016-2016	Colombia	Survey	Enrolment	435	435	NA	NA	Yes	23.8 (6.0) 435	143/435 (32.9)	NA	195/418 (46.7)	316/431 (73.3) †
Vasquez 2018[4]	2016-2017	Colombia	Survey	Delivery	329	NA	329	329	Yes	24.7 (5.1) 329	149/329 (45.3)	0/329 (0.0)	160/326 (49.1)	283/327 (86.5) †
Vasquez 2020[5]	2017-2017	Colombia	Survey	Enrolment	861	861	NA	NA	Yes	24.8 (5.5) 861	265/861 (30.8)	NA	183/861 (21.3)	NA
**Americas, aggregated data**														
Parekh 2010[6]	2004-2004	Peru	Survey	Delivery	193	NA	166	174		Median, IQR: 22, 14-44, n=189	NR	NR	NR	NR
Arango 2013[7]	2005-2011	Colombia	Survey	Delivery	96	NA	95	95		22.8 (6.4) 96	31/90 (34.4)	NR	NR	NR
Agudelo 2013[8]	2008-2011	Colombia	Survey	Delivery	129	NA	121	121		21.9 (6.7) 121	NR	48 tested, 0 positive	NR	0
**Asia, individual participant data**														
Stanisic 2015[9]	2005-2008	PNG	Cohort	Enrolment and delivery	350	350	309	232	Yes	24.9 (5.4) 337	132/350 (37.7)	NA	339/347 (97.7)	20/318 (6.3)
Singh 2015[10]	2007-2008	India	Survey	Enrolment	2477	2477	NA	NA	Yes	23.7 (3.6) 2477	1131/2477 (45.7)	0/682 (0.0)	1013/2477 (40.9)	7/2477 (0.3)
Singh 2015[10]	2007-2008	India	Survey	Delivery	948	NA	NA	948	Yes	24.3 (3.9) 948	471/948 (49.7)	0/266 (0.0)	353/948 (37.2)	3/948 (0.3)
Bardaji 2017[1]	2009-2011	India, PNG	Cohort	Enrolment, delivery	3679	551	579	193	Yes	24.1 (4.6) 547	262/551 (47.5)	NA	NA	123/456 (27.0) †
Unger 2015[11]	2010-2013	PNG	Trial	Enrolment, follow up, delivery	2242	2219	2005	1344	Yes	24.6 (5.5) 2242	1097/2238 (49.0)	NA	1393/2123 (65.6)	1701/2234 (76.1) †
Pava 2016[12]	2013-2013	Indonesia	Survey	Enrolment	45	44	NA	NA	Yes	28.6 (7.0) 45	NA	NA	NA	16/45 (35.6) †
Ahmed 2019[13]	2013-2016	Indonesia	Trial	Enrolment, delivery	2279	2071	1853	1763	Yes	26.8 (6.1) 2219	632/2279 (27.7)	0/2279 (0.0)	1713/2279 (75.2)	865/2279 (38.0) †
**Asia, aggregated data**														
Ahmed 2014[14] Abstract	2008-2009	Indonesia	Survey	Pregnancy	4230	2598	NA	NA		NR	NR	NR	NR	NR
Ahmed 2015[15]	2012-2012	Indonesia	Survey	Delivery	950	NA	934	NA		28.8 (6.2) 950	17.8 (169/950)	NR	100% rural	265/950 (27.9)
**Africa, individual participant data**														

Study	Study period	Country	Study design	Timing available	Overall Sample size	Number with information on malaria			Malaria species PCR	Mean age (sd) N	Proportion primigravidae	Proportion HIV-infected	Proportion living rural	ITN (%)*
						Pregnancy	Delivery (maternal)	Delivery (placenta)						
Walker-Abbey 2005[16]	1995-1998	Cameroon	Survey	Delivery	303	NA	302	198	Yes	25.7 (5.8) 297	72/296 (24.3)	NA	0/303 (0.0)	NA
Mockenhaupt 2000[17]	1998-1998	Ghana	Survey	Enrolment	528	528	NA	NA	Yes	26.6 (6.3) 521	129/528 (24.4)	NA	194/528 (36.7)	NA
Mockenhaupt 2002[18]	2000-2000	Ghana	Survey	Delivery	893	NA	889	887	Yes	26.3 (6.3) 875	318/882 (36.1)	NA	466/891 (52.3)	NA
Malhotra 2009[19]	2000-2003	Kenya	Survey	Delivery	706	NA	629	583	Only for Pf	25.8 (6.2) 517	192/702 (27.4)	NA	706/706 (100.0)	NA
Leke 2010[20]	2001-2005	Cameroon	Cohort	Enrolment, follow up, delivery	120	56	48	NA	Only for Pf	25.0 (6.7) 119	26/120 (21.7)	NA	120/120 (100.0)	0/59
Adegnika 2006[21]	2003-2004	Gabon	Survey	Delivery	145	NA	130	81	Only for Pf	24.4 (6.5) 145	35/145 (24.1)	NA	NA	95/145 (65.5) †
Menendez 2008[22]	2003-2005	Mozambique	Trial	Delivery	1030	NA	605	443	Only for Pf	24.2 (6.6) 1030	266/1030 (25.8)	208/870 (23.9)	1030/1030 (100.0)	NA
Luntamo 2012[23]	2003-2006	Malawi	Trial	Delivery	1190	NA	453	NA	Only for Pf	25.0 (6.4) 1190	267/1190 (22.4)	144/1075 (13.4)	1190/1190 (100.0)	877/1190 (73.7)
Landis 2009[24]	2005-2006	DRC	Cohort	Delivery	164	NA	124	NA	Yes, only Pf detected	27.5 (5.3) 164	43/164 (26.2)	4/164 (2.4)	0/164 (0.0)	NA
Dobano 2018[25]	2005-2007	Mozambique	Survey	Delivery	240	NA	238	NA	Only for Pf	NA	60/217 (27.6)	0/240 (0.0)	NA	26/240 (10.8)
Cottrell 2015[26]	2008-2010	Benin	Cohort	Enrolment and delivery	997	993	651	544	Only for Pf	26.3 (6.1) 984	185/997 (18.6)	14/851 (1.6)	552/997 (55.4)	NA
Oduwole 2011[27]	2009-2009	Nigeria	Survey	Delivery	204	NA	202	203	Only Pf	27.5 (4.8) 204	68/204 (33.3)	NA	NA	40/204 (19.6)
Cohee 2014[28]	2009-2010	Malawi	Cohort	Enrolment and delivery	447	438	289	305	Only for Pf	20.1 (3.2) 447	282/447 (63.1)	0/447 (0.0)	NA	224/445 (50.3) †
Natureeba 2014[29]	2009-2013	Uganda	Trial	Delivery	389	NA	NA	301	No	29.3 (5.4) 388	24/389 (6.2)	389/389 (100.0)	NA	147/387 (38.0)
Patel 2016[30]	2010-2011	Malawi	Survey	Delivery	1142	NA	1103	1118	No	24.2 (5.5) 1142	349/1142 (30.6)	0/1141 (0.0)	980/1142 (85.8)	761/1142 (66.6)
Kattenberg 2012[31]	2010-2011	Burkina Faso	Survey	Enrolment	418	380	NA	NA	Only for Pf	25.6 (5.1) 61	NA	NA	NA	NA
Gonzalez 2014[32]	2010-2012	Benin, Gabon, Mozambique	Trial	Delivery	946	NA	943	938	Only for Pf	24.3 (6.3) 946	292/946 (30.9)	0/946 (0.0)	NA	NA
Gonzalez 2014 HIV+[33]	2010-2012	Kenya, Mozambique	Trial	Delivery	768	NA	765	764	Only for Pf	26.7 (5.7) 768	94/768 (12.2)	768/768 (100.0)	NA	NA
Denoeud-Ndam 2013[34]	2010-2012	Benin	Trial	Enrolment and delivery	417	394	291	300	Only for Pf	29.2 (4.7) 416	47/417 (11.3)	417/417 (100.0)	0/417 (0.0)	262/407 (64.4) †
Williams 2016[35]	2010-2012	Burkina Faso, The Gambia, Mali	Trial	Enrolment	4048	1861	NA	NA	Yes	20.0 (3.1) 4048	2199/4026 (54.6)	0/4048 (0.0)	NA	2610/3968 (65.8)

Study	Study period	Country	Study design	Timing available	Overall Sample size	Number with information on malaria			Malaria species PCR	Mean age (sd) N	Proportion primigravidae	Proportion HIV-infected	Proportion living rural	ITN (%)*
						Pregnancy	Delivery (maternal)	Delivery (placenta)						
Williams 2016 Ghana[35]	2010-2012	Ghana	Trial	Enrolment, follow up, delivery	1306	1258	897	NA	Yes	21.6 (3.7) 1306	727/1303 (55.8)	0/1306 (0.0)	NA	489/1305 (37.5)
Mahamar 2021[36]	2010-2015	Mali	Cohort	Enrolment, follow up, delivery	1885	1851	1808	1776	No	24.1 (6.2) 1885	444/1885 (23.6)	NA	NA	960/1878 (51.1)
Arinaitwe 2013[37]	2011-2011	Uganda	Survey	Delivery	565	NA	NA	565	No	24.6 (6.1) 565	183/565 (32.4)	0/565 (0.0)	565/565 (100.0)	437/565 (77.4)
Daud 2014[38]	2011-2012	Kenya	Cohort	Enrolment, follow up, delivery	111	111	108	NA	Only for Pf	22.4 (6.0) 111	NA	16/111 (14.4)	NA	NA
Madanitsa 2016[39]	2011-2013	Malawi	Trial	Enrolment, follow up, delivery	1844	1788	1541	1490	Only for Pf	22.5 (5.1) 1843	627/1844 (34.0)	0/1844 (0.0)	1829/1833 (99.8)	350/1843 (19.0)
Nkhoma 2017[40]	2011-2013	Malawi	Trial	Enrolment	1341	1341	NA	NA	Only for Pf	24.7 (6.1) 1324	297/1338 (22.2)	178/1286 (13.8)	NA	NA
Mosha 2014[41]	2012-2012	Tanzania	Survey	Delivery	349	NA	NA	348	Only for Pf	25.2 (6.9) 349	130/349 (37.2)	12/342 (3.5)	NA	330/349 (94.6)
Ntoumi 2016[42] (Francine 2016)	2012-2013	Congo B	Survey	Enrolment	363	363	NA	NA	Only for Pf	24.7 (6.4) 363	95/355 (26.8)	NA	NA	NA
Desai 2015[43]	2012-2014	Kenya	Trial	Enrolment, follow up, delivery	1543	1429	1267	1231	Only for Pf	23.4 (5.8) 1543	514/1543 (33.3)	0/1543 (0.0)	NA	1102/1543 (71.4) †
Chaponda 2015[44]	2012-2014	Zambia	Cohort	Enrolment	1085	1074	NA	NA	Only for Pf	25.6 (6.3) 1085	261/1085 (24.1)	143/1084 (13.2)	1085/1085 (100.0)	534/1085 (49.2) †
Braun 2015[45]	2013-2013	Uganda	Survey	Delivery	765	NA	743	NA	Yes	25.5 (5.7) 765	231/762 (30.3)	92/765 (12.0)	NA	583/709 (82.2) †
Arnaldo 2018[46]	2014-2015	Mozambique	Survey	Delivery	918	NA	918	NA	Only Pf	23.1 (6.2) 918	398/918 (43.4)	0/918 (0.0)	NA	847/918 (92.3)
Kakuru 2016[47]	2014-2015	Uganda	Trial	Enrolment and delivery	300	299	288	280	No	21.6 (4.0) 300	111/300 (37.0)	1/300 (0.3)	NA	261/300 (87.0)
Mbouamboua 2019[48]	2014-2015	Congo B	Survey	Delivery	371	NA	370	370	Only for Pf	25.9 (6.4) 371	81/371 (21.8)	NA	NA	240/371 (64.7)
Accrombessi 2018[49]	2014-2016	Benin	Cohort	Enrolment, follow up, delivery	393	367	233	221	Only for Pf	26.7 (5.1) 393	32/393 (8.1)	6/341 (1.8)	NA	373/380 (98.2) †
Briggs 2019[50]	2016-2017	Uganda	Trial	Enrolment, follow up, delivery	782	782	NA	640	Only for Pf	23.8 (5.7) 782	195/782 (24.9)	0/782 (0.0)	NA	111/782 (14.2)
**Africa, aggregated data**														
Singer 2004[51]	2001-2001	Burkina Faso	Survey	Delivery	1316	NA	463	460		NR	NR	NR	NR	NR
Perrault 2009[52]	2002-2008	Kenya	Survey	Delivery	157	NA	157	157		30.1% < 20 years	43.9 (69/157)	24.8 (39/157)	65/157 rural	NR
Adam 2005[53]	2003-2004	Sudan	Survey	Pregnancy	142	142	NA	NA		25.9 (5.4) 124	35.2 (44/125)	NR	NR	NR
Bouyou-Akotet 2010[54]	2005-2006	Gabon	Survey	Delivery	378	NA	185	NA		23 (5) 185	80.5 (149/185)	NR	NR	NR
Kapito-Tembo 2010 Thesis[55]	2005-2009	Malawi	Survey	Pregnancy	1128	1101	NA	NA		Median 27	6.2 (70/1121)	100%	Rural 100%	653/1092 (59.8)

Study	Study period	Country	Study design	Timing available	Overall Sample size	Number with information on malaria			Malaria species PCR	Mean age (sd) N	Proportion primigravidae	Proportion HIV-infected	Proportion living rural	ITN (%)*
						Pregnancy	Delivery (maternal)	Delivery (placenta)						
Newman 2009[56] HIV+	2008-2009	Uganda	Survey	Delivery	150	NA	NA	150		Median, IQR: 23, 19-29, n=161	19.3 (31/161)	100	NR	NR
Newman 2009[56] HIV-	2008-2009	Uganda	Survey	Delivery	336	NA	NA	336		Median, IQR: 28, 23-33, n=356	37.6 (134/356)	0	NR	NR
Nwaefuna 2015[57]	2009-2009	Ghana	Cohort	Pregnancy	872	870	NA	NA		32.9 (5.0) 872	29.4 (257/872)	NR	NR	NR
Elbashir 2011[58]	2010-2010	Sudan	Survey	Delivery	107	NA	NA	107		26.4 (6.9) 107	28.0 (30/107)	NR	NR	NR
Kyabayinze 2016[59]	2010-2012	Burkina Faso	Cohort	Delivery	555	NA	554	NA		Median, IQR: 24, 20-29, n=650	26.2 (170/650)	0	Periurban	NR
Kashif 2013[60]	2011-2011	Sudan	Survey	Delivery	150	NA	NA	150		26.0 (6.0) 150	Parity 1.6 (1.7) 150	NR	NR	NR
Kyabayinze 2016[59]	2011-2012	Uganda	Cohort	Delivery	297	NA	294	NA		Median, IQR: 23, 20-27, n=340	33.8 (115/340)	0	NR	NR
Matangila 2014[61]	2012-2012	DRC	Survey	Pregnancy	166	166	NA	NA		Median, IQR: 27, 22-33, n=166	26.0 (86/332)	3.9% in Kinshasa, no individual results	Semi-rural	143/332 (43.1)
Lamptey 2018[62]	2013-2014	Ghana	Cohort	Pregnancy	126	126	NA	NA		Median, IQR: 24, 20-28, n=125	NR	NR	NR	98/125 (78.4) †
Fadlelseed 2017[63]	2014-2014	Sudan	Survey	Delivery	153	NA	153	153		25.7 (6.5) 153	45.8 (70/153)	NR	Rural 75.2 (115/153)	56/153 (36.6) †
Ruh 2018[64]	2014-2014	DRC	Survey	Delivery	250	NA	250	NA		27.2 (6.5) 250	40.0 (100/250)	NR	NR	NR
Natureeba 2017[65]	2014-2015	Uganda	Trial	Delivery	200	NA	197	197		29.3 (5.4) 389	6.2 (24/389)	100	NR	147/389 (37.8)
Quakyi 2019[66]	2015-2017	Ghana	Survey	Pregnancy	987	987	NA	NA		27.3 (5.9) 987	25.2 (249/987)	0	Urban and periurban	421/987 (42.7) †
Quakyi 2019[66]	2015-2017	Ghana	Survey	Delivery	935	NA	935	935		27.7 (5.5) 935	21.0 (196/935)	0	Urban and periurban	627/935 (67.1) †
Samuels 2019[67] Abstract	2018-2018	Kenya	Survey	Pregnancy	483	483	NA	NA		NR	NR	NR	NR	NR
Tadesse 2020[68]	2018-2019	Ethiopia	Cohort	Pregnancy	149	149	NA	NA		NR	NR	NR	NR	26/149 (17.4)

NA: Not available. NR, not reported.
Only for Pf: NAAT only for Pf detection. Only Pf: NAAT species detection not specified, but only Pf infections reported
*ITN use reported at enrolment in pregnancy for surveys or cohorts in pregnancy, or at enrolment for surveys at delivery; use during pregnancy or use in the last night
† Any net, not clear if ITN or not

Table S2: Description of malaria treatments, IPTp and study arms of trials in IPD studies (by continent and year of study)

Study	Country	Study period	Study design	Timing of prevalence subpatency available	Sample size	Treatment arms	Reported IPT in pregnancy or antimalarial use other than SP in pregnancy or at delivery (%) if available	Reported IPTp at delivery (%) any dose	Reported IPTp at delivery (%), 2+ doses	Reference used for SP-resistance markers (Ala437Gly and Lys540Glu)
**Americas, individual participant data**										
Bardaji 2017[1]	Brazil, Colombia, Guatemala	2009-2011	Cohort	Enrolment and delivery	9388	NA	Antimalarial use: Enrolment: 24/8429 (0.3) Delivery: 90/3816 (2.4) Drugs not specified	No IPTp policy		NA
Elbadry 2017[2]	Haiti	2014-2015	Survey pregnancy	Enrolment	313	NA	Antimalarial use 49/246 (19.9), drugs not specified	No IPTp policy		NA
Gavina 2018[3]	Colombia	2014-2017	Cohort	Enrolment and delivery	186	NA	Antimalarial use 111/117 (94.9), drugs not specified	No IPTp policy		NA
Vasquez 2018[4]	Colombia	2016-2016	Survey	Pregnancy	435	NA	Antimalarial use 13/408 (3.2) drugs not specified	No IPTp policy		NA
Vasquez 2018[4]	Colombia	2016-2017	Survey	Delivery	329	NA	Antimalarial use 12/316 (3.8) drugs not specified	No IPTp policy		NA
Vasquez 2020[5]	Colombia	2017-2017	Survey	Pregnancy	861	NA	Antimalarial use 0/861 (0.0) in past 48 hours	No IPTp policy		NA
**Asia, individual participant data**										
Stanisic 2015[9]	PNG	2005-2008	Cohort	Enrolment and delivery	350	NA	Antimalarial use 35/347 (10.1) enrolment 320/340 (94.1) delivery, drugs not specified	No IPTp policy		NA
Singh 2015[10]	India	2007-2008	Survey	Pregnancy	2477	NA	Antimalarial use 3/101 (3.0) CQ (2) PQ (1)	No IPTp policy		NA
Singh 2015[10]	India	2007-2008	Survey	Delivery	948	NA	Antimalarial use 2/948 (0.2)	No IPTp policy		NA
Bardaji 2017[1]	India, Papua New Guinea	2009-2011	Cohort	Enrolment and delivery	9388	NA	Antimalarial use Enrolment: 24/8429 (0.3) Delivery: 90/3816 (2.4)	No IPTp policy		NA
Unger 2015[11]	PNG	2010-2013	Trial	Enrolment and delivery	2242	SP CQ once IPT SP AZ monthly	Antimalarial use 246/2187 (11.3) enrolment	2236/2242 (99.7)	1071/2242 (47.8)	NA
Pava 2016[12]	Indonesia	2013-2013	Survey	Pregnancy	45	NA	NR	No IPTp policy		NA
Ahmed 2019[13]	Indonesia	2013-2016	Trial	Enrolment and delivery	2279	IPT DP 3+ IST DP 3+ ST 1x, DP	NR		591/681 in DP3+ arm (86.8) got IPT-DP 2+	NA
**Africa, individual participant data**										
Walker-Abbey 2005[16]	Cameroon	1995-1998	Survey	Delivery	303	NA	NR	No IPTp policy		NA
Mockenhaupt 2000[17]	Ghana	1998-1998	Survey	Pregnancy	528	NA	Antimalarial use 341/528 (64.6) CQ 73.3%, CQ+PYR 17.6%, PYR 9.1%	No IPTp policy		NA
Mockenhaupt 2002[18]	Ghana	2000-2000	Survey	Delivery	893	NA	Antimalarial use 783/869 (90.1)	No IPTp policy		NA

Study	Country	Study period	Study design	Timing of prevalence subpatency available	Sample size	Treatment arms	Reported IPT in pregnancy or antimalarial use other than SP in pregnancy or at delivery (%) if available	Reported IPTp at delivery (%) any dose	Reported IPTp at delivery (%), 2+ doses	Reference used for SP-resistance markers (Ala437Gly and Lys540Glu)
							CQ 13.8%, CQ+PYR 49.6%, PYR 36.7%			
Malhotra 2009[19]	Kenya	2000-2003	Survey	Delivery	706	NA	Antimalarial use 78/358 (21.8); with information AQ 12, Q 1, SP 17	No IPTp policy		NA
Leke 2010[20]	Cameroon	2001-2005	Cohort	Pregnancy and delivery	120	NA	Antimalarial use 26/61 (42.6) enrolment, during pregnancy (CQ) 53/91 (58.2), delivery CQ 71.2%, Q 5.8%, 13 NR	No IPT policy		Chauvin 2015[69]
Adegnika 2006[21]	Gabon	2003-2004	Survey	Delivery	145	NA	Antimalarial use 143/145 (98.6), CQ (92.3); CQ with AQ (2.8%); CQ with Q (4.9%)	0/145 (0.0)		No IPTp adopted at time of study
Menendez 2008[22]	Mozambique	2003-2005	Trial	Delivery	1042	IPT SP 2<br>IPT placebo 2 (100% ITN coverage in both arms)		520/1042 (49.9)	520/1042 (49.9)	Enosse 2008[70]
Luntamo 2013[23]	Malawi	2003-2006	Trial	Delivery	1190	IPT SP 2<br>IPT SP monthly<br>IPT SP monthly & 2 doses AZ		1190/1190 (100)	1179/1190 (99.1)	Gutman 2015[71]
Landis 2009[24]	DRC	2005-2006	Cohort	Delivery	164	NA		163/164 (99.4)	149/164 (90.9)	Mita 2011[72]
Dobano 2018[25]	Mozambique	2005-2007	Survey delivery	Delivery	240	NA		NA		Enosse 2008[70]
Cottrell 2015[26]	Benin	2008-2010	Cohort	Enrolment and delivery	997	NA		944/997 (94.7)	853/997 (85.6)	Moussilliou 2013[73]
Oduwole 2011[27]	Nigeria	2009-2009	Survey	Delivery	204	NA	Antimalarial use 70/204 (34.3) ACT 91%, CQ 9%	114/204 (55.9)		Esu 2018[74]
Cohee 2014[28]	Malawi	2009-2010	Cohort	Enrolment and delivery	447	NA	Antimalarial use at enrolment 61/445 (13.7)	426/447 (95.3)	382/447 (85.5)	Artimovich 2015[75]
Natureeba 2014[29]	Uganda	2009-2013	Trial	Delivery	389	EFV<br>LPV-r	377/377 (100) CTX	0/377 (0.0)		Tumwebaze 2017[76]
Kattenberg 2012[31]	Burkina Faso	2010-2011	Survey	Pregnancy	418	NA		NA		Tahita 2015[77]
Patel 2016[30]	Malawi	2010-2011	Survey	Delivery	1142	NA	155/1142 (13.6) ACT 78.7%, CTX 9.7%, Q 5.8%, combinations 5.8%	1118/1141 (98.0)	959/1141 (84.0)	Artimovich 2015[75]
Denoeud-Ndam 2013[34]	Benin	2010-2012	Trial	Enrolment and delivery	417	CTX daily<br>CTX daily & IPT MQ 2+<br>IPT MQ 2+	Antimalarial use at enrolment: 44/410 (10.7)<br>At delivery: 417/417 (100)	208/417 (49.9)		Moussilliou 2013[73]
Gonzalez 2014[32]	Benin, Gabon, Mozambique	2010-2012	Trial	Delivery	946	IPT SP 2<br>IPT MQ 2		946/946 (100)	946/946 (100)	Benin: Moussilliou 2013[73]<br>Gabon: Guerra 2017[78]<br>Mozambique: Gupta 2018[79]

Study	Country	Study period	Study design	Timing of prevalence subpatency available	Sample size	Treatment arms	Reported IPT in pregnancy or antimalarial use other than SP in pregnancy or at delivery (%) if available	Reported IPTp at delivery (%) any dose	Reported IPTp at delivery (%), 2+ doses	Reference used for SP-resistance markers (Ala437Gly and Lys540Glu)
Gonzalez 2014[33] HIV+	Kenya, Mozambique	2010-2012	Trial	Delivery	768	IPT MQ 3 & daily CTX<br>IPT placebo 3 & daily CTX	768/768 (100) CTX	380/768 (49.5)	3 doses: 380/380 (100)	Kenya: Lucchi 2015[80]<br>Mozambique: Gupta 2018[79]
Williams 2016[35]	Burkina Faso, The Gambia, Mali	2010-2012	Trial	Enrolment	4048	IPT SP 2<br>IST SP 2<br>IST AQ-AS 2		2025/4048 (50.0)	1842/2025 (91.0)	Burkina Faso: Tahita 2015[77]<br>The Gambia: Ndiaye 2013[81]<br>Mali: Desai 2016[82]
Williams 2016[35] Ghana	Ghana	2010-2012	Trial	Enrolment and delivery	1306			653/1306 (50.0)	579/1306 (44.3)	Tahita 2015[77]
Mahamar 2021[36]	Mali	2010-2015	Cohort	Enrolment and delivery	1885	NA	820/1884 (43.5)<br>64 Q, 756 ACT	1786/1884 (94.8%)	1358/1884 (72.1%)	Diawara 2017[83]
Arinaitwe 2013[37]	Uganda	2011-2011	Survey	Delivery	565	NA		533/565 (94.3)	330/565 (58.4%)	Desai 2016[82]
Daud 2014[38]	Kenya	2011-2012	Cohort	Enrolment and delivery	111	NA				Lucchi 2015[80]
Madanitsa 2016[39]	Malawi	2011-2013	Trial	Enrolment and delivery	1844	IPT SP 3+<br>IST DP 3+		921/1844 (50.0)	886/1844 (48.0)	Artimovich 2015[75]
Nkhoma 2017[40]	Malawi	2011-2013	Trial	Enrolment	1341	NA	NR	NA		Gutman 2015[71]
Mosha 2014[41]	Tanzania	2012-2012	Survey	Delivery	349	NA	68/349 (19.5)	318/349 (91.1)	181/349 (51.9)	Baraka 2015[84]
Ntoumi 2016[42] (Francine 2016)	Congo B	2012-2013	Survey	Pregnancy	363	NA	135/363 (37.2%) IPT-SP before enrolment<br>SP 2+: 76/363 (20.9%)	NA		Nkoli Mandoko 2018[85]
Desai 2015[43]	Kenya	2012-2014	Trial	Enrolment and delivery	1543	IPT SP 3+<br>IPT DP 3+<br>IST DP 3+		1029/1543 (66.7)	942/1543 (61.0)	Lucchi 2015[80]
Braun 2015[45]	Uganda	2013-2013	Survey	Delivery	765	NA	168/720 (23.3) drug unknown	605/725 (83.5)	No info on SP2+	Baraka 2017[86]
Chaponda 2015[44]	Zambia	2012-2014	Cohort	Enrolment	1085	NA		722/728 (99.2)	598/728 (82.1)	Desai 2016[82]
Kakuru 2016[47]	Uganda	2014-2015	Trial	Enrolment and delivery	300	IPT SP 3<br>IPT DP 3<br>IPT DP monthly		300/300 (100)	291/300 (97)	Conrad 2017[87]
Arnaldo 2018[46]	Mozambique	2014-2015	Survey	Delivery	918	NA		855/918 (93.1)	No info on SP2+	Gupta 2018[79]
Mbouamboua 2019[48]	Congo B	2014-2015	Survey	Delivery	371	NA		311/371 (83.8)		Nkoli Mandoko 2018[85]
Accrombessi 2018[49]	Benin	2014-2016	Cohort	Enrolment and delivery	393	NA		251/260 (96.5)	199/260 (76.5)	Huijben 2020[88]
Briggs 2019[50]	Uganda	2016-2017	Trial	Enrolment	782	IPT DP 3+<br>IPT SP 3+		731/782 (93.5)	709/782 (90.7)	Conrad 2017[87]

AZ, azithromycin. ACT, artemisinin-based combination therapy. AQ, amodiaquine. AS, artesunate. CQ, chloroquine. CTX, cotrimoxazole. DP, dihydroartemisinin–piperaquine. EFV, efavirenz-based antiretroviral therapy. LPV-r, lopinavir/ritonavir-based antiretroviral therapy. NA, not applicable or available. IPT, intermittent preventive treatment. IPTp, intermittent preventive treatment in pregnancy. IST, intermittent screening and treatment. MQ, mefloquine. PYR, pyrimethamine. PQ, primaquine. SP, sulfadoxine-pyrimethamine. ST: screen and treat.

Table S3: Risk of bias assessment of included IPD studies

Study	Country/ countries	Location(s)	Design	Timing	Location of enrolment	Repre-sentative	Inclusion criteria	Availability potential confounders	Availability co-variates	Ascertainment malaria test	Attrition malaria test	Total	Final
Accrombessi 2018[49]	Benin	Soava and Akassato districts	Cohort	Both	Community	1	1	1	0	1	0	4	Moderate-to-low
Adegnika 2006[21]	Gabon	Lambarene	Survey	Delivery	Maternity	0	1	0	1	1	1	4	Moderate-to-low
Ahmed 2019[13]	Indonesia	Sumba/Timika	Trial	Both	ANC	1	0	1	1	1	1	5	High
Arinaitwe 2013[37]	Uganda	Tororo	Survey	Delivery	Maternity	0	0	1	1	0	1	3	Moderate-to-low
Arnaldo 2018[46]	Mozambique	Chokwe district	Survey	Delivery	Maternity	0	0	1	0	1	1	3	Moderate-to-low
Bardaji 2017[1]	India, Guatamala, Papua New Guinea, Brazil, Colombia	Bikaner, Fray Bartolome de las Casas, Madang, Manaus, Tieralta	Cohort	Pregnancy	ANC	1	1	0	1	1	0	4	Moderate-to-low
Braun 2015[45]	Uganda	Fort Portal	Survey	Delivery	Maternity	0	1	1	1	1	1	5	High
Briggs 2019[50]	Uganda	Busia	Trial	Both	ANC	1	0	1	1	1	1	5	High
Chico 2017[89]	Zambia	Nchelenge (two health centers)	Cohort	Pregnancy	ANC	1	0	1	0	1	1	4	Moderate-to-low
Cohee 2014[28]	Malawi	Blantyre	Cohort	Both	ANC	1	0	1	1	1	0	4	Moderate-to-low
Cottrell 2015[26]	Benin	Come district Akodeha, Come Central, Ouedeme-Pedah	Cohort	Both	ANC	1	1	1	0	1	0	4	Moderate-to-low
Daud 2104[38]	Kenya	Chulaimbo	Cohort	Both	ANC	1	0	1	0	0	1	3	Moderate-to-low
Denoeud-Ndam 2013[34]	Benin	Cotonou/Porto novo	Trial	Both	ANC	1	0	1	1	1	0	4	Moderate-to-low
Desai 2015[43]	Kenya	Bondo/Lwak/Madiany/Siaya	Trial	Both	ANC	1	0	1	1	1	1	5	High
Dobano 2018[25]	Mozambique	Manhica	Survey	Delivery	ANC	1	0	1	0	1	1	4	Moderate-to-low
Elbadry 2017[2]	Haiti	6 districts	Survey	Pregnancy	Community	1	0	1	1	1	1	5	High
Mahamar 2021[36]	Mali	Ouelessebougou	Cohort	Both	ANC	1	0	1	1	0	1	4	Moderate-to-low
Gavina 2018[3]	Colombia	Puerto Libertador	Cohort	Both	ANC	1	1	0	1	1	1	5	High
Gonzalez 2014[32] HIV-	Benin	Allada, Sekou, Attogon	Trial	Delivery	ANC	1	0	1	1	1	1	5	High
Gonzalez 2014[33] HIV+	Mozambique	Manhica, Maragra	Trial	Delivery	ANC	1	0	1	1	1	1	5	High
Kakuru 2016[47]	Uganda	Tororo	Trial	Both	ANC	1	0	1	1	1	1	5	High
Kattenberg 2012[31]	Burkina Faso	Nanoro	Survey	Pregnancy	ANC	1	1	0	0	1	0	3	Moderate-to-low
Landis 2009[24]	DRC	Kinshasa (Binza Maternity hospital)	Cohort	Both	ANC	1	1	1	0	1	0	4	Moderate-to-low
Leke 2010[20]	Cameroon	Ngali II	Cohort	Both	ANC	1	1	1	1	1	0	5	High
Luntamo 2012[23]	Malawi	Mangochi district (Lungwena HC)	Trial	Delivery	ANC	1	1	1	0	1	0	4	Moderate-to-low
Madanitsa 2016[39]	Malawi	Chikwawa/Madziabango/Mpemba	Trial	Both	ANC	1	0	1	1	1	1	5	High
Malhotra 2009[19]	Kenya	Kwale	Survey	Delivery	Maternity	0	0	0	0	1	1	2	Moderate-to-low
Mbouamboua 2019[48]	Congo B	Madibou	Survey	Delivery	Maternity	0	0	0	0	1	1	2	Moderate-to-low
Menendez 2008[22]	Mozambique	Manhica district	Trial	Both	ANC	1	1	1	0	1	0	4	Moderate-to-low
Mockenhaupt 2000[17]	Ghana	Agogo	Survey	Pregnancy	ANC	1	1	0	0	1	1	4	Moderate-to-low
Mockenhaupt 2002[18]	Ghana	Agogo	Survey	Delivery	Maternity	0	1	0	0	1	1	3	Moderate-to-low

Study	Country/ countries	Location(s)	Design	Timing	Location of enrolment	Repre-sentative	Inclusion criteria	Availability potential confounders	Availability co-variates	Ascertainment malaria test	Attrition malaria test	Total	Final
Mosha 2014[41]	Tanzania	Moshi/Rufiji	Survey	Delivery	Maternity	0	1	1	1	1	1	5	High
Natureeba 2014[29]	Uganda	Tororo	Trial	Delivery	ANC	1	0	1	1	1	0	4	Moderate-to-low
Nkhoma 2017[40]	Malawi	4 facilities in Mangochi district	Trial	Both	ANC	1	1	1	1	1	1	6	High
Ntoumi 2016[42] (Francine 2016)	Congo B	Madibou	Survey	Pregnancy	ANC	1	0	0	1	1	1	4	Moderate-to-low
Oduwole 2011[27]	Nigeria	Calabar	Survey	Delivery	Maternity	0	1	0	1	0	1	3	Moderate-to-low
Patel 2016[30]	Malawi	Blantyre, Madziabango, Mpemba	Survey	Delivery	Maternity	0	0	1	1	1	1	4	Moderate-to-low
Pava 2016[12]	Indonesia	Timika	Survey	Pregnancy	Community	1	1	0	1	1	1	5	High
Singh 2015[10]	India	Bastar district, Rajnandgaon district	Survey	Pregnancy	ANC	1	1	1	1	1	1	6	High
Singh 2015[10]	India	Bastar district, Rajnandgaon district	Survey	Delivery	Maternity	0	1	1	1	1	1	5	High
Stanisic 2015[9]	PNG	Alexishafen	Cohort	Both	ANC	1	0	1	1	0	1	4	Moderate-to-low
Unger 2015[11]	PNG	Alexishafen, Madang, Mugil, Yagaum	Trial	Both	ANC	1	0	1	1	1	1	5	High
Vasquez 2018[4]	Colombia	Apartado/Turbo/El Bagre	Survey	Pregnancy	ANC	1	0	1	1	1	1	5	High
Vasquez 2018[4]	Colombia	Apartado/Turbo/El Bagre	Maternity	Delivery	Maternity	0	0	1	1	1	1	4	Moderate-to-low
Vasquez 2020[5]	Colombia	Quibdo/Tumaco	Survey	Pregnancy	ANC	1	0	1	0	1	1	4	Moderate-to-low
Walker-Abbey 2005[16]	Cameroon	Yaounde (Central Hospital and Biyem Assi Hospital)	Survey	Delivery	Maternity	0	1	0	0	1	1	3	Moderate-to-low
Williams 2016[35]	Mali, Burkina Faso, Gambia, Ghana	Bamako/Basse/Kita/San Ziniare/Navrongo	Trial	Pregnancy	ANC	1	0	1	1	1	0	4	Moderate-to-low

Table S4: Risk of bias assessment of included aggregated data studies

Study	Country	Location(s)	Design	Timing	Location of enrolment	Repre-sentative	Inclusion criteria	Availability potential confounders	Availability co-variates	Ascertainment malaria tests	Attrition malaria tests	Total	Final
Adam 2005[53]	Sudan	New Halfa	Survey	Pregnancy	ANC	1	1	0	0	0	1	3	Moderate-to-low
Agudelo 2013[8]	Colombia	Turbo, Puerto Libertador	Survey	Delivery	Maternity	0	1	0	0	1	1	3	Moderate-to-low
Ahmed 2014[14]	Indonesia	Jayapura and South-West Sumba	Survey	Pregnancy	ANC	1	0	0	0	1	1	3	Moderate-to-low
Ahmed 2014[14]	Indonesia	Jayapura and South-West Sumba	Survey	Delivery	Maternity	0	0	0	0	1	1	2	Moderate-to-low
Ahmed 2015[15]	Indonesia	Sumba, southwest	Survey	Pregnancy	ANC	1	1	1	1	1	1	6	High
Arango 2013[7]	Colombia	Turbo, Puerto Libertador	Survey	Delivery	Maternity	0	1	0	1	1	1	4	Moderate-to-low
Bouyou-Akotet 2010[54]	Gabon	Libreville	Survey	Delivery	Maternity	0	0	0	0	1	1	2	Moderate-to-low
Elbashir 2011[58]	Sudan	Medani	Survey	Delivery	Maternity	0	1	0	0	1	1	3	Moderate-to-low
Fadlelseed 2017[63]	Sudan	New Halfa	Survey	Delivery	Maternity	0	0	1	1	1	1	4	Moderate-to-low
Kapito-Tembo 2010[55]	Malawi	Thyolo	Survey	Pregnancy	ANC	1	0	1	1	1	1	5	High
Kashif 2013[60]	Sudan	Gadarif	Survey	Delivery	Maternity	0	1	0	0	1	1	3	Moderate-to-low
Kyabayinze 2016[59]	Burkina Faso	Colsama	Cohort	Delivery	ANC	1	1	1	1	1	1	6	High
Kyabayinze 2016[59]	Uganda	Tororo	Cohort	Delivery	ANC	1	1	1	1	1	0	5	Moderate-to-low
Lamptey 2018[62]	Ghana	Asutsuare	Cohort	Pregnancy	ANC	1	0	0	0	0	0	1	Moderate-to-low
Matangila 2014[61]	DRC	Kinshasa	Survey	Pregnancy	ANC	1	0	0	1	1	0	3	Moderate-to-low
Natureeba 2017[65]	Uganda	Tororo	Trial	Pregnancy	ANC	1	0	1	1	1	0	4	Moderate-to-low
Newman 2009[56] HIV+	Uganda	Tororo	Survey	Delivery	Maternity	0	0	1	0	1	1	3	Moderate-to-low
Newman 2009[56] HIV-	Uganda	Tororo	Survey	Delivery	Maternity	0	0	1	0	1	1	3	Moderate-to-low
Nwaefuna 2015[57]	Ghana	Central Ghana	Survey	Pregnancy	ANC	1	1	0	0	1	1	4	Moderate-to-low
Quakyi 2019[66]	Ghana	Maamobi, Kpone on-Sea	Survey	Pregnancy	ANC	1	0	1	1	1	1	5	High
Quakyi 2019[66]	Ghana	Maamobi, Kpone on-Sea	Survey	Delivery	Maternity	0	0	1	1	1	1	4	Moderate-to-low
Parekh 2010[6]	Peru	Iquitos	Survey	Delivery	Maternity	0	1	0	0	1	1	3	Moderate-to-low
Perrault 2009[52]	Kenya	Kisumu, Siaya	Survey	Delivery	Maternity	0	1	1	0	0	1	3	Moderate-to-low
Ruh 2018[64]	DRC	Bandundu	Survey	Delivery	Maternity	0	0	0	0	1	1	2	Moderate-to-low
Samuels 2019[67]	Kenya	Siaya	Survey	Pregnancy	ANC	1	0	0	0	0	1	2	Moderate-to-low
Singer 2004[51]	Burkina Faso	Koupela district	Survey	Delivery	Maternity	0	1	0	0	1	0	2	Moderate-to-low
Tadesse 2020[68]	Ethiopia	Kafa zone	Cohort	Pregnancy	ANC	1	0	1	1	0	1	4	Moderate-to-low

Table S5: Characteristics of participants by timing of test and source of blood, IPD and aggregated data

Characteristic	Individual patient data: Participants (%) / studies / substudies*			Aggregated data: Participants (%) / studies / substudies*		
	Microscopy and NAATs in pregnancy	Microscopy and NAATs at delivery, maternal blood	Microscopy and NAATs at delivery, placental blood	Microscopy and NAATs in pregnancy	Microscopy and NAATs at delivery, maternal blood	Microscopy and NAATs at delivery, placental blood
**Overall**	25,401/ 28/ 56	21,820/ 32/ 58	18,451/ 29/ 57	6622/ 9 /10	4504/ 12/ 14	3035/ 12/ 13
Study design (%)						
Cohort	6841 (25.6)/ 10/ 16	5189 (23.5)/ 10/ 16	3461 (17.8)/ 7/13	275 (4.2)/ 2/ 2	848 (18.8)/ 1/ 2	0/ 0/ 0
Survey	5383 (21.6)/ 8/ 13	5805 (26.7)/ 11/ 15	5609 (30.8)/ 11/ 17	6347 (95.8)/ 7/ 8	3459 (76.8)/ 10/ 11	2838 (93.5)/ 11/ 12
Trial	13,177 (52.8)/ 10/ 27	10,826 (49.8)/ 11/ 27	9381 (51.5)/ 11/27	0 / 0/ 0	197 (4.4)/ 1/ 1	197 (6.5)/ 1/ 1
Region (%)‡						
West/Central Africa	7952 (30.1)/ 10/ 17	6491 (29.5)/ 13 / 17	5143 (27.0)/ 10/ 14	2149 (32.5)/ 4/ 5	2387 (53.0)/ 5/ 6	1395 (46.0)/ 2/ 3
East Africa	7144 (28.6)/ 8/ 16	9227 (42.5)/ 13 / 25	8307 (45.6)/ 12/ 25	1875 (28.3)/ 4/ 4	801 (17.8)/ 4/ 4	1250 (41.2) / 7/ 7
Americas	2683 (10.8)/ 5/ 11	1395 (6.4)/ 3 / 7†	544 (3.0)/ 3/ 7†	0 /0 / 0	382 (8.5)/ 3/ 3	390 (12.9)/ 3/ 3
Asia	7622 (30.6)/ 6/12	4707 (21.7)/ 4 / 9	4457 (24.4)/ 5/ 11	2598 (39.2)/ 1/ 1	934 (20.7)/ 1/ 1	0/ 0/ 0
Pf$PR_{2\text{-}10}$ category (%) ‡						
<10%	9933 (39.8)/ 11/ 24	5833 (26.9)/ 8/ 17	4955 (27.2)/ 10/ 20	2889 (43.6)/ 3/ 3	1654 (36.7)/ 6/ 6	800 (26.4)/ 6/ 6
10-34%	8678 (34.8)/ 12/ 23	10,584 (48.7)/ 19/ 32	7973 (43.7)/ 15/ 28	3250 (49.1)/ 5/ 6	1382 (30.7)/ 3/ 4	1132 (37.3)/ 2/ 3
≥35%	6790 (25.4)/ 8/ 9	5403 (24.4)/ 9/ 9	5523 (29.1)/ 9/ 9	483 (7.3)/ 1/ 1	1468 (32.6)/ 3/ 4	1103 (36.3)/ 4/ 4
Study period (median, range)	2011 (1998-2017)	2011 (1995-2017)	2011 (1995-2017)	2013 (2004-2018)	2011 (2001-2016)	2009 (2001-2016)

*Substudies: different locations within study, or groups if stratified enrolment
†One study with locations both in Americas and Asia
‡Studies could be conducted in different regions or areas with different malaria endemicity

Table S6: Availability of co-factors and characteristics of combined IPD set

Characteristic	Microscopy and NAATs during pregnancy*		Microscopy and NAATs at delivery, maternal blood*		Microscopy and NAATs at delivery, placental blood*	
	**Availability† (as % of overall)**	**Prevalence (%) or median (range)**	**Availability† (as % of overall)**	**Prevalence (%) or median (range)**	**Availability† (as % of overall)**	**Prevalence (%) or median (range)**
**Outcome available overall**	25,401 (28 studies)		21,820 (32 studies)		18,451 (29 studies)	
Rainy season	99.8 (1 | 44 ; 0 | 0)	15,151/25,357 (59.8)	95.7 (3 | 774; 8 | 174)	12,071/20,872 (57.8)	96.5 (2 | 548; 10 | 90)	10,684/17,813 (60.0)
Age (years) median (range) n	91.5 (0 | 0; 10 | 2165)	23 (11-55) 23,236	97.8 (1 | 237; 9 | 250)	24 (10-49) 21,333	95.4 (0 | 0; 10 | 851)	24 (10-49) 17,600
Age <20 years	98.2 (0 | 0; 9 | 467)	5880/24,934 (23.6)	98.9 (0 | 0; 9 | 250)	4937/21,570 (22.9)	98.8 (0 | 0; 9 | 220)	3785/18,231 (20.8)
Gravidity, median (range)	89.3 (5 | 2687; 6 | 27)	2 (1-15) 22,687	86.8 (3 | 2832; 8 | 52)	2 (1-14) 18,936	90.3 (1 | 1775; 4 | 20)	2 (1-13) 16,656
Primigravidae, n/N (%)	97.4 (3 | 532; 7 | 141)	8686/24,728 (35.1)	99.3 (1 | 108; 8 | 52)	7092/21,660 (32.7)	99.9 (0 | 0; 4 | 20)	5658/18,214 (31.1)
HIV-infected	56.7 (13 | 8987; 4 | 2029)	739/14394 (5.1)	61.0 (12 | 8287; 5 | 222)	1409/13,311 (10.6)	62.7 (11 | 6058; 6 | 820)	1547/11,573 (13.4)
Rural Setting	52.2 (15 | 12,005; 4 | 143)	8505/13,253 (64.2)	50.0 (17 | 10,788; 4 | 121)	8074/10,911 (74.0)	57.1 (15 | 7830; 4 | 94)	7551/10,527 (71.7)
Fever, history or documented‡	71.6 (6 | 6893; 10 | 310)	1262/18,198 (6.9)	64.0 (14 | 7510; 12 | 346)	726/13,964 (5.2)	64.5 (12 | 6359; 11 | 198)	498/11,894 (4.2)
Any net use	81.4 (7 | 4537; 13 | 197)	10,179/20,667 (49.3)	47.6 (17 | 11,335; 6 | 105)	7796/10,380 (75.1)	51.2 (14 | 8947; 6 | 49)	6557/9455 (69.4)
ITN use likely§	45.1 (19 | 12,993; 6 | 954)	4041/11,454 (35.3)	53.9 (15 | 9636; 4 | 416)	11,244/11,768 (95.6)	62.4 (11 | 6116; 6 | 814)	10,580/11,521 (91.8)
Antimalarial use in pregnancy¶	33.0 (13 | 14,320; 8 | 2710)	1054/8371 (12.6)	93.0 (4 | 822; 6 | 703)	15,794/20,295 (77.8)	95.9 (2 | 374; 5 | 385)	13,680/17,693 (77.3)
IRS	44.2 (19 | 13,628; 6 | 556)	1597/11,169 (14.3)	6.0 (30 | 20,504; 0 | 0)	111/959 (11.6)	14.2 (26 | 15,828; 1 | 1)	279/2254 (12.4)
Gestational age (weeks)	94.0 (1 | 44; 17 | 1487)	21 (1-46) 23,870	83.9 (5 | 2025; 21 | 1488)	39 (6-46) 18,307	88.3 (2 | 781; 23 | 1384)	39 (10-46), 16,286
Trimester	94.0 (1 | 44; 17 | 1487)					
First (1-16 weeks)		5718/23,871 (24.0)	NA	NA	NA	NA
Second (17-27 weeks)		14,532/23,871 (60.9)				
Third (≥28 weeks)		3,621/23,871 (15.2)				
Preterm delivery (< 37 weeks)		NA	88.3 (4 | 1161; 18 | 1397)	2480/19,262 (12.9)	88.8 (2 | 781; 18 | 1288)	2113/16,382 (12.9)
First antenatal visit	99.8 (1 | 44; 0 | 0)	7656/25,357 (30.2)	NA	NA	NA	NA

Abbreviations: HIV, human immunodeficiency virus. IPD, individual participant data. NAATs, nucleic acid amplification tests (PCR or LAMP). ITN, insecticide treated net. IRS, indoor residual spraying.
*Participants with microscopy positive/PCR negative test results excluded
† In brackets (number of studies where variable is missing because data was not collected or available | number of participants involved; number of studies with some participants missing this information | number of participants for which this variable is missing)
‡A history of fever in the past week or documented fever as defined by the study
§Either reported ITN use at enrolment or delivery or received ITN at the start of a cohort or trial in pregnancy (delivery only)
¶At delivery, 43% of women with a maternal blood result and 49% of women with a placental blood result had received 2 or more doses of an antimalarial

Table S7: Pregnancy: Prevalence of malaria by microscopy compared to NAATs by study (IPD, order by region and country)

Study	Country	Location	Mid-year study	*Pf*PR$_{2-10}$*		Microscopy vs. NAAT			
					Sample size	BS-/ NAAT+ (%)	BS+/ NAAT+ (%)	BS+/ NAAT- (%)	BS-/ NAAT- (%)
**Americas**									
Bardaji 2017[1]	Brazil	Manaus	2010	0.0	299	27 (9.0)	3 (1.0)	0 (0.0)	269 (90.0)
Vasquez 2018[4]†	Colombia	Tumaco	2016	0.0	120	3 (2.5)	4 (3.3)	0 (0.0)	113 (94.2)
Vasquez 2018[4]†	Colombia	Apartado/Turbo/El Bagre-P	2017	0.0	209	1 (0.5)	1 (0.5)	0 (0.0)	207 (99.0)
Gavina 2018[3]	Colombia	Puerto Libertador	2015	0.0	184	11 (6.0)	4 (2.2)	0 (0.0)	169 (91.8)
Bardaji 2017[1]	Colombia	Tieralta	2010	0.0	300	21 (7.0)	5 (1.7)	0 (0.0)	274 (91.3)
Vasquez 2020[5]†	Colombia	Tumaco	2017	0.0	469	9 (1.9)	15 (3.2)	0 (0.0)	445 (94.9)
Vasquez 2020[5]†	Colombia	Quibdo	2017	0.5	392	11 (2.8)	13 (3.3)	0 (0.0)	368 (93.9)
Vasquez 2018[4]	Colombia	Quibdo	2016	1.2	106	0 (0.0)	5 (4.7)	0 (0.0)	101 (95.3)
Bardaji 2017[1]	Guatemala	Fray Bartolome de las Casas	2010	0.0	300	63 (21.0)	0 (0.0)	0 (0.0)	237 (79.0)
Elbadry 2017[2]	Haiti	South Haiti	2014	0.0	94	33 (35.1)	4 (4.3)	0 (0.0)	57 (60.6)
Elbadry 2017[2]	Haiti	Middle & North Haiti	2014	0.1	210	13 (6.2)	0 (0.0)	0 (0.0)	197 (93.8)
**Asia & Pacific**									
Singh 2015[10]	India	Bastar	2007	2.1	1091	29 (2.7)	25 (2.3)	5 (0.5)	1032 (94.6)
Bardaji 2017[1]	India	Bikaner	2010	0.0	293	30 (10.2)	0 (0.0)	0 (0.0)	263 (89.8)
Singh 2015[10]	India	Rajnandgaon	2007	0.1	1386	30 (2.2)	0 (0.0)	0 (0.0)	1356 (97.8)
Pava 2016[12]	Indonesia	Timika	2013	4.8	44	8 (18.2)	4 (9.1)	0 (0.0)	32 (72.7)
Ahmed 2019[13]†	Indonesia	Timika	2015	24.8	142	142 (12.5)	33 (2.9)	42 (3.7)	915 (80.8)
Ahmed 2019[13]†	Indonesia	Sumba	2015	0.1	939	166 (17.7)	4 (0.4)	1 (0.1)	768 (81.8)
Unger 2015[11]	PNG	Yagaum	2011	2.1	175	8 (4.6)	14 (8.0)	3 (1.7)	150 (85.7)
Unger 2015[11]	PNG	Mugil	2011	0.5	182	16 (8.8)	6 (3.3)	3 (1.6)	157 (86.3)
Unger 2015[11]	PNG	Alexishafen	2011	1.4	374	36 (9.6)	34 (9.1)	5 (1.3)	299 (79.9)
Stanisic 2015[9]	PNG	Alexishafen	2006	0.0	350	122 (34.9)	113 (32.3)	7 (2.0)	108 (30.9)
Bardaji 2017[1]	PNG	Madang	2009	0.1	258	41 (15.9)	20 (7.8)	6 (2.3)	191 (74.0)
Unger 2015[11]	PNG	Madang	2011	0.5	1488	99 (6.7)	66 (4.4)	18 (1.2)	1305 (87.7)
**Africa**									
Cottrell 2015[26]	Benin	Come Central	2009	11.8	442	83 (18.8)	57 (12.9)	1 (0.2)	301 (68.1)
Cottrell 2015[26]	Benin	Ouedeme-Pedah	2009	16.4	165	47 (28.5)	33 (20.0)	1 (0.6)	84 (50.9)
Cottrell 2015[26]	Benin	Akodeha	2009	12.7	386	96 (24.9)	75 (19.4)	0 (0.0)	215 (55.7)
Accrombessi 2018[49]	Benin	Soava and Akassato	2015	20.6	367	108 (29.4)	17 (4.6)	10 (2.7)	232 (63.2)
Denoeud-Ndam 2013[34]	Benin	Porto Novo	2011	16.0	88	24 (27.3)	6 (6.8)	0 (0.0)	58 (65.9)
Denoeud-Ndam 2013[34]	Benin	Cotonou	2011	22.4	306	103 (33.7)	15 (4.9)	0 (0.0)	188 (61.4)
Williams 2016[35]	Burkina Faso	Ziniare	2011	47.3	693	116 (16.7)	282 (40.7)	15 (2.2)	280 (40.4)
Kattenberg 2012[31]	Burkina Faso	Nanoro	2011	63.1	380	92 (24.2)	109 (28.7)	3 (0.8)	176 (46.3)
Leke 2010[20]	Cameroon	Ngali II	2003	68.0	56	11 (19.6)	22 (39.3)	1 (1.8)	22 (39.3)
Ntoumi 2016[42]	Congo B	Madibou	2012	18.3	363	43 (11.8)	25 (6.9)	0 (0.0)	295 (81.3)
Williams 2016[35]	Gambia	Basse	2011	7.9	580	33 (5.7)	35 (6.0)	7 (1.2)	505 (87.1)

Study	Country	Location	Mid-year study	*Pf*PR2-10*		Microscopy vs. NAAT			
					Sample size	BS-/ NAAT+ (%)	BS+/ NAAT+ (%)	BS+/ NAAT- (%)	BS-/ NAAT- (%)
Mockenhaupt 2000[17]	Ghana	Agogo	1998	36.0	528	164 (31.1)	171 (32.4)	1 (0.2)	192 (36.4)
Williams 2016[35]	Ghana	Navrongo	2011	60.7	1258	141 (11.2)	548 (43.6)	52 (4.1)	517 (41.1)
Desai 2015[43]	Kenya	Siaya	2014	13.7	219	46 (21.0)	56 (25.6)	2 (0.9)	115 (52.5)
Desai 2015[43]	Kenya	Lwak	2013	11.2	344	91 (26.5)	54 (15.7)	1 (0.3)	198 (57.6)
Desai 2015[43]	Kenya	Bondo	2013	16.8	410	50 (12.2)	64 (15.6)	4 (1.0)	292 (71.2)
Daud 2015[38]	Kenya	Chulaimbo	2011	20.3	111	23 (20.7)	16 (14.4)	0 (0.0)	72 (64.9)
Desai 2015[43]	Kenya	Madiany	2013	13.5	456	54 (11.8)	57 (12.5)	1 (0.2)	344 (75.4)
Madanitsa 2016[39]‡	Malawi	Chikwawa-G12	2013	11.4	294	96 (32.7)	36 (12.2)	8 (2.7)	154 (52.4)
Madanitsa 2016[39]‡	Malawi	Chikwawa-G35	2013	11.4	97	15 (15.5)	3 (3.1)	1 (1.0)	78 (80.4)
Madanitsa 2016[39]‡	Malawi	Madziabango-G12	2012	18.9	242	83 (34.3)	59 (24.4)	11 (4.5)	89 (36.8)
Madanitsa 2016[39]‡	Malawi	Madziabango-G35	2012	18.9	205	58 (28.3)	22 (10.7)	12 (5.9)	113 (55.1)
Madanitsa 2016[39]‡	Malawi	Mpemba-G12	2012	18.0	569	210 (36.9)	85 (14.9)	16 (2.8)	258 (45.3)
Madanitsa 2016[39]‡	Malawi	Mpemba-G35	2012	18.0	381	89 (23.4)	23 (6.0)	11 (2.9)	258 (67.7)
Nkhoma 2017[40]	Malawi	Mangochi district	2012	27.9	1341	240 (17.9)	111 (8.3)	34 (2.5)	956 (71.3)
Cohee 2014[28]	Malawi	Blantyre	2010	23.7	438	46 (10.5)	54 (12.3)	0 (0.0)	338 (77.2)
Mahamar 2021[36]	Mali	Ouelessebougou	2013	49.6	1851	331 (17.9)	521 (28.2)	0 (0.0)	999 (54.0)
Williams 2016[35]	Mali	Kita	2011	31.1	180	27 (15.0)	19 (10.6)	2 (1.1)	132 (73.3)
Williams 2016[35]	Mali	San	2011	50.9	261	36 (13.8)	84 (32.2)	4 (1.5)	137 (52.5)
Williams 2016[35]	Mali	Bamako	2011	5.2	147	14 (9.5)	14 (9.5)	2 (1.4)	117 (79.6)
Kakuru 2016[47]†	Uganda	Tororo	2014	13.0	299	167 (55.9)	4 (1.3)	0 (0.0)	128 (42.8)
Briggs 2019[50]	Uganda	Busia district	2017	45.9	782	242 (30.9)	391 (50.0)	10 (1.3)	139 (17.8)
Chico 2017[89]	Zambia	Nchelenge	2013	37.5	1074	285 (26.5)	336 (31.3)	7 (0.7)	446 (41.5)

Abbreviations: BS: microscopy. LAMP: loop-mediated isothermal amplification. NAAT: nucleic acid amplification test. PCR: polymerase chain reaction. PNG: Papua New Guinea. G12: women in their first and second pregnancy. G35: women in their third to fifth pregnancy. For analyses in the main paper, the groups "microscopy positive/NAAT-negative" has been excluded.

**Pf*PR$_{2-10}$: Estimated prevalence in the study area for the mid-year of the study for *P. falciparum* among children 2-10 years of age (2020-layer, Malaria Atlas Project: https://malariaatlas.org). † LAMP was used in the following studies: Ahmed 2019,[13] Kakuru 2016,[47] Briggs,[50] Vasquez 2018[4] and Vasquez 2020[5]. ‡ Enrolment in this study was stratified by gravidity.

Several studies had enrolment criteria that may have affected parasite prevalence, these included (where known, in alphabetical order of first author): Ahmed 2019[13]: HIV-negative women, no severe malaria, no antimalarial treatment in previous month. Chico 2017[89]: No antimalarials or antibiotics in the previous 4 weeks. Daud 2014[38]: Hb >=7.5 g/dl. Denoeud-Ndam 2013[34]: HIV-infected women, last SP dose one month before enrolment, 2 weeks after any other antimalarial intake. Desai 2015[43]: HIV-negative women, no IPTp received yet, no severe anaemia (not further defined). Gavina 2018[3]: No antimalarial treatment in the past 2 weeks, no signs of severe malaria. Kakuru 2016[47]: HIV-negative women. Kapito-Tembo 2010:[55] HIV-infected women. Madanitsa 2016[39]: HIV-negative women, 1st ANC visit, Hb >7 g/dl, no IPTp received yet. Matangila 2014[61]: No fever or other symptoms of malaria. Ntoumi 2016[42]: No history of clinical malaria in the past 2 weeks, no fever for the past 48 hours, axillary temperature ≤37.5° at enrolment. Quakyi 2019[66]: HIV-negative women. Stanisic 2015[9]: 1st ANC visit, Hb ≥5 g/dl. Tadesse 2020[68]: No severe malaria symptoms, no antimalarials in the past 4 weeks. Unger 2015[90]: 1st ANC visit, Hb ≥6 g/dl. Vasquez 2018[4]: No antimalarials in the past three days. Vasquez 2020[5]: No history of malaria or antimalarial use in the past 3 months, no positive malaria test at previous ANC, no severe malaria. Williams 2016[35]: 1st ANC visit.

Table S8: Delivery: Prevalence of malaria by microscopy compared to NAATs by study (IPD, order by region and country)

Study	Country	Location	Mid-year study	*Pf*PR$_{2-10}$*	Microscopy vs. NAAT (maternal blood)					Microscopy vs. NAAT (placental blood)				
					Sample	BS-/NAAT+ (%)	BS+/ NAAT+ (%)	BS+/ NAAT- (%)	BS-/ NAAT- (%)	Sample	BS-/NAAT+ (%)	BS+/ NAAT+ (%)	BS+/ NAAT- (%)	BS-/ NAAT- (%)
**Americas**														
Bardaji 2017[1]	Brazil	Manaus	2010	0.0	287	15 (5.2)	0 (0.0)	0 (0.0)	272 (94.8)	17	0 (0.0)	0 (0.0)	0 (0.0)	17 (100.0)
Vasquez 2018[4]†	Colombia	Apartado/Turbo/El Bagre	2016	0.0	76	0 (0.0)	0 (0.0)	0 (0.0)	76 (100.0)	76	0 (0.0)	0 (0.0)	0 (0.0)	76 (100.0)
Gavina 2018[3]	Colombia	Puerto Libertador	2015	0.0	179	12 (6.7)	0 (0.0)	0 (0.0)	167 (93.3)	178	9 (5.1)	0 (0.0)	0 (0.0)	169 (94.9)
Vasquez 2018[4]†	Colombia	Quibdo	2016	1.2	123	0 (0.0)	0 (0.0)	0 (0.0)	123 (100.0)	123	2 (1.6)	1 (0.8)	0 (0.0)	120 (97.6)
Bardaji 2017[1]	Colombia	Tieralta	2010	0.0	300	12 (4.0)	3 (1.0)	0 (0.0)	285/300 (95.0)	18	1 (5.6)	0 (0.0)	0 (0.0)	17 (94.4)
Vasquez 2018[4]†	Colombia	Tumaco	2016	0.0	130	5 (3.8)	0 (0.0)	0 (0.0)	125 (96.2)	130	5 (3.8)	1 (0.8)	0 (0.0)	124 (95.4)
Bardaji 2017[1]	Guatemala	Fray Bartolome de las Casas	2010	0.0	300	54 (18.0)	0 (0.0)	0 (0.0)	246 (82.0)	2	0/2 (0.0)	0/2 (0.0)	0/2 (0.0)	2 (100.0)
**Asia**														
Singh 2015[10]	India	Bastar	2007	2.1						469	15 (3.2)	11 (2.3)	4 (0.9)	439 (93.6)
Bardaji 2017[1]	India	Bikaner	2010	0.0	292	29 (9.9)	0 (0.0)	0 (0.0)	263 (90.1)	98	18 (18.4)	0/98 (0.0)	0 (0.0)	80 (81.6)
Singh 2015[10]	India	Rajnandgaon	2007	0.0						479	14 (2.9)	0 (0.0)	1 (0.2)	464 (96.9)
Ahmed 2019[13]†	Indonesia	Sumba	2015	0.1	816	52 (6.4)	2 (0.2)	1 (0.1)	761 (93.3)	787	29 (3.7)	1 (0.1)	0 (0.0)	757 (96.2)
Ahmed 2019[13]†	Indonesia	Timika	2015	24.8	1037	64 (6.2)	21 (2.0)	14 (1.4)	938 (90.5)	976	39 (4.0)	16 (1.6)	6 (0.6)	915 (93.8)
Stanisic 2015[9]	PNG	Alexishafen	2006	0.0	309	107 (34.6)	28 (9.1)	11 (3.6)	163 (52.8)	232	76 (32.8)	34 (14.7)	6 (2.6)	116 (50.0)
Unger 2015[11]	PNG	Alexishafen	2011	1.4	345	23 (6.7)	15 (4.3)	3 (0.9)	304 (88.1)	215	5 (2.3)	11 (5.1)	1 (0.5)	198 (92.1)
Bardaji 2017[1]	PNG	Madang	2009	0.1	287	34 (11.8)	5 (1.7)	1 (0.3)	247 (86.1)	95	3 (3.2)	1 (1.1)	0 (0.0)	91 (95.8)
Unger 2015[11]	PNG	Madang	2011	0.5	1350	26 (1.9)	34 (2.5)	7 (0.5)	1283 (95.0)	994	15 (1.5)	21 (2.1)	4 (0.4)	954 (96.0)
Unger 2015[11]	PNG	Mugil	2011	0.5	148	7 (4.7)	2 (1.4)	0 (0.0)	139 (93.9)	40	0 (0.0)	0 (0.0)	0 (0.0)	40 (100.0)
Unger 2015[11]	PNG	Yagaum	2011	2.1	162	6 (3.7)	1 (0.6)	2 (1.2)	153 (94.4)	95	4 (4.2)	0 (0.0)	1 (1.1)	90 (94.7)
**Africa**														
Cottrell 2015[26]	Benin	Akodeha	2009	12.7	250	49 (19.6)	32 (12.8)	3 (1.2)	166 (66.4)	243	55 (22.6)	30 (12.3)	3 (1.2)	155 (63.8)
Gonzalez 2014[32]	Benin	Allada	2011	36.0	347	82 (23.6)	24 (6.9)	3 (0.9)	238 (68.6)	345	78 (22.6)	17 (4.9)	6 (1.7)	244 (70.7)
Cottrell 2015[26]	Benin	Come Central	2009	17.1	267	73 (27.3)	14 (5.2)	3 (1.1)	177 (66.3)	188	30 (16.0)	11 (5.9)	5 (2.7)	142 (75.5)
Denoeud-Ndam 2013[34]	Benin	Cotonou	2011	7.2	233	12 (5.2)	2 (0.9)	0 (0.0)	219 (94.0)	240	7 (2.9)	2 (0.8)	0 (0.0)	231 (96.3)
Cottrell 2015[26]	Benin	Ouedeme-Pedah	2009	11.7	134	37 (27.6)	11 (8.2)	1 (0.7)	85 (63.4)	113	34 (30.1)	15 (13.3)	2 (1.8)	62 (54.9)
Denoeud-Ndam 2013[34]	Benin	Porto Novo	2011	16.1	58	3 (5.2)	0 (0.0)	0 (0.0)	55 (94.8)	60	1 (1.7)	0 (0.0)	0 (0.0)	59 (98.3)
Accrombessi 2018[49]	Benin	Soava and Akassato	2015	20.6	233	24 (10.3)	9 (3.9)	0 (0.0)	200 (85.8)	221	9 (4.1)	6 (2.7)	8 (3.6)	198 (89.6)
Leke 2010[20]	Cameroon	Ngali II	2003	68.0	48	24 (50.0)	18 (37.5)	6 (12.5)	0 (0.0)					
Walker-Abbey 2005[16]	Cameroon	Yaounde	1997	51.5	302	138 (45.7)	91 (30.1)	4 (1.3)	69 (22.8)	198	70 (35.4)	69 (34.8)	2 (1.0)	57 (28.8)
Mbouamboua 2019[48]	Congo B	Madibou HC	2014	27.1	370	87 (23.5)	27 (7.3)	0 (0.0)	256 (69.2)	370	60 (16.2)	9 (2.4)	1 (0.3)	300 (81.1)
Landis 2009[24]	DRC	Kinshasa	2005	34.2	124	2 (1.6)	0 (0.0)	1 (0.8)	121 (97.6)					
Adegnika 2006[21]	Gabon	Lambarene	2003	29.0	130	22 (16.9)	9 (6.9)	0 (0.0)	99 (76.2)	81	8 (9.9)	8 (9.9)	0 (0.0)	65 (80.2)
Gonzalez 2014[32]	Gabon	Lambarene, Foukamou	2011	20.9	256	12 (4.7)	10 (3.9)	1 (0.4)	233 (91.0)	253	15 (5.9)	6 (2.4)	3 (1.2)	229 (90.5)
Mockenhaupt 2002[18]	Ghana	Agogo	2000	36.3	889	300 (33.7)	166 (18.7)	1 (0.1)	422 (47.5)	887	216 (24.4)	304 (34.3)	3 (0.3)	364 (41.0)
Williams 2016[35]	Ghana	Navrongo	2011	60.7	897	46 (5.1)	95 (10.6)	33 (3.7)	723 (80.6)					
Desai 2015[43]	Kenya	Bondo	2013	16.8	387	13 (3.4)	15 (3.9)	2 (0.5)	357 (92.2)	384	19 (4.9)	13 (3.4)	2 (0.5)	350 (91.1)
Daud 2015[38]	Kenya	Chulaimbo	2011	20.3	108	17 (15.7)	3 (2.8)	0 (0.0)	88 (81.5)					
Malhotra 2009[19]	Kenya	Kwale district	2002	81.6	629	180 (28.6)	37 (5.9)	14 (2.2)	398 (63.3)	583	94 (16.1)	2 (0.3)	4 (0.7)	483 (82.8)
Desai 2015[43]	Kenya	Lwak	2013	11.2	296	14 (4.7)	25 (8.4)	2 (0.7)	255 (86.1)	291	18 (6.2)	27 (9.3)	2 (0.7)	244 (83.8)
Desai 2015[43]	Kenya	Madiany	2013	13.5	408	10 (2.5)	10 (2.5)	1 (0.2)	387 (94.9)	398	15 (3.8)	10 (2.5)	1 (0.3)	372 (93.5)

Study	Country	Location	Mid-year study	*Pf*PR$_{2-10}$*	Microscopy vs. NAAT (maternal blood)					Microscopy vs. NAAT (placental blood)				
					Sample	BS-/NAAT+ (%)	BS+/ NAAT+ (%)	BS+/ NAAT- (%)	BS-/ NAAT- (%)	Sample	BS-/NAAT+ (%)	BS+/ NAAT+ (%)	BS+/ NAAT- (%)	BS-/ NAAT- (%)
Desai 2015[43]	Kenya	Siaya	2014	13.7	176	14 (8.0)	15 (8.5)	0 (0.0)	147 (83.5)	158	12 (7.6)	16 (10.1)	0 (0.0)	130 (82.3)
Gonzalez 2014[33] HIV+	Kenya	Siaya	2011	40.0	349	14 (4.0)	11 (3.2)	6 (1.7)	318 (91.1)	351	16 (4.6)	11 (3.1)	5 (1.4)	319 (90.9)
Cohee 2014[28]	Malawi	Blantyre	2010	23.7	289	7 (2.4)	3 (1.0)	0 (0.0)	279 (96.5)	305	63 (20.7)	4 (1.3)	0 (0.0)	238 (78.0)
Patel 2016[30]	Malawi	Blantyre	2010	23.7	162	4 (2.5)	1 (0.6)	4 (2.5)	153 (94.4)	187	4 (2.1)	3 (1.6)	0 (0.0)	180 (96.3)
Madanitsa 2016[39]‡	Malawi	Chikwawa-G12	2013	11.4	251	30 (12.0)	15 (6.0)	0 (0.0)	206 (82.1)	245	20 (8.2)	18 (7.3)	11 (4.5)	196 (80.0)
Madanitsa 2016[39]‡	Malawi	Chikwawa-G35	2013	11.4	90	8 (8.9)	2 (2.2)	0 (0.0)	80 (88.9)	82	11 (13.4)	3 (3.7)	2 (2.4)	66 (80.5)
Luntamo 2013[23]	Malawi	Lungwena	2005	31.5	453	39 (8.6)	11 (2.4)	3 (0.7)	400 (88.3)					
Madanitsa 2016[39]‡	Malawi	Madziabango-G12	2012	18.3	205	55 (26.8)	9 (4.4)	1 (0.5)	140 (68.3)	202	32 (15.8)	23 (11.4)	11 (5.4)	136 (67.3)
Madanitsa 2016[39]‡	Malawi	Madziabango-G35	2012	18.3	185	38 (20.5)	0 (0.0)	0 (0.0)	147 (79.5)	179	23 (12.8)	5 (2.8)	7 (3.9)	144 (80.4)
Patel 2016[30]	Malawi	Madziabango	2010	26.3	280	16 (5.7)	11 (3.9)	10 (3.6)	243 (86.8)	276	48 (17.4)	4 (1.4)	1 (0.4)	223 (80.8)
Nkhoma 2017[40]	Malawi	Mangochi district	2012	27.9										
Madanitsa 2016[39]‡	Malawi	Mpemba-G12	2012	18.0	482	111 (23.0)	5 (1.0)	1 (0.2)	365 (75.7)	467	78 (16.7)	24 (5.1)	27 (5.8)	338 (72.4)
Madanitsa 2016[39]‡	Malawi	Mpemba-G35	2012	18.0	328	43 (13.1)	1 (0.3)	0 (0.0)	284 (86.6)	315	34 (10.8)	8 (2.5)	17 (5.4)	256 (81.3)
Patel 2016[30]	Malawi	Mpemba	2010	27.8	661	26 (3.9)	21 (3.2)	10 (1.5)	604 (91.4)	655	59 (9.0)	15 (2.3)	0 (0.0)	581 (88.7)
Mahamar 2021[36]	Mali	Ouelessebougou	2013	49.6	1808	155 (8.6)	156 (8.6)	0 (0.0)	1497 (82.8)	1776	194 (10.9)	290 (16.3)	0 (0.0)	1292 (72.7)
Arnaldo 2018[46]	Mozambique	Chokwe district	2014	12.8	918	77 (8.4)	22 (2.4)	2 (0.2)	817 (89.0)					
Gonzalez 2014[32]	Mozambique	Manhica, Managra	2011	4.6	340	9 (2.6)	6 (1.8)	1 (0.3)	324 (95.3)	340	10 (2.9)	7 (2.1)	1 (0.3)	322 (94.7)
Gonzalez 2014[33] HIV+	Mozambique	Manhica, Managra	2011	4.6	416	5 (1.2)	7 (1.7)	1 (0.2)	403 (96.9)	413	4 (1.0)	6 (1.5)	1 (0.2)	402 (97.3)
Dobano 2018[25]	Mozambique	Manhica	2006	10.6	238	30 (12.6)	16 (6.7)	1 (0.4)	191 (80.3)					
Menendez 2008[22]	Mozambique	Manhica	2004	32.8	613	145 (23.7)	76 (12.4)	0 (0.0)	392 (63.9)	450	104 (23.1)	65 (14.4)	3 (0.7)	278 (61.8)
Oduwole 2011[27]	Nigeria	Calabar	2009	38.1	202	11 (5.4)	3 (1.5)	1 (0.5)	187 (92.6)	203	15 (7.4)	4 (2.0)	1 (0.5)	183 (90.1)
Mosha 2014[41]	Tanzania	Moshi	2012	4.2						174	2 (1.1)	1 (0.6)	1 (0.6)	170 (97.7)
Mosha 2014[41]	Tanzania	Rufiji	2012	17.1						174	15 (8.6)	12 (6.9)	1 (0.6)	146 (83.9)
Briggs 2019[50]†	Uganda	Busia district	2017	45.9						640	49 (7.7)	28 (4.4)	1 (0.2)	562 (87.8)
Braun 2015[45]	Uganda	Fort Portal	2013	13.5	743	57 (7.7)	22 (3.0)	1 (0.1)	663 (89.2)					
Kakuru 2016[47]†	Uganda	Tororo	2014	13.0	288	23 (8.0)	6 (2.1)	0 (0.0)	259 (89.9)	280	17 (6.1)	7 (2.5)	1 (0.4)	255 (91.1)
Arinaitwe 2013[37]	Uganda	Tororo	2011	38.0						565	62 (11.0)	97 (17.2)	2 (0.4)	404 (71.5)
Natureeba 2014[29]	Uganda	Tororo	2011	27.3						301	15 (5.0)	11 (3.7)	0 (0.0)	275 (91.4)

Abbreviations: BS: microscopy. LAMP: loop-mediated isothermal amplification. NAAT: nucleic acid amplification test. PCR: polymerase chain reaction. PNG: Papua New Guinea. G12: women in their first and second pregnancy. G35: women in their third to fifth pregnancy. For analyses in the main paper, the group "microscopy positive/NAAT-negative" has been excluded. * *Pf*PR$_{2-10}$: Estimated prevalence in the study area for the mid-year of the study for *P. falciparum* among children 2-10 years of age (2020-layer, Malaria Atlas Project: https://malariaatlas.org). † LAMP was used in the following studies: Ahmed 2019,[13] Kakuru 2016,[47] Vasquez 2018[4] and Briggs 2019.[50] ‡ Enrolment in this study was stratified by gravidity.

Table S9: Median and interquartile range of microscopy positive/NAAT negative test results overall and by region

	Microscopy positive, NAAT-negative results: Median, IQR, N [range]		
	Pregnancy	Delivery maternal	Delivery placental
**All**	0.5, 0.0-1.6, 56 [0.0-5.9]	0.2, 0.0-0.9, 58 [0.0-12.5]	0.4, 0.0-1.1, 57 [0.0-5.8]
**Americas**	0.0, 0.0-0.0, 11 [0.0-0.0]	0.0, 0.0-0.0, 7 [0.0-0.0]	0.0, 0.0-0.0, 7 [0.0-0.0]
**Asia**	1.3, 0.1-1.9, 12 [0.0-3.7]	0.5, 0.1-1.2, 9 [0.0-3.6]	0.4, 0.0-0.9, 11 [0.0-2.6]
**Africa**	1.0, 0.2-1.8, 33 [0.0-5.9]	0.3, 0.0-0.9, 42 [0.0-12.5]	0.5, 0.2-1.7, 39 [0.0-5.8]

IQR, interquartile range. N, number of substudies. NAAT, nucleic acid amplification test.

Table S10: Pregnancy: Pooled prevalence and odds ratio of fever by malaria test results

**Subgroup**	**N sub-Studies**	**Number of women in subgroups**	**Pooled prevalence of fever (95% CI)**	I^2 (%)	**Median prevalence fever, range**	**Pooled Odds Ratio (95% CI) Reference group “no malaria” (two-stage analysis)**	I^2 (%)	**Adjusted pooled Odds Ratio (95% CI) Reference group “no malaria” (one stage analysis)***	**p-value**	**Total N**	**Adjusted Odds Ratio (95% CI) Reference group “microscopic malaria” (one stage analysis)***	**p-value**	**Total N**
**Overall**													
Microscopic	36	1930	11.6 (6.2-17.9)	86.6	9.0, 0.0-100.0	**3.90 (2.60-5.85)**	62.0	**2.84 (2.30-3.51)**	<0.0001	17086	Reference		
Submicroscopic	39	2926	2.0 (0.4-4.3)	84.1	1.2, 0.0-36.4	**1.25 (1.02-1.54)**	0.0	**1.29 (1.04-1.60)**	0.0181		**0.46 (0.36-0.59)**	<0.0001	800
No malaria infection	40	13,326	3.9 (2.2-6.0)	96.6	3.8, 0.0-27.3	Reference		Reference					
**Americas/Asia**													
Microscopic	19	366	20.6 (8.2-35.7)	82.4	12.5, 0.0-100.0	**4.63 (2.58-8.30)**	59.6	**4.07 (2.91-5.69)**	<0.0001	9833	Reference		
Submicroscopic	22	897	0.9 (0.0-3.9)	71.7	0.5, 0.0-17.2	1.09 (0.75-1.59)	0.0	1.11 (0.76-1.61)	0.5913		**0.21 (0.13-0.35)**	<0.0001	1222
No malaria infection	23	8843	5.1 (2.5-8.5)	97.3	4.6, 0.0-19.6	Reference		Reference					
**Africa**													
Microscopic	17	1564	7.4 (2.7-13.5)	88.5	7.3, 0.0-100.0	**2.93 (1.79-4.82)**	51.3	**2.29 (1.75-3.00)**	<0.0001	7253	Reference		
Submicroscopic	17	2029	3.8 (1.3-7.4)	90.1	1.9, 0.0-36.4	**1.33 (1.04-1.70)**	0.0	**1.32 (1.02-1.72)**	0.0377		**0.61 (0.46-0.81)**	0.0006	578
No malaria infection	17	4483	2.4 (0.6-5.0)	94.6	1.1, 0.0-27.3	Reference		Reference					
**By transmission level (*Pf*PR$_{2-10}$) in Africa**													
**High (≥35%)**													
Microscopic	4	1104	19.7 (9.7-32.0)	94.2	18.8, 17.3-54.5	**2.15 (1.57-2.94)**	0.0	**2.16 (1.56-3.00)**	<0.0001	3177	Reference		
Submicroscopic	4	747	9.8 (3.9-17.7)	86.1	11.4, 3.9-36.4	1.28 (0.87-1.89)	0.0	1.42 (0.98-2.06)	0.0624		**0.64 (0.46-0.90)**	0.0096	1847
No malaria infection	4	1349	7.9 (3.2-14.3)	85.5	7.9, 3.7-27.3	Reference		Reference					
**Moderate to low (<35%)**													
Microscopic	13	460	3.6 (0.0-10.7)	81.0	0.0, 0.0-100.0	**5.38 (2.05-14.10)**	55.8	**2.75 (1.60-4.73)**	0.0003	4076	Reference		
Submicroscopic	13	1282	2.3 (0.1-6.2)	89.8	1.2, 0-23.1	1.36 (0.99-1.86)	0.0	1.34 (0.91-1.96)	0.1423		0.62 (0.37-1.03)	0.0646	1731
No malaria infection	13	3134	1.3 (0.0-4.1)	95.4	0.3, 0.0-21.2	Reference		Reference					

Abbreviations: aOR, adjusted odds ratio. CI, confidence interval. OR, Odds ratio.

Factors with a p-value <0.05 printed in bold

Notes: The following stata-procedures were used: pooled prevalence: metaprop, two-stage analyses OR: metan, one-stage analyses OR: xtlogit. Note that for the procedures meta-prop and metan, studies with 0-values in sample (meta-prop) or in the control group (metan) were not included in the meta-analyses which may explain some of the differences between median and pooled prevalence and pooled OR and aOR.

*Adjusted for region (Africa vs. Americas/Asia in overall model), gestational age, transmission level as continuous variable, season, study year, gravidity, age and first antenatal visit. The inclusion of gestational age led to a reduction in sample size (254 in overall model) but results with and without gestational age were similar (data not shown). An interaction was noted between malaria status and region (Africa vs. Americas/Asia combined, see table insert) for the overall model. P-values for interaction terms between malaria status and gestational age were ≥0.05 for each model (p=0.0661 for interaction term of model among parasitaemic women). Malaria species were not associated with fever in models among parasitaemic women by region (data not shown).

	aOR, 95% CI	p-value	aOR, 95% CI among NAAT+	p-value
No malaria, America/Asia	Reference			
Submicroscopic malaria, America/Asia	1.13, 0.78-1.64	0.5223	0.77, 0.20-2.96	0.7059
Microscopic malaria, America/Asia	4.08, 2.92-5.70	<0.0001	3.50, 0.91-13.38	0.0676
No malaria, Africa	0.38, 0.09-1.55	0.1758		
Submicroscopic malaria, Africa	0.50, 0.12-2.04	0.3318	0.60, 0.45-0.81	0.0007
Microscopic malaria, Africa	0.89, 0.22-3.65	0.8682	Reference	

Table S11: Pooled prevalence of species as assessed by PCR among microscopic and submicroscopic infections by region, meta-analysis

	Pregnancy									Delivery, maternal blood								
	Among microscopic infections					Among submicroscopic infections				Among microscopic infections					Among submicroscopic infections			
	N	Pooled estimate, 95% CI	I^2	Sub-studies	Median and range	Pooled estimate, 95% CI	I^2	Sub-studies	Median and range	N	Pooled estimate, 95% CI	I^2	Sub-studies	Median and range	Pooled estimate, 95% CI	I^2	Sub-studies	Median and range
**Americas**									**Americas**									
***P. falciparum* mono-infection**	88	69, 30-98	79%	8	75 (0-100)	47, 23-72	80%	8	35 (0-100)	21	67, 6-100		1	67 (67)	35, 2-79	93%	5	40 (0-100)
***P. vivax* mono-infection**	84	23, 1-56	71%	8	23 (0-100)	39, 13-68	85%	8	54 (0-100)	75	33, 0-94		1	33 (33)	56, 15-93	93%	5	53 (0-98)
**Mixed infections**	17	0, 0-5	0%	8	0 (0-25)	0, 0-8	55%	8	0 (0-22)	5	0, 0-50		1	0 (0)	3, 0-9	8%	5	7 (0-17)
**Other mono-infections**	2	0, 0-2	0%	8	0 (0)	0, 0-0	18%	8	0 (0-33)	0	0, 0-50		1	0 (0)	0, 0-1	0%	5	0 (0)
**Asia**									**Asia**									
***P. falciparum* mono-infection**	514	76, 62-87	78%	10	78 (30-100)	57, 43-69	88%	12	59 (20-93)	264	67, 48-84	50%	8	70 (29-100)	60, 41-77	90%	9	61 (21-100)
***P. vivax* mono-infection**	201	8, 4-13	16%	10	10 (0-25)	31, 22-41	79%	12	28 (7-59)	143	17, 9-27	0%	8	19 (0-40)	33, 18-51	89%	9	29 (0-77)
**Mixed infections**	118	9, 1-21	84%	10	7 (0-61)	7, 2-14	83%	12	3 (0-29)	43	4, 0-16	49%	8	0 (0-38)	4, 0-11	74%	9	3 (0-27)
**Other mono-infections**	34	0, 0-1	0%	10	0 (0-7)	1, 0-5	82%	12	0 (0-20)	5	0, 0-0	0%	8	0 (0-5)	0, 0-1	0%	9	0 (0-3)
**Africa***									**Africa**									
***P. falciparum* mono-infection**	1352	96, 91-99	86%	7	98 (85-100)	96, 90-100	81%	7	95 (77-100)	726	92, 79-99	86%	3	91 (85-98)	93, 83-99	90%	3	89 (88-98)
***P. vivax* mono-infection**	2	0, 0-0	0%	7	0 (0)	0, 0-1	27%	7	0 (0-15)	0	0, 0-0	0%	3	0 (0)	0, 0-0	0%	3	0 (0)
**Mixed infections**	25	1, 0-4	66%	7	1 (0-15)	1, 0-2	23%	7	0 (0-8)	40	8, 1-21	86%	3	9 (2-15)	4, 0-12	88%	3	4 (1-10)
**Other mono-infections**	8	0, 0-0	0%	7	0 (0-1)	0, 0-2	27%	7	0 (0-9)	8	0, 0-0	0%	3	0 (0)	2, 0-6	80%	3	1 (1-9)
**Asia/America**									**Asia/America combined**									
***P. falciparum* mono-infection**	602	75, 62-87	77%	18	75 (0-100)	54, 42-65	86%	20	52 (0-100)	285	67, 49-82	43%	9	68 (29-100)	51, 31-71	94%	14	48 (0-100)
***P. vivax* mono-infection**	285	9, 3-17	55%	18	13 (0-100)	34, 23-45	84%	20	32 (0-100)	218	18, 9-28	0%	9	27 (0-40)	41, 22-61	94%	14	33 (0-98)
**Mixed infections**	135	5, 0-13	74%	18	0 (0-61)	4, 0-9	76%	20	2 (0-29)	48	3, 0-15	42%	9	0 (0-38)	4, 1-9	63%	14	3 (0-27)
**Other mono-infections**	36	0, 0-0	0%	18	0 (0-7)	0, 0-2	73%	20	0 (0-33)	5	0, 0-0	0%	9	0 (0-5)	0, 0-0	0%	14	0 (0-3)

NA, not available. Notes: Pooled estimates using metaprop procedure, Stata. Note that because of the weighting of the studies in the random-effects models, proportion of *P. falciparum* and *P. vivax* are not adding up to 100.
*All regions in Africa combined because of limited sample in moderate-tolow transmission areas

Table S12: Malaria species as assessed by PCR as risk factor for submicroscopic malaria among PCR-positive infections, IPD*

	**Pregnancy, any dataset with species information**			**Pregnancy, datasets reporting ≥2 species**			**Delivery, any set with species information**			**Delivery, datasets reporting ≥2 species**		
	**aOR, 95% CI**	**p value**	**# Women, sub-locations**	**aOR, 95% CI**	**p value**	**# Women, sub-locations**	**aOR, 95% CI**	**p value**	**# Women, sub-locations**	**aOR, 95% CI**	**p value**	**# Women, sub-locations**
**Africa, high transmission**												
P. falciparum	Reference		1243, 4	Reference		1243, 4	Reference		461, 1	Reference		461, 1
P. vivax mono-infection	Insufficient data			Insufficient data			Insufficient data			Insufficient data		
Mixed infections†	0.86, 0.32-2.30	0.76		0.86, 0.32-2.30	0.76		0.47, 0.11-1.94	0.30		0.47, 0.11-1.94	0.30	
Other mono-infections‡	3.66, 0.80-16.67	0.09		3.66, 0.80-16.67	0.09		Insufficient data			Insufficient data		
**Africa, moderate-to-low transmission**												
P. falciparum	Reference ¶		134, 3	Reference ¶		24, 1	Reference		294, 2	Reference		294, 2
P. vivax mono-infection	Insufficient data			Insufficient data			Insufficient data			Insufficient data		
Mixed infections†	0.48, 0.04-5.40	0.55		0.55, 0.04-7.03			0.59, 0.27-1.27	0.18		0.59, 0.27-1.27	0.18	
Other mono-infections‡	Insufficient data			Insufficient data			Insufficient data			Insufficient data		
**Asia/Americas**												
P. falciparum mono-infection	Reference		1026, 20	Reference		969, 16	Reference		532, 13	Reference		503, 12
P. vivax mono-infection	**3.69, 2.45-5.54**	<0.0001		**3.45, 2.28-5.22**	<0.0001		**2.33, 1.31-4.15**	0.0042		**2.40, 1.35-4.27**	0.003	
Mixed infections†	1.40, 0.88-2.22	0.16		1.43, 0.89-2.28	0.14		0.86, 0.37-1.99	0.73		0.88, 0.38-2.03	0.76	
Other mono-infections‡	**7.86, 2.92-21.15**	<0.0001		**7.73, 2.87-20.79**	<0.0001		0.53, 0.08-3.38	0.50		0.54, 0.08-3.43	0.51	

aOR, adjusted Odds ratio. CI, confidence interval. IPD, individual participant data.

*Submicroscopic malaria as outcome of interest, with microscopic malaria as reference group, and species as exposure as a variable with 4 categories with *P. falciparum* as the reference category. Models were adjusted for variables in the baseline model (age, gravidity, season, level of malaria transmission and 1st antenatal visit for enrolment and study design at delivery). †Mixed infections: combinations of *P. falciparum*, *P. vivax*, *P. malariae* or *P. ovale.* ‡Other mono-infections: mono-infections with *P. ovale* or *P. malariae* § Comparison: *P. vivax* versus any other infection, univariate analyses, because of small sample size ¶ Pregnancy: univariate analyses because of small sample size

Table S13: Multivariable analyses of factors associated with submicroscopic and microscopic infections by gravidity, base model

**Africa, high transmission**								
	**Any malaria infection vs. no malaria**		**Microscopic vs. no infection**		**Submicroscopic vs. no infection**		**Risk of submicroscopic infections among NAAT-positive infections**	
	**aOR, 95% CI**	**p-value**	**aOR, 95% CI**	**p-value**	**aOR, 95% CI**	**p-value**	**aOR, 95% CI**	**p-value**
**Primigravidae** N=2171								
Age (years)								
<20	**5.52, 2.17-14.07**	0.0003	**6.29, 2.21-17.91**	0.0006	3.59, 0.79-16.26	0.10	0.57, 0.11-2.98	0.51
20-29	**3.03, 1.19-7.73**	0.0206	**3.16, 1.11-9.03**	0.0316	2.70, 0.59-12.27	0.20	0.85, 0.16-4.49	0.85
30+ years	Reference		Reference		Reference		Reference	
*Pf*PR$_{2-10}$	**1.04, 1.01-1.06**	0.0036	**1.04, 1.02-1.06**	0.0010	1.02, 0.99-1.05	0.18	**0.98, 0.96-1.00**	0.0114
Rainy season	1.05, 0.86-1.28	0.65	1.06, 0.86-1.31	0.59	1.01, 0.74-1.37	0.97	0.95, 0.71-1.27	0.73
1st ANC visit	**0.44, 0.22-0.89**	0.0222	**0.46, 0.23-0.92**	0.0294	**0.39, 0.18-0.85**	0.0171	0.86, 0.62-1.19	0.36
**Secundigravidae** N=1798								
Age (years)								
<20	**3.12, 1.82-5.36**	<0.0001	**2.86, 1.59-5.13**	0.0004	**4.25, 1.78-10.11**	0.0011	1.49, 0.61-3.66	0.38
20-29	**1.65, 1.01-2.72**	0.0471	1.42, 0.83-2.44	0.21	**2.45, 1.07-5.58**	0.0332	1.73, 0.72-4.12	0.22
30+ years	Reference		Reference		Reference		Reference	
*Pf*PR$_{2-10}$	1.02, 1.00-1.05	0.10	**1.04, 1.01-1.07**	0.0057	1.00, 0.97-1.03	0.87	**0.97, 0.95-0.98**	<0.0001
Rainy season	1.06, 0.86-1.30	0.61	1.23, 0.98-1.55	0.08	0.76, 0.57-1.01	0.06	**0.62, 0.46-0.82**	0.0011
1st ANC visit	**0.41, 0.19-0.87**	0.0201	0.38, 0.14-1.05	0.06	0.47, 0.15-1.53	0.21	1.23, 0.86-1.75	0.26
**Multigravidae (G3+)** N=2382								
Age (years)								
<20	1.61, 0.87-2.99	0.13	**2.92, 1.49-5.72**	0.0019	0.80, 0.34-1.86	0.60	**0.27, 0.12-0.64**	0.0028
20-29	**1.26, 1.06-1.50**	0.0086	**1.61, 1.30-2.01**	<0.0001	1.05, 0.86-1.28	0.66	**0.65, 0.51-0.82**	0.0003
30+ years	Reference		Reference		Reference		Reference	
*Pf*PR$_{2-10}$	1.01, 0.99-1.03	0.37	**1.02, 1.00-1.04**	0.0167	1.00, 0.97-1.02	0.77	**0.97, 0.96-0.99**	<0.0001
Rainy season	**1.56, 1.24-1.95**	0.0001	**1.95, 1.47-2.58**	<0.0001	1.27, 0.98-1.65	0.08	**0.65, 0.48-0.87**	0.0042
1st ANC visit	Reference		Not included		Not included		Not included	
**Africa, moderate-to-low transmission**								
	**Any malaria infection vs. no malaria**		**Microscopic vs. no infection**		**Submicroscopic vs. no infection**		**Risk of submicroscopic infections among NAAT-positive infections**	
	**aOR, 95% CI**	**p-value**	**aOR, 95% CI**	**p-value**	**aOR, 95% CI**	**p-value**	**aOR, 95% CI**	**p-value**
**Primigravidae** N=2605								
Age (years)								
<20	1.06, 0.42-2.68	0.91	2.27, 0.47-10.93	0.31	0.75, 0.27-2.07	0.57	0.33, 0.06-1.69	0.18
20-29	0.53, 0.21-1.35	0.18	1.08, 0.22-5.21	0.92	0.39, 0.14-1.09	0.07	0.36, 0.07-1.89	0.23
30+ years	Reference		Reference		Reference		Reference	

*Pf*PR2-10	**0.96, 0.93-0.99**	0.0126	**0.96, 0.92-1.00**	0.0496	**0.96, 0.92-1.00**	0.0345	1.00, 0.95-1.04	0.83
Rainy season	0.94, 0.78-1.13	0.49	0.94, 0.74-1.18	0.57	0.93, 0.74-1.17	0.54	1.00, 0.76-1.31	0.98
1st ANC visit	0.65, 0.30-1.43	0.29	0.56, 0.24-1.29	0.17	0.86, 0.35-2.12	0.74	1.54, 0.64-3.71	0.34
**Secundigravidae** N=2087								
Age (years)								
<20	**3.18, 1.62-6.25**	0.0008	**7.24, 1.67-31.34**	0.0081	**2.44, 1.17-5.09**	0.0171	0.34, 0.07-1.63	0.18
20-29	**2.09, 1.09-4.01**	0.0262	4.00, 0.94-16.98	0.06	1.75, 0.87-3.53	0.12	0.44, 0.09-2.06	0.30
30+ years	Reference		Reference		Reference		Reference	
*Pf*PR2-10	0.98, 0.95-1.02	0.34	0.98, 0.94-1.03	0.44	0.99, 0.95-1.03	0.52	1.00, 0.96-1.05	0.88
Rainy season	0.97, 0.79-1.20	0.81	0.97, 0.71-1.34	0.86	0.97, 0.76-1.24	0.82	1.00, 0.70-1.44	0.99
1st ANC visit	0.61, 0.32-1.18	0.14	**0.47, 0.24-0.95**	0.0355	0.72, 0.34-1.53	0.39	1.52, 0.67-3.41	0.32
**Multigravidae (G3+)** N=3507								
Age (years)								
<20	1.58, 0.87-2.86	0.13	1.27, 0.38-4.27	0.70	1.64, 0.86-3.11	0.13	1.29, 0.36-4.66	0.70
20-29	**1.25, 1.06-1.47**	0.0073	**1.52, 1.14-2.04**	0.0048	1.17, 0.97-1.40	0.10	0.76, 0.56-1.05	0.10
30+ years	Reference		Reference		Reference		Reference	
*Pf*PR2-10	0.98, 0.95-1.01	0.12	0.98, 0.94-1.02	0.32	0.98, 0.94-1.01	0.14	1.00, 0.95-1.04	0.87
Rainy season	0.97, 0.82-1.15	0.71	0.92, 0.69-1.23	0.58	0.98, 0.81-1.18	0.83	1.06, 0.77-1.46	0.71
1st ANC visit	1.01, 0.55-1.84	0.98	0.78, 0.40-1.53	0.47	1.12, 0.57-2.19	0.75	1.43, 0.70-2.91	0.33

**Asia and Americas**								
	**Any malaria infection vs. no malaria**		**Microscopic vs. no infection**		**Submicroscopic vs. no infection**		**Risk of submicroscopic infections among NAAT-positive infections**	
	**aOR, 95% CI**	**p-value**	**aOR, 95% CI**	**p-value**	**aOR, 95% CI**	**p-value**	**aOR, 95% CI**	**p-value**
**Primigravidae** N=3894								
Age (years)								
<20	1.46, 0.80-2.64	0.21	2.02, 0.67-6.08	0.21	1.27, 0.65-2.46	0.48	0.63, 0.19-2.11	0.45
20-29	1.14, 0.64-2.04	0.66	1.30, 0.44-3.87	0.63	1.11, 0.58-2.11	0.75	0.85, 0.26-2.81	0.79
30+ years	Reference		Reference		Reference		Reference	
*Pf*PR2-10	1.01, 0.97-1.06	0.53	1.03, 0.96-1.10	0.44	1.01, 0.96-1.06	0.66	0.98, 0.91-1.06	0.67
Rainy season	1.06, 0.84-1.33	0.65	1.08, 0.76-1.52	0.67	1.05, 0.80-1.38	0.73	0.97, 0.65-1.46	0.89
1st ANC visit	**3.61, 1.28-10.21**	0.0156	**8.83, 2.37-32.93**	0.0012	2.67, 0.82-8.68	0.10	0.30, 0.08-1.14	0.08
**Secundigravidae** N=2636								
Age (years)								
<20	**2.22, 1.26-3.89**	0.0056	**4.27, 1.33-13.71**	0.0148	1.82, 0.97-3.42	0.06	0.43, 0.12-1.53	0.19
20-29	1.43, 0.92-2.23	0.11	1.95, 0.68-5.58	0.21	1.35, 0.84-2.18	0.22	0.69, 0.22-2.14	0.52
30+ years	Reference		Reference		Reference		Reference	
*Pf*PR2-10	**1.06, 1.01-1.11**	0.0117	1.03, 0.97-1.10	0.33	**1.08, 1.02-1.15**	0.0089	1.05, 0.97-1.13	0.22
Rainy season	0.94, 0.68-1.30	0.69	1.70, 0.94-3.06	0.08	0.79, 0.55-1.14	0.21	**0.47, 0.24-0.89**	0.0211
1st ANC visit	3.10, 0.95-10.12	0.06	**8.54, 2.54-28.75**	0.0005	1.96, 0.51-7.56	0.33	**0.23, 0.07-0.72**	0.0121

**Multigravidae (G3+)** N=3538								
Age (years)								
<20	1.63, 0.81-3.30	0.17	**3.91, 1.22-12.50**	0.0215	1.23, 0.53-2.84	0.63	0.31, 0.08-1.24	0.10
20-29	1.09, 0.87-1.35	0.46	1.54, 0.98-2.40	0.06	0.99, 0.78-1.26	0.96	0.65, 0.40-1.05	0.08
30+ years	Reference		Reference		Reference		Reference	
*Pf*PR$_{2\text{-}10}$	**1.04, 1.01-1.08**	0.0165	0.99, 0.93-1.05	0.73	**1.06, 1.02-1.10**	0.0053	1.07, 1.00-1.15	0.06
Rainy season	1.00, 0.78-1.28	0.98	1.47, 0.90-2.43	0.13	0.92, 0.70-1.20	0.53	0.62, 0.36-1.07	0.09
1st ANC visit	2.05, 0.79-5.32	0.14	**5.25, 1.50-18.41**	0.0095	1.75, 0.61-5.04	0.30	0.33, 0.08-1.40	0.13

ANC, antenatal clinic. aOR, adjusted Odds ratio. CI, confidence interval. G1, primigravidae. G2, secundigravidae. G3+, multigravidae (excluding secundigravidae). PfPR$_{2\text{-}10}$, Plasmodium falciparum prevalence among children aged 2-10 years at the location and year of study visit, as estimated by the Malaria Atlas Project. Factors where the p-value is <0·05 are printed in bold. Note that studies in Asia and the Pacific were included under “Asia”; studies in central or south America were included under “Americas”.

Notes: Results for first ANC visits may have been affected by the data available, with only one study including first ANC visits only in high transmission areas for which only women in their first and second pregnancy were eligible; they had a lower prevalence of malaria compared to the other studies in this group,. In Asia/Americas, two studies included first ANC visits only and these were conducted in areas with higher malaria transmission than the other studies in this group.

Table S14: Multivariable analyses of factors associated with submicroscopic and microscopic *Plasmodium falciparum* and *Plasmodium vivax* infection in Asia and America, IPD base model

	Any malaria infection vs. no malaria		Microscopic vs. no infection		Submicroscopic vs. no infection		Risk of submicroscopic infections among NAAT-positive infections	
	aOR, 95% CI	p-value	aOR, 95% CI	p-value	aOR, 95% CI	p-value	aOR, 95% CI	p-value
**America and Asia: *Plasmodium falciparum*** N=8192								
Age (years)								
<20	**2.15, 1.46-3.17**	<0.0001	**2.59, 1.42-4.74**	0.0020	**1.84, 1.13-2.98**	0.0136	0.71, 0.34-1.49	0.37
20-29	**1.66, 1.22-2.25**	0.0011	1.65, 0.99-2.73	0.05	**1.65, 1.15-2.37**	0.0064	1.00, 0.55-1.83	0.99
30+ years	Reference		Reference		Reference		Reference	
Gravidity								
G1	1.09, 0.84-1.41	0.52	**1.52, 1.01-2.29**	0.0435	0.88, 0.64-1.22	0.45	0.58, 0.35-0.95	0.0307
G2	1.00, 0.76-1.32	1.00	1.19, 0.76-1.88	0.45	0.91, 0.66-1.27	0.59	0.77, 0.45-1.31	0.33
G1 /G2*	1.05, 0.83-1.33	0.69	1.39, 0.95-2.04	0.09	0.90, 0.67-1.19	0.45	0.65, 0.41-1.02	0.06
G3+	Reference		Reference		Reference		Reference	
*Pf*PR$_{2-10}$	1.02, 0.98-1.06	0.26	0.95, 0.89-1.00	0.07	**1.06, 1.01-1.12**	0.0230	1.12, 1.04-1.21	0.0043
Rainy season	**1.28, 1.04-1.57**	0.0197	**1.40, 1.03-1.89**	0.0293	1.19, 0.91-1.54	0.20	0.85, 0.58-1.24	0.39
1st ANC visit	**4.76, 1.78-12.70**	0.0019	**14.08, 2.01-98.84**	0.0078	**3.71, 1.30-10.54**	0.0140	0.26, 0.03-2.32	0.23
America vs. Asia	0.85, 0.30-2.37	0.75	0.46, 0.05-4.04	0.48	1.09, 0.36-3.29	0.88	2.37, 0.21-26.36	0.48
**America and Asia: *Plasmodium vivax*** N=7905								
Age (years)								
<20	1.45, 0.88-2.38	0.15	2.15, 0.58-8.04	0.26	1.35, 0.79-2.31	0.27	0.63, 0.15-2.57	0.52
20-29	1.15, 0.80-1.64	0.45	2.34, 0.91-6.02	0.08	1.01, 0.69-1.48	0.96	0.43, 0.16-1.19	0.10
30+ years	Reference		Reference		Reference		Reference	
Gravidity								
G1	0.71, 0.50-1.01	0.06	0.54, 0.24-1.20	0.10	0.76, 0.51-1.12	0.16	1.40, 0.58-3.36	0.45
G2	0.87, 0.61-1.24	0.45	0.54, 0.22-1.29	0.17	0.96, 0.65-1.41	0.84	1.78, 0.69-4.59	0.23
G1 /G2*	0.78, 0.58-1.07	0.12	0.54, 0.27-1.09	0.09	0.85, 0.61-1.19	0.35	1.57, 0.73-3.40	0.25
G3+	Reference		Reference		Reference		Reference	
*Pf*PR$_{2-10}$	1.05, 0.98-1.12	0.18	1.06, 0.95-1.18	0.32	1.04, 0.97-1.12	0.22	0.99, 0.89-1.10	0.81
Rainy season	0.97, 0.73-1.27	0.80	0.96, 0.50-1.84	0.90	0.97, 0.72-1.30	0.83	1.01, 0.50-2.06	0.98
1st ANC visit	2.17, 0.57-8.30	0.26	5.30, 0.95-29.38	0.20	1.85, 0.45-7.54	0.39	0.35, 0.08-1.44	0.15
America vs. Asia	1.93, 0.50-7.51	0.34	4.30, 0.68-27.37	0.12	1.55, 0.36-6.67	0.55	0.35, 0.08-1.44	0.23

OR, adjusted Odds ratio. CI, confidence interval. G1, primigravidae. G2, secundigravidae. G3+, multigravidae (excluding secundigravidae). PfPR$_{2-10}$, Plasmodium falciparum prevalence among children aged 2-10 years at the location and year of study visit, as estimated by the Malaria Atlas Project. Factors where the p-value is <0·05 are printed in bold. Note that studies in Asia and the Pacific were included under "Asia"; studies in central or south America were included under "Americas". *The line for combination of G1 and G2 was added to allow the assessment of the effect size of the combination of these groups, using the same reference group (G3+) and the same base model. Note that this estimate is from a separate model.

Table S15: Multivariable analyses of factors associated with submicroscopic and microscopic malaria infections in pregnancy by region, first ANC visits only, IPD base model

**Africa, high transmission level*, first ANC visits**								
	**Any malaria infection vs. no malaria**		**Microscopic vs. no malaria**		**Submicroscopic vs. no malaria**		**Risk of submicroscopic infections among NAAT-positive infections**	
**N=2130** 31·6% of available data, 3 sublocations†								
	**aOR, 95% CI**	**p-value**	**aOR, 95% CI**	**p-value**	**aOR, 95% CI**	**p-value**	**aOR, 95% CI**	**p-value**
Age (years)								
<20	**2.10, 1.20-3.69**	0.0094	**1.95, 1.07-3.54**	0.0290	**2.73, 1.01-7.41**	0.0479	1.40, 0.50-3.92	0.52
20-29	1.09, 0.64-1.87	0.74	0.93, 0.53-1.66	0.81	1.77, 0.68-4.63	0.25	1.89, 0.70-5.14	0.21
30+ years	Reference		Reference		Reference		Reference	
Gravidity								
G1	**1.45, 1.19-1.78**	0.0002	**1.76, 1.42-2.18**	<0.0001	0.84, 0.61-1.14	0.25	**0.48, 0.35-0.65**	<0.0001
G2	Reference		Reference		Reference		Reference	
G3+	Not present		Not present		Not present		Not present	
*Pf*PR$_{2-10}$*	Not included‡		Not included‡		Not included‡		Not included‡	
Rainy vs. dry season	0.92, 0.77-1.10	0.35	0.97, 0.80-1.17	0.74	0.78, 0.60-1.02	0.07	0.81, 0.62-1.06	0.12
**Africa, Moderate and low transmission level*, first ANC visits**								
	**Any malaria infection vs. no malaria**		**Microscopic vs. no malaria**		**Submicroscopic vs. no malaria**		**Risk of submicroscopic infections among NAAT-positive infections**	
**N=2982** 35·7% of available data, 10 sublocations†								
	**aOR, 95% CI**	**p-value**	**aOR, 95% CI**	**p-value**	**aOR, 95% CI**	**p-value**	**aOR, 95% CI**	**p-value**
Age (years)								
<20	**2.07, 1.43-3.01**	0.0001	**2.53, 1.31-4.90**	0.0058	**1.93, 1.28-2.90**	0.0017	0.72, 0.36-1.47	0.37
20-29	1.19, 0.89-1.60	0.24	1.29, 0.73-2.29	0.38	1.17, 0.85-1.61	0.33	0.89, 0.49-1.63	0.71
30+ years	Reference		Reference		Reference		Reference	
Gravidity								
G1	1.18, 0.70-1.97	0.54	**2.07, 1.06-4.05**	0.0324	0.97, 0.56-1.69	0.92	**0.43, 0.24-0.77**	0.0048
G2	0.83, 0.50-1.36	0.45	0.98, 0.51-1.87	0.95	0.83, 0.49-1.40	0.48	0.77, 0.44-1.33	0.35
G3+	Reference		Reference		Reference		Reference	
*Pf*PR$_{2-10}$	**0.94, 0.91-0.98**	0.0019	**0.94, 0.90-0.98**	0.0056	**0.95, 0.91-0.98**	0.0059	1.01, 0.97-1.06	0.48
Rainy vs. dry season	0.89, 0.74-1.07	0.21	0.99, 0.76-1.30	0.96	0.84, 0.69-1.04	0.10	0.91, 0.68-1.20	0.50
**Asia, first ANC visits (none in Americas)**								
	**Any malaria infection vs. no malaria**		**Microscopic vs. no malaria**		**Submicroscopic vs. no malaria**		**Risk of submicroscopic infections among NAAT-positive infections**	
**N=2516** 24·4% of available data, 5 sublocations†								
	**aOR, 95% CI**	**p-value**	**aOR, 95% CI**	**p-value**	**aOR, 95% CI**	**p-value**	**aOR, 95% CI**	**p-value**
Age (years)								

<20	**2.11, 1.57-2.84**	<0.0001	**2.60, 1.37-4.93**	0.0034	1.44, 0.82-2.53	0.21	0.55, 0.26-1.17	0.12
20-29	1.16, 0.90-1.50	0.26	1.58, 0.93-2.68	0.09	1.21, 0.78-1.85	0.39	0.76, 0.42-1.39	0.38
30+ years	Reference		Reference		Reference		Reference	
Gravidity								
G1	1.27, 0.79-2.03	0.32	1.50, 0.97-2.32	0.07	1.15, 0.78-1.70	0.48	0.77, 0.46-1.27	0.30
G2	0.88, 0.55-1.39	0.58	1.32, 0.81-2.16	0.26	1.41, 0.93-2.14	0.10	1.07, 0.61-1.86	0.82
G3+	Reference		Reference		Reference		Reference	
Pf$PR_{2\text{-}10}$*	Not included		Not included		Not included		Not included	
Rainy vs· dry season	0.95, 0.84-1.07	0.38	1.18, 0.87-1.59	0.29	1.38, 1.04-1.82	0.0236	1.17, 0.82-1.68	0.39

ANC, antenatal clinic. aOR, adjusted Odds ratio. CI, confidence interval. G1, primigravidae. G2, secundigravidae. G3+, multigravidae (excluding secundigravidae). Available data: number of participants with an outcome on submicroscopic, microscopic and no malaria. Factors where the p-value is <0·05 are printed in bold. Note that studies in Asia and the Pacific were included under “Asia”.
*Transmission level: Africa, high transmission: *Pf*$PR_{2\text{-}10}$ ≥35%; Africa, moderate-to-low transmission: *Pf*$PR_{2\text{-}10}$ <35%: only 1 study (two sublocations) *Pf*$PR_{2\text{-}10}$ <10%. In the model for America/Asia, *Pf*$PR_{2\text{-}10}$ was included as a continuous variable in the models. In the high transmission area in Africa, *Pf*$PR_{2\text{-}10}$ was not included because of non-convergence of the models; there were 3 sublocations with *Pf*$PR_{2\text{-}10}$ range of 47-63%. In Asia, *Pf*$PR_{2\text{-}10}$ was not included because of the limited range of *Pf*$PR_{2\text{-}10}$ (0-4%) with only 174 participants when *Pf*$PR_{2\text{-}10}$ in the range of 2-4%, leading to distortion of results (e.g., for submicroscopic malaria vs. no malaria the aOR was 0.59, 0.45-0.78, suggesting a jump of 41% in odds for every percentage increase of *Pf*$PR_{2\text{-}10}$ which is improbable). Removal of *Pf*$PR_{2\text{-}10}$ did not lead to meaningful differences for the estimates of the other co-variates. †Available data (data with information on microscopic, submicroscopic and no malaria): Africa, high transmission areas N=6746, 9 sublocations (microscopic 2455, submicroscopic 1406, no malaria infections 2885); Africa, moderate-to-low transmission areas N=8350, 25 sublocations (microscopic 949, submicroscopic 1858, no malaria infection 5543); Americas/Asia N=10,305, 23 sublocations (microscopic 373, submicroscopic 919, no malaria infection 9013).

Table S16: Multivariable models by region to assess the effect of inclusion of the variable "Gestational age" into the analyses of submicroscopic and microscopic malaria infections, IPD

**Africa, high transmission level, gestational age as co-variate, N=6371**												
	**Submicroscopic vs. no infection**				**Microscopic vs. no infection**				**Submicroscopic vs. microscopic infection**			
	**Base model**		**Base model + factor of interest**		**Base model**		**Base model + factor of interest**		**Base model**		**Base model + factor of interest**	
	**aOR, 95% CI**	**p-value**	**aOR, 95% CI**	**p-value**	**aOR, 95% CI**	**p-value**	**aOR, 95% CI**	**p-value**	**aOR, 95% CI**	**p-value**	**aOR, 95% CI**	**p-value**
Age (years)												
<20	**2.00, 1.41-2.84**	0.0001	**2.00, 1.41-2.84**	0.0001	**4.50, 3.16-6.39**	<0.0001	**4.49, 3.16-6.39**	<0.0001	**0.44, 0.30-0.67**	0.0001	**0.45, 0.30-0.67**	0.0001
20-24	**1.44, 1.07-1.93**	0.0161	**1.44, 1.07-1.93**	0.0161	**2.40, 1.74-3.33**	<0.0001	**2.40, 1.74-3.32**	<0.0001	**0.60, 0.42-0.86**	0.0054	**0.60, 0.42-0.86**	0.0055
25-29	1.33, 1.00-1.77	0.05	1.33, 1.00-1.77	0.05	**1.71, 1.24-2.36**	0.0011	**1.71, 1.24-2.36**	0.0011	0.78, 0.54-1.11	0.16	0.78, 0.54-1.11	0.17
30-34	**1.37, 1.01-1.85**	0.0400	**1.37, 1.01-1.85**	0.0400	1.37, 0.97-1.94	0.07	1.37, 0.97-1.94	0.07	1.00, 0.68-1.46	0.99	1.00, 0.68-1.46	0.99
35+ years	Reference		Reference		Reference		Reference		Reference		Reference	
Gravidity												
G1	**0.62, 0.48-0.81**	0.0004	**0.62, 0.48-0.81**	0.0004	**1.60, 1.29-1.99**	<0.0001	**1.60, 1.29-1.99**	<0.0001	**0.39, 0.30-0.51**	<0.0001	**0.39, 0.30-0.51**	<0.0001
G2	**0.71, 0.57-0.88**	0.0018	**0.71, 0.57-0.88**	0.0018	1.09, 0.89-1.32	0.40	1.09, 0.89-1.32	0.40	**0.65, 0.52-0.81**	0.0002	**0.65, 0.52-0.81**	0.0002
G3+	Reference		Reference		Reference		Reference		Reference		Reference	
Rainy season	1.02, 0.87-1.20	0.79	1.02, 0.87-1.20	0.79	**1.28, 1.12-1.46**	0.0004	**1.28, 1.12-1.46**	0.0004	**0.80, 0.68-0.94**	0.0085	**0.80, 0.68-0.94**	0.0084
1st ANC visit	**0.48, 0.24-0.97**	0.0394	**0.48, 0.24-0.97**	0.0394	0.60, 0.28-1.26	0.17	0.60, 0.29-1.25	0.17	0.81, 0.57-1.15	0.24	0.81, 0.57-1.14	0.23
Gestational age (weeks)	Not included		1.00, 0.99-1.01	0.84	Not included		1.00, 0.99-1.01	0.55	Not included		1.00, 0.99-1.02	0.75
**Africa, moderate-to-low transmission level, gestational age as co-variate, N=7364**												
	**Submicroscopic vs. no infection**				**Microscopic vs. no infection**				**Submicroscopic vs. microscopic infection**			
	**Base model**		**Base model + factor of interest**		**Base model**		**Base model + factor of interest**		**Base model**		**Base model + factor of interest**	
	**aOR, 95% CI**	**p-value**	**aOR, 95% CI**	**p-value**	**aOR, 95% CI**	**p-value**	**aOR, 95% CI**	**p-value**	**aOR, 95% CI**	**p-value**	**aOR, 95% CI**	**p-value**
Age (years)												
<20	**2.08, 1.51-2.89**	<0.0001	**2.09, 1.51-2.89**	<0.0001	**4.47, 2.69-7.43**	<0.0001	**4.50, 2.71-7.48**	<0.0001	**0.47, 0.27-0.81**	0.0072	**0.46, 0.27-0.81**	0.0068
20-24	1.29, 0.97-1.72	0.08	1.29, 0.96-1.72	0.09	**2.34, 1.45-3.78**	0.0005	**2.33, 1.45-3.77**	0.0005	**0.55, 0.33-0.93**	0.0254	**0.55, 0.33-0.93**	0.0256
25-29	1.18, 0.89-1.55	0.25	1.17, 0.89-1.54	0.25	**1.65, 1.03-2.63**	0.0373	**1.64, 1.03-2.62**	0.0385	0.71, 0.43-1.19	0.20	0.72, 0.43-1.19	0.20
30-34	1.08, 0.81-1.45	0.61	1.08, 0.81-1.45	0.60	1.22, 0.73-2.03	0.44	1.22, 0.73-2.04	0.44	0.88, 0.51-1.54	0.66	0.89, 0.51-1.54	0.67
35+ years	Reference		Reference		Reference		Reference		Reference		Reference	
Gravidity												
G1	1.06, 0.85-1.33	0.59	1.07, 0.85-1.34	0.56	**1.89, 1.42-2.52**	<0.0001	**1.91, 1.43-2.54**	<0.0001	**0.56, 0.41-0.78**	0.0005	**0.56, 0.40-0.78**	0.0005
G2	1.02, 0.84-1.25	0.81	1.03, 0.85-1.26	0.76	1.14, 0.87-1.51	0.34	1.16, 0.88-1.52	0.30	0.90, 0.66-1.22	0.48	0.89, 0.66-1.21	0.47
G3+	Reference		Reference		Reference		Reference		Reference		Reference	

Rainy season	1.00, 0.88-1.14	1.00	1.00, 0.87-1.14	0.94	0.97, 0.82-1.15	0.72	0.97, 0.82-1.14	0.68	1.03, 0.85-1.24	0.75	1.03, 0.85-1.24	0.76
1st ANC visit	0.89, 0.47-1.69	0.72	0.88, 0.47-1.64	0.68	0.67, 0.37-1.23	0.20	0.66, 0.36-1.21	0.18	1.32, 0.71-2.47	0.38	1.33, 0.71-2.50	0.38
Gestational age (weeks)	Not included		**0.98, 0.97-0.99**	0.0074	Not included		**0.97, 0.96-0.99**	0.0052	Not included		1.01, 0.98-1.03	0.63
**Americas and Asia, gestational age as co-variate, N=9901**												
	**Submicroscopic vs. no infection**				**Microscopic vs. no infection**				**Submicroscopic vs. microscopic infection**			
	**Base model**		**Base model + factor of interest**		**Base model**		**Base model + factor of interest**		**Base model**		**Base model + factor of interest**	
	**aOR, 95% CI**	**p-value**	**aOR, 95% CI**	**p-value**	**aOR, 95% CI**	**p-value**	**aOR, 95% CI**	**p-value**	**aOR, 95% CI**	**p-value**	**aOR, 95% CI**	**p-value**
Age (years)												
<20	**1.52, 1.05-2.19**	0.0250	**1.52, 1.05-2.19**	0.0248	**4.71, 2.16-10.24**	0.0001	**4.73, 2.17-10.28**	0.0001	**0.32, 0.14-0.74**	0.0075	0.32, 0.14-0.74	0.0073
20-24	1.34, 0.98-1.84	0.07	1.34, 0.98-1.84	0.07	**3.23, 1.54-6.77**	0.0019	**3.25, 1.55-6.81**	0.0018	**0.42, 0.19-0.91**	0.0272	0.41, 0.19-0.90	0.0259
25-29	1.12, 0.83-1.52	0.46	1.12, 0.83-1.52	0.47	**2.36, 1.14-4.90**	0.0214	**2.37, 1.14-4.92**	0.0206	0.47, 0.22-1.02	0.06	0.47, 0.22-1.02	0.06
30-34	1.12, 0.82-1.54	0.47	1.12, 0.82-1.54	0.47	1.99, 0.92-4.27	0.08	1.98, 0.92-4.27	0.08	0.56, 0.25-1.26	0.16	0.57, 0.25-1.26	0.16
35+ years	Reference		Reference		Reference		Reference		Reference		Reference	
Gravidity												
G1	**0.80, 0.64-0.99**	0.0437	**0.80, 0.64-0.99**	0.0445	1.02, 0.72-1.44	0.92	1.00, 0.71-1.42	0.98	0.78, 0.53-1.15	0.21	0.80, 0.54-1.17	0.24
G2	0.93, 0.75-1.14	0.46	0.93, 0.75-1.14	0.46	0.92, 0.64-1.30	0.63	0.91, 0.64-1.29	0.59	1.01, 0.68-1.49	0.97	1.02, 0.69-1.50	0.93
G3+	Reference		Reference		Reference		Reference		Reference		Reference	
Rainy season	0.97, 0.82-1.15	0.70	0.97, 0.82-1.15	0.70	1.25, 0.96-1.63	0.09	1.26, 0.97-1.63	0.09	0.77, 0.58-1.04	0.08	0.77, 0.58-1.03	0.08
PfPR_{2-10}	**1.06, 1.03-1.09**	0.0004	**1.06, 1.03-1.09**	0.0004	1.00, 0.96-1.04	0.97	1.00, 0.96-1.04	0.96	**1.07, 1.01-1.12**	0.0159	1.07, 1.01-1.12	0.0155
1st ANC visit	1.77, 0.44-7.04	0.42	1.77, 0.44-7.03	0.42	**10.66, 2.18-52.13**	0.0035	**10.51, 2.16-51.22**	0.0036	**0.14, 0.02-0.85**	0.0327	0.14, 0.02-0.85	0.0326
Americas vs. Asia	0.66, 0.20-2.15	0.49	0.66, 0.20-2.15	0.49	1.36, 0.34-5.49	0.67	1.32, 0.33-5.34	0.69	0.40, 0.08-2.00	0.27	0.41, 0.08-2.04	0.28
Gestational age (weeks)	Not included		1.00, 0.99-1.01	0.99	Not included		0.99, 0.97-1.01	0.16	Not included		1.01, 0.99-1.04	0.22

Table S17: Multivariable models in Africa to assess the effect of inclusion of the variable “HIV infection” into the analyses of submicroscopic and microscopic malaria infections, IPD

**Africa, high transmission level, HIV-infection as co-variate, N=3969**												
	**Submicroscopic vs. no infection**				**Microscopic vs. no infection**				**Submicroscopic vs. microscopic infection**			
	**Base model**		**Base model + factor of interest**		**Base model**		**Base model + factor of interest**		**Base model**		**Base model + factor of interest**	
	**aOR, 95% CI**	**p-value**	**aOR, 95% CI**	**p-value**	**aOR, 95% CI**	**p-value**	**aOR, 95% CI**	**p-value**	**aOR, 95% CI**	**p-value**	**aOR, 95% CI**	**p-value**
Age (years)												
<20	**2·09, 1·28-3·43**	0·0034	**2·17, 1·32-3·56**	0·0022	**4·28, 2·60-7·07**	<0·0001	**4·52, 2·74-7·49**	<0·0001	**0·49, 0·28-0·86**	0·0123	**0·48, 0·28-0·84**	0·0105
20-24	1·42, 0·92-2·20	0·12	1·46, 0·94-2·27	0·09	**2·18, 1·36-3·50**	0·0013	**2·28, 1·42-3·68**	0·0007	0·65, 0·39-1·09	0·10	0·64, 0·39-1·08	0·09
25-29	1·25, 0·81-1·94	0·31	1·28, 0·83-1·98	0·26	**1·63, 1·02-2·63**	0·0428	**1·69, 1·05-2·72**	0·0313	0·77, 0·46-1·28	0·31	0·76, 0·45-1·27	0·29
30-34	1·57, 1·00-2·46	0·05	**1·61, 1·02-2·53**	0·0400	1·45, 0·87-2·40	0·15	1·50, 0·90-2·50	0·12	1·08, 0·63-1·85	0·77	1·07, 0·63-1·83	0·81
35+ years	Reference		Reference		Reference		Reference		Reference		Reference	
Gravidity												
G1	**0·67, 0·47-0·95**	0·0256	**0·67, 0·47-0·95**	0·0265	**1·80, 1·32-2·44**	0·0002	**1·81, 1·33-2·45**	0·0002	**0·37, 0·26-0·52**	<0·0001	**0·37, 0·26-0·52**	<0·0001
G2	0.76, 0.56-1.03	0.08	0.76, 0.56-1.03	0.08	1·14, 0·86-1·51	0·37	1·14, 0·86-1·51	0·35	**0·66, 0·49-0·89**	0·0069	**0·66, 0·49-0·89**	0·0066
G3+	Reference		Reference		Reference		Reference		Reference		Reference	
Rainy season	**0·70, 0·56-0·87**	0·0012	**0·70, 0·56-0·87**	0·0012	0·92, 0·78-1·09	0·35	0·92, 0·78-1·09	0·35	**0·75, 0·61-0·93**	0·0082	**0·75, 0·61-0·93**	0·0084
1st ANC visit	**0.30, 0.16-0.55**	0.0001	**0.30, 0.16-0.58**	0.0003	**0·42, 0·19-0·96**	0·0392	0·44, 0·19-1·03	0·06	0·79, 0·51-1·24	0·31	0·78, 0·50-1·24	0·30
HIV infection	Not included		1·52, 0·97-2·38	0·07	Not included		**1·90, 1·22-2·96**	0·0047	Not included		0·80, 0·50-1·27	0·34
**Africa, moderate-to-low transmission level, HIV-infection as co-variate, N=7569**												
	**Submicroscopic vs. no infection**				**Microscopic vs. no infection**				**Submicroscopic vs. microscopic infection**			
	**Base model**		**Base model + factor of interest**		**Base model**		**Base model + factor of interest**		**Base model**		**Base model + factor of interest**	
	**aOR, 95% CI**	**p-value**	**aOR, 95% CI**	**p-value**	**aOR, 95% CI**	**p-value**	**aOR, 95% CI**	**p-value**	**aOR, 95% CI**	**p-value**	**aOR, 95% CI**	**p-value**
Age (years)												
<20	**2·24, 1·62-3·09**	<0·0001	**2·23, 1·62-3·08**	<0·0001	**4·41, 2·66-7·32**	<0·0001	**4·50, 2·71-7·48**	<0·0001	**0·51, 0·29-0·88**	0·0168	**0·50, 0·28-0·87**	0·0135
20-24	**1·36, 1·02-1·82**	0·0356	**1·36, 1·02-1·81**	0·0366	**2·26, 1·40-3·64**	0·0009	**2·29, 1·42-3·69**	0·0007	0·60, 0·36-1·02	0·06	0·59, 0·35-1·00	0·05
25-29	1·17, 0·89-1·53	0·26	1·17, 0·89-1·53	0·26	1·49, 0·93-2·39	0·10	1·49, 0·93-2·38	0·10	0·78, 0·47-1.31	0·35	0·79, 0·47-1.31	0·36
30-34	1·12, 0·84-1·50	0·44	1·12, 0·84-1·50	0·44	1·18, 0·71-1·97	0·52	1·17, 0·70-1·95	0·56	0·95, 0·55-1·65	0·85	0·96, 0·55-1·68	0·89
35+ years	Reference		Reference		Reference		Reference		Reference		Reference	
Gravidity												
G1	1·16, 0·93-1·45	0·18	1·16, 0·93-1·45	0·19	**2·10, 1·57-2·80**	<0·0001	**2·12, 1·59-2·84**	<0·0001	**0·55, 0·40-0·77**	0·0004	**0·55, 0·39-0·76**	0·0003
G2	1·07, 0·88-1·29	0·52	1·06, 0·88-1·29	0·53	1·21, 0·92-1·60	0·17	1·23, 0·93-1·62	0·15	0·88, 0·65-1·19	0·40	0·87, 0·64-1·18	0·37
G3+	Reference		Reference		Reference		Reference		Reference		Reference	
Rainy season	0·94, 0·82-1·06	0·31	0·94, 0·82-1·06	0·31	0·93, 0·79-1·09	0·37	0·94, 0·79-1·10	0·42	1·01, 0·84-1.21	0·93	1·00, 0·83-1.20	0·99

1st ANC visit	0·80, 0·41-1·56	0·52	0·80, 0·41-1·56	0·51	0·63, 0·34-1·18	0·15	0·70, 0·36-1·34	0·28	1·26, 0·67-2·40	0·47	1·14, 0·56-2·32	0·71
HIV infection	Not included		0·95, 0·64-1·43	0·82	Not included		**1·78, 1·07-2·95**	0·0262	Not included		**0·54, 0·30-0·97**	0·0396

Table S18: Multivariable models in Africa to assess the effect of inclusion of the variable "Antimalarial use" into the analyses of submicroscopic and microscopic malaria infections, IPD

**Africa, high transmission level, antimalarial use as co-variate, N=1347**												
	**Submicroscopic vs. no infection**				**Microscopic vs. no infection**				**Submicroscopic vs. microscopic infection**			
	**Base model**		**Base model + factor of interest**		**Base model**		**Base model + factor of interest**		**Base model**		**Base model + factor of interest**	
	**aOR, 95% CI**	**p-value**	**aOR, 95% CI**	**p-value**	**aOR, 95% CI**	**p-value**	**aOR, 95% CI**	**p-value**	**aOR, 95% CI**	**p-value**	**aOR, 95% CI**	**p-value**
Age (years)												
<20	1·52, 0·81-2·84	0·19	1·55, 0·82-2·95	0·18	**3·68, 2·04-6·62**	<0·0001	**3·47, 1·92-6·28**	<0·0001	**0·42, 0·24-0·72**	0·0015	**0·45, 0·26-0·79**	0·0049
20-29	**1·44, 1·01-2·05**	0·0434	**1·43, 1·00-2·05**	0·0474	**2·16, 1·47-3·17**	0·0001	**2·08, 1·41-3·07**	0·0002	**0·67, 0·47-0·96**	0·0282	**0·69, 0·48-0·99**	0·0452
30+ years	Reference		Reference		Reference		Reference		Reference		Reference	
Gravidity												
G1	0·67, 0·40-1·12	0·13	0·65, 0·39-1·10	0·11	**1·97, 1·24-3·13**	0·0039	**1·98, 1·24-3·17**	0·0042	**0·34, 0·22-0·52**	<0·0001	**0·33, 0·21-0·51**	<0·0001
G2	0.68, 0.45-1.02	0.06	**0.65, 0.43-0.99**	0.0430	1·34, 0·92-2·00	0·13	1·30, 0·88-1·94	0·19	**0·50, 0·35-0·72**	0·0002	**0·50, 0·34-0·71**	0·0002
G3+	Reference		Reference		Reference		Reference		Reference		Reference	
Rainy season	**0·60, 0·41-0·89**	0·0103	**0·55, 0·36-0·88**	0·0043	0·69, 0·47-1·03	0·07	0·68, 0·46-1·00	0·05	0·86, 0·64-1·16	0·33	0·78, 0·57-1·08	0·13
Antimalarial use	Not included		**0·56, 0·36-0·88**	0·0114	Not included		**0·27, 0·18-0·40**	<0·0001	Not included		**2·08, 1·42-3·05**	0·0002

Notes: Because of non-convergence of the model, age was collapsed from 5 into 3 categories. The variable "First ANC visit" was not included; all involved studies included women at any ANC visit, or it was unknown.

**Africa, moderate-to-low transmission level, antimalarial use as co-variate, N=1479**												
	**Submicroscopic vs. no infection**				**Microscopic vs. no infection**				**Submicroscopic vs. microscopic infection**			
	**Base model**		**Base model + factor of interest**		**Base model**		**Base model + factor of interest**		**Base model**		**Base model + factor of interest**	
	**aOR, 95% CI**	**p-value**	**aOR, 95% CI**	**p-value**	**aOR, 95% CI**	**p-value**	**aOR, 95% CI**	**p-value**	**aOR, 95% CI**	**p-value**	**aOR, 95% CI**	**p-value**
Age (years)												
<20	**2.57,1.53-4.31**	0.0004	**2.44, 1.45-4.11**	0.0008	1.94,0.87-4.33	0.11	1.95, 0.87-4.36	0.11	1.32, 0.53-3.32	0.55	1.26, 0.50-3.16	0.63
20-29	1.14,0.79-1.64	0.49	1.12, 0.78-1.62	0.54	1.44,0.72-2.88	0.30	1.44, 0.72-2.87	0.31	0.79, 0.37-1.68	0.54	0.78, 0.37-1.67	0.52
30+ years			Reference				Reference		Reference		Reference	
Primigravidae	0.77,0.54-1.11	0.17	0.79, 0.55-1.14	0.20	1.54,0.93-2.56	0.09	1.54, 0.93-2.54	0.10	**0.50, 0.28-0.90**	0.0210	**0.51, 0.29-0.93**	**0.0266**
Rainy season	**1.44,1.05-1.98**	0.0227	**1.44, 1.05-1.97**	0.0245	**0.61,0.40-0.93**	0.0210	**0.61, 0.40-0.93**	0.0227	**2.37, 1.44-3.93**	0.0008	**2.35, 1.42-3.89**	**0.0009**
Antimalarial use	Not included		**0.45, 0.27-0.76**	0.0027	Not included		1.12, 0.68-1.86	0.65	Not included		**0.40, 0.20-0.81**	**0.0105**

Notes: Because of non-convergence of the models, age was collapsed from 5 into 3 categories and gravidity into two (primigravidae versus multigravidae). The variable "First ANC visit" was not included; all involved studies included women at any ANC visit, or it was unknown.

**Americas and Asia, antimalarial use as co-variate, N=5407**		
**Submicroscopic vs. no infection**	**Microscopic vs. no infection**	**Submicroscopic vs. microscopic infection**

	**Base model**		**Base model + factor of interest**		**Base model**		**Base model + factor of interest**		**Base model**		**Base model + factor of interest**	
	**aOR, 95% CI**	**p-value**	**aOR, 95% CI**	**p-value**	**aOR, 95% CI**	**p-value**	**aOR, 95% CI**	**p-value**	**aOR, 95% CI**	**p-value**	**aOR, 95% CI**	**p-value**
Age (years)												
<20	1.24, 0.76-2.03	0.39	1.24, 0.76-2.04	0.38	**5.83, 2.28-14.90**	0.0002	**5.86, 2.29-14.97**	0.0002	**0.21, 0.08-0.58**	0.0026	**0.21, 0.08-0.58**	0.0026
20-24	1.18, 0.76-1.84	0.45	1.19, 0.76-1.84	0.45	**3.52, 1.42-8.70**	0.0064	**3.51, 1.42-8.67**	0.0065	**0.34, 0.13-0.88**	0.0258	**0.34, 0.13-0.88**	0.0263
25-29	0.84, 0.54-1.29	0.42	0.84, 0.54-1.29	0.42	**2.91, 1.20-7.07**	0.0185	**2.90, 1.20-7.05**	0.0186	**0.29, 0.11-0.74**	0.0094	**0.29, 0.11-0.74**	0.0095
30-34	0.92, 0.58-1.46	0.73	0.92, 0.58-1.45	0.71	**2.56, 1.02-6.44**	0.0460	**2.54, 1.01-6.38**	0.0480	**0.36, 0.14-0.96**	0.0412	**0.36, 0.14-0.96**	0.0419
35+ years	Reference		Reference		Reference		Reference		Reference		Reference	
Gravidity												
G1	0.88, 0.65-1.18	0.38	0.87, 0.65-1.17	0.37	1.08, 0.72-1.61	0.72	1.07, 0.72-1.59	0.75	0.81, 0.52-1.28	0.37	0.82, 0.52-1.29	0.39
G2	1.15, 0.86-1.53	0.34	1.15, 0.86-1.53	0.35	1.11, 0.74-1.67	0.61	1.11, 0.74-1.66	0.63	1.03, 0.65-1.63	0.89	1.04, 0.66-1.64	0.88
G3+	Reference		Reference		Reference		Reference		Reference		Reference	
Rainy season	1.10, 0.88-1.36	0.40	1.10, 0.89-1.36	0.39	1.12, 0.84-1.49	0.43	1.12, 0.85-1.49	0.42	0.98, 0.71-1.35	0.90	0.98, 0.71-1.35	0.89
*Pf*PR$_{2-10}$	**0.60, 0.43-0.85**	0.0036	**0.60, 0.43-0.85**	0.0036	**0.71, 0.51-1.00**	0.0473	**0.71, 0.51-1.00**	0.0470	0.85, 0.53-1.36	0.50	0.85, 0.53-1.36	0.50
1st ANC visit	2.45, 0.48-12.49	0.28	2.41, 0.48-12.24	0.29	5.72, 0.72-45.28	0.10	5.56, 0.71-43.65	0.10	0.43, 0.04-4.68	0.49	0.43, 0.04-4.77	0.49
Americas vs. Asia	0.60, 0.14-2.54	0.48	0.58, 0.14-2.47	0.46	0.53, 0.08-3.43	0.51	0.51, 0.08-3.27	0.48	1.12, 0.13-9.81	0.92	1.14, 0.13-10.04	0.90
Antimalarial use	Not included		1.19, 0.81-1.74	0.38	Not included		1.35, 0.87-2.08	0.18	Not included		0.88, 0.53-1.47	0.62

Table S19: Delivery: Multivariable analyses of factors associated with submicroscopic and microscopic malaria infections peripheral blood, IPD: Base model and variables of interest with limited sample size

		**Africa, high transmission*, delivery, maternal peripheral blood**							
		**Any malaria infection vs. no malaria**		**Microscopic vs. no malaria**		**Submicroscopic vs. no malaria**		**Risk of submicroscopic infections among NAAT-positive infections**	
		**Base model** N=4599, 92.4% of available data, 9 sublocations†							
		**aOR, 95% CI**	**p-value**	**aOR, 95% CI**	**p-value**	**aOR, 95% CI**	**p-value**	**aOR, 95% CI**	**p-value**
Age (years)									
<20		**1.33, 1.01-1.76**	0.0446	**1.63, 1.09-2.46**	0.0184	1.15, 0.83-1.61	0.41	0.70, 0.44-1.12	0.14
20-29		1.10, 0.90-1.35	0.35	1.29, 0.94-1.78	0.11	1.03, 0.82-1.30	0.79	0.80, 0.56-1.13	0.21
30+ years		Reference		Reference		Reference		Reference	
Gravidity									
G1		**1.30, 1.04-1.62**	0.0211	**1.82, 1.32-2.50**	0.0002	1.04, 0.80-1.36	0.75	**0.58, 0.40-0.83**	0.0031
G2		1.22, 0.99-1.51	0.06	**1.38, 1.02-1.88**	0.0390	1.18, 0.93-1.51	0.17	0.86, 0.61-1.22	0.40
G3+		Reference		Reference		Reference		Reference	
PfPR_{2-10}		**0.97, 0.95-0.99**	0.0030	0.99, 0.96-1.02	0.54	**0.96, 0.94-0.98**	0.0003	**0.96, 0.93-1.00**	0.0454
Rainy vs. dry season		**2.42, 2.06-2.84**	<0.0001	**4.19, 3.25-5.40**	<0.0001	**1.73, 1.43-2.08**	<0.0001	**0.41, 0.31-0.55**	<0.0001
Survey vs. cohort/trial		0.73, 0.02-25.26	0.86	0.42, 0.01-12.11	0.61	1.01, 0.03-38.01	1.00	3.87, 0.83-17.99	0.08
	**Available data (%)**	**Base model with additional variables of interest with limited sample size**							
Preterm delivery	77.1	1.08, 0.85-1.37	0.54	0.96, 0.69-1.32	0.78	1.17, 0.88-1.56	0.29	1.22, 0.85-1.76	0.28
HIV-infection‡	30.0	0.67, 0.22-2.02	0.48	**0.28, 0.14-0.58**	0.0007	0.70, 0.24-2.10	0.53	2.49, 0.70-8.84	0.16
Rural setting‡	28.6	1.05, 0.80-1.37	0.74	1.13, 0.77-1.65	0.54	1.05, 0.77-1.43	0.75	0.92, 0.61-1.37	0.67
Antimalarial use	87.2	**0.75, 0.57-0.98**	0.0349	**0.68, 0.48-0.98**	0.0370	0.79, 0.57-1.10	0.16	1.16, 0.77-1.75	0.47
ITN use likely	64.7	0.89, 0.68-1.15	0.37	**0.55, 0.40-0.75**	0.0002	1.27, 0.90-1.78	0.18	**2.23, 1.50-3.33**	0.0001
Any net use‡	34.7	0.88, 0.67-1.15	0.35	**0.66, 0.47-0.92**	0.0141	1.27, 0.92-1.75	0.15	**1.97, 1.27-3.08**	0.0027
		**Africa, moderate-to-low transmission*, delivery, maternal peripheral blood**							
		**Any malaria infection vs. no malaria**		**Microscopic vs. no malaria**		**Submicroscopic vs. no malaria**		**Risk of submicroscopic infections among NAAT-positive infections**	
		**Base model** N=9981, 93.0% of available data, 33 sublocations†							
		**aOR, 95% CI**	**p-value**	**aOR, 95% CI**	**p-value**	**aOR, 95% CI**	**p-value**	**aOR, 95% CI**	**p-value**
Age (years)									
<20		**1.28, 1.01-1.62**	0.0421	**1.71, 1.15-2.55**	0.0084	1.15, 0.88-1.50	0.32	0.67, 0.43-1.04	0.08
20-29		0.99, 0.84-1.17	0.91	1.14, 0.84-1.54	0.41	0.95, 0.79-1.15	0.60	0.84, 0.60-1.17	0.30
30+ years		Reference		Reference		Reference		Reference	
Gravidity									
G1		**1.26, 1.03-1.54**	0.0256	**1.89, 1.38-2.59**	<0.0001	1.01, 0.80-1.28	0.93	**0.53, 0.37-0.76**	0.0005
G2		1.18, 0.99-1.41	0.06	1.22, 0.91-1.64	0.19	1.12, 0.92-1.37	0.26	0.92, 0.66-1.27	0.61
G3+		Reference		Reference		Reference		Reference	

PfPR_{2-10}		1.01, 0.99-1.02	0.52	1.01, 0.99-1.04	0.31	1.00, 0.98-1.02	0.89	0.99, 0.96-1.02	0.40
Rainy vs. dry season		**1.22, 1.08-1.38**	0.0018	**1.49, 1.20-1.85**	0.0003	1.13, 0.98-1.29	0.10	**0.75, 0.59-0.96**	0.0219
Survey vs. cohort/trial		1.40, 0.64-3.07	0.39	1.67, 0.70-4.00	0.25	1.42, 0.62-3.25	0.41	0.85, 0.42-1.72	0.65
	**Available data (%)**	**Base model with additional variables of interest with limited sample size**							
Preterm delivery	88.3	**1.24, 1.05-1.47**	0.0128	**1.68, 1.27-2.21**	0.0002	1.11 0.91-1.34	0.30	**0.66, 0.48-0.90**	0.0083
HIV-infection	83.6	**0.58, 0.41-0.82**	0.0022	0.85, 0.52-1.38	0.51	**0.48, 0.32-0.72**	0.0005	0.56, 0.32-1.00	0.05
Rural setting	40.7	1.36, 0.58-3.23	0.48	1.40, 0.39-5.06	0.60	1.31, 0.55-3.09	0.54	1.06, 0.33-3.46	0.92
Antimalarial use	88.2	**0.63, 0.53-0.75**	<0.0001	**0.65, 0.48-0.90**	0.0080	**0.62, 0.51-0.76**	<0.0001	0.95, 0.67-1.35	0.77
ITN use likely	79.7	**0.58, 0.44-0.77**	0.0002	0.65, 0.41-1.04	0.07	**0.56, 0.40-0.77**	0.0005	0.86, 0.50-1.48	0.59
Any net use	53.7	**0.70, 0.55-0.90**	0.0051	**0.64, 0.42-0.96**	0.0328	**0.73, 0.55-0.98**	0.0364	1.15, 0.71-1.84	0.57
**Americas and Asia, delivery, maternal peripheral blood**									
		**Any malaria infection vs. no malaria**		**Microscopic vs. no malaria**		**Submicroscopic vs. no malaria**		**Risk of submicroscopic infections among NAAT-positive infections**	
**Base model** N=5891, 96.5% of available data, 16 sublocations†									
		**aOR, 95% CI**	**p-value**	**aOR, 95% CI**	**p-value**	**aOR, 95% CI**	**p-value**	**aOR, 95% CI**	**p-value**
Age (years)									
<20		**2.08, 1.44-3.01**	<0.0001	**3.13, 1.40-6.99**	0.0055	**1.94, 1.29-2.90**	0.0013	0.62, 0.26-1.49	0.28
20-29		**1.47, 1.12-1.92**	0.0059	**2.06, 1.13-3.77**	0.0188	**1.35, 1.01-1.82**	0.0454	0.66, 0.34-1.26	0.21
30+ years		Reference		Reference		Reference		Reference	
Gravidity									
G1		**0.71, 0.55-0.93**	0.0111	**0.54, 0.31-0.94**	0.0303	0.75, 0.56-1.00	0.05	1.38, 0.75-2.52	0.30
G2		0.98, 0.75-1.27	0.85	1.11, 0.66-1.87	0.68	0.93, 0.69-1.24	0.62	0.83, 0.47-1.48	0.53
G3+		Reference		Reference		Reference		Reference	
PfPR_{2-10}		0.98, 0.96-1.00	0.10	0.98, 0.94-1.02	0.25	0.98, 0.96-1.01	0.21	1.01, 0.96-1.05	0.75
Rainy vs. dry season		1.06, 0.86-1.32	0.57	**1.61, 1.03-2.52**	0.0355	0.95, 0.75-1.20	0.65	**0.59, 0.36-0.96**	0.0321
Survey vs. cohort/trial		**0.14, 0.03-0.73**	0.0194						
Americas vs. Asia		0.63, 0.23-1.70	0.36	**0.04, 0.01-0.30**	0.0019	0.44, 0.15-1.26	0.13	**11.29, 1.43-89.00**	0.0214
	**Available data (%)**	**Base model with additional variables of interest with limited sample size**							
Preterm delivery	83.7	**1.47, 1.14-1.91**	0.0034	**2.25, 1.33-3.81**	0.0025	**1.36, 1.02-1.80**	0.0356	0.60, 0.34-1.07	0.08
Rural setting	69.8	1.36, 0.98-1.88	0.07	**1.82, 1.03-3.23**	0.0400	1.18, 0.80-1.73	0.41	0.65, 0.33-1.27	0.21
Antimalarial use	87.7	1.14, 0.81-1.58	0.45	1.98, 0.92-4.26	0.08	1.02, 0.71-1.48	0.91	0.52, 0.22-1.18	0.12
Any net use	71.2	1.13, 0.85-1.51	0.39	1.25, 0.70-2.23	0.44	1.11, 0.81-1.53	0.50	0.89, 0.48-1.66	0.71

aOR, adjusted Odds ratio. G1, primigravidae. G2, secundigravidae. G3+, multigravidae (excluding secundigravidae). ITN, insecticide treated net. IRS, indoor residual spraying. Available data: number of participants with an outcome on submicroscopic, microscopic and no malaria. Note: there was insufficient information about IRS to allow useful models. There was insufficient information on HIV in Asia/Americas. *High transmission: *Pf*PR_{2-10} ≥35%. Moderate and low transmission: *Pf*PR_{2-10} <35%. †Available data: Africa, high transmission N=4976, 10 sublocations (microscopic 511, submicroscopic 826, and no malaria infections 3639 observations). Africa, moderate-to-low transmission N=10,737 (microscopic 505, submicroscopic 1232, no malaria infection 9000), 36 sublocations. Asia/Americas 6,102 (microscopic 111, submicroscopic 446 and no malaria infection 5545), 16 sublocations. ‡ Africa high transmission, subgroup analysis: HIV infection: model with HIV infection only, non-convergence of adjusted models, very low numbers of HIV-infected women with malaria. Setting, subgroup analysis: survey and *Pf*PR_{2-10} removed because of non-convergence. Only 1 cohort study in rural area. Any net use, subgroup analysis: only univariate analysis because of non-convergence.

Table S20: Delivery: Multivariable analyses of factors associated with placental submicroscopic and microscopic malaria infections, IPD: Base model and variables of interest with limited sample size

**Africa, high transmission level*, delivery, placental blood**									
		**Any malaria infection vs. no malaria**		**Microscopic vs. no malaria**		**Submicroscopic vs. no malaria**		**Risk of submicroscopic infections among NAAT-positive infections**	
		**Base model** N=5028, 93.3% of available data, 10 sublocations†							
		**aOR, 95% CI**	**p-value**	**aOR, 95% CI**	**p-value**	**aOR, 95% CI**	**p-value**	**aOR, 95% CI**	**p-value**
Age (years)									
<20		**1.42, 1.09-1.86**	0.0091	**1.80, 1.26-2.57**	0.0012	1.10, 0.79-1.54	0.56	**0.61, 0.40-0.94**	0.0264
20-29		1.17, 0.97-1.40	0.10	**1.37, 1.06-1.78**	0.0170	1.05, 0.85-1.30	0.67	0.76, 0.57-1.03	0.08
30+ years		Reference		Reference		Reference		Reference	
Gravidity		**1.40, 1.14-1.73**	0.0016						
G1		0.99, 0.82-1.21	0.95	**2.03, 1.54-2.66**	<0.0001	0.99, 0.76-1.29	0.96	**0.49, 0.35-0.68**	<0.0001
G2		Reference		**1.27, 0.98-1.65**	0.07	0.81, 0.64-1.04	0.10	**0.64, 0.47-0.88**	0.0057
G3+		**0.98, 0.96-1.00**	0.0159	Reference		Reference		Reference	
*Pf*PR$_{2-10}$		**3.04, 2.57-3.58**	<0.0001	1.00, 0.97-1.03	0.93	**0.97, 0.94-0.99**	0.0096	0.97, 0.93-1.01	0.11
Rainy vs. dry season		2.56, 0.92-7.07	0.07	**4.18, 3.31-5.29**	<0.0001	**2.33, 1.89-2.86**	<0.0001	**0.56, 0.42-0.74**	<0.0001
Survey vs. cohort/trial		**1.42, 1.09-1.86**	0.0091	1.57, 0.25-9.97	0.63	2.47, 0.83-7.33	0.10	1.57, 0.21-11.90	0.66
	**Available data (%)**	**Base model with additional variables of interest with limited sample size**							
Preterm delivery	78.3	1.09, 0.88-1.37	0.43	0.15, 0.88-1.49	0.31	1.00, 0.74-1.34	0.99	0.87, 0.63-1.21	0.41
HIV-infection‡	37.4	**0.30, 0.21-0.45**	<0.0001	0.78, 0.21-2.96	0.71	0.59, 0.21-1.71	0.33	0.76 0.22-2.59	0.66
Rural setting‡	35.3	0.88, 0.67-1.17	0.38	0.88, 0.64-1.22	0.45	0.90, 0.64-1.27	0.5	1.02, 0.71-1.46	0.92
Antimalarial use	88.7	**0.60, 0.44-0.84**	0.0024	**0.50, 0.34-0.75**	0.0007	0.71, 0.48-1.05	0.09	1.41, 0.89-2.22	0.14
ITN use likely	69.0	0.83, 0.67-1.02	0.07	**0.67, 0.52-0.87**	0.0021	**1.14, 0.85-1.52**	0.39	**1.69, 1.20-2.39**	0.0029
Any net use	42.1	0.86, 0.69-1.07	0.18	No convergence		No convergence		No convergence	
**Africa, moderate-to-low transmission level*, delivery, placental blood**									
		**Any malaria infection vs. no malaria**		**Microscopic vs. no malaria**		**Submicroscopic vs. no malaria**		**Risk of submicroscopic infections among NAAT-positive infections**	
		**Base model** N=7664, 95.2% of available data, 31 sublocations†							
		**aOR, 95% CI**	**p-value**	**aOR, 95% CI**	**p-value**	**aOR, 95% CI**	**p-value**	**aOR, 95% CI**	**p-value**
Age (years)									
<20		**1.48, 1.12-1.95**	0.0053	**2.12, 1.34-3.34**	0.0013	1.27, 0.93-1.74	0.14	0.60, 0.36-1.01	0.05
20-29		1.09, 0.89-1.34	0.39	1.34, 0.94-1.91	0.11	1.02, 0.81-1.28	0.90	0.76, 0.51-1.13	0.17
30+ years		Reference		Reference		Reference		Reference	
Gravidity									
G1		**1.39, 1.10-1.75**	0.0053	**1.80, 1.26-2.57**	0.0012	1.20, 0.92-1.58	0.18	0.67, 0.45-1.00	0.05
G2		**1.25, 1.02-1.53**	0.0294	1.31, 0.95-1.82	0.10	1.20, 0.95-1.51	0.12	0.91, 0.63-1.32	0.63
G3+		Reference		Reference		Reference		Reference	

PfPR_{2-10}		1.02, 1.00-1.04	0.07	**1.03, 1.00-1.06**	0.0454	1.01, 0.99-1.04	0.28	0.98, 0.95-1.01	0.29
Rainy vs. dry season		**1.27, 1.10-1.45**	0.0008	1.11, 0.89-1.40	0.35	**1.34, 1.14-1.57**	0.0003	1.21, 0.93-1.56	0.16
Survey vs. cohort/trial		1.37, 0.57-3.30	0.48	0.90, 0.32-2.56	0.84	1.65, 0.66-4.16	0.29	1.84, 0.80-4.23	0.15
	**Available data (%)**	**Base model with additional variables of interest with limited sample size**							
Preterm delivery	92.5	**1.56, 1.29-1.88**	<0.0001	**1.85, 1.37-2.49**	0.0001	**1.45, 1.16-1.80**	0.0009	0.78, 0.56-1.09	0.15
HIV-infection	85.0	**0.60, 0.40-0.90**	0.0135	0.81, 0.48-1.37	0.43	0.52, 0.32-0.83	0.0060	0.64, 0.36-1.15	0.14
Rural setting	48.9	1.34, 0.61-2.93	0.46	1.13, 0.37-3.45	0.83	1.52, 0.74-3.12	0.25	1.35, 0.67-2.71	0.40
Antimalarial use	92.9	**0.57, 0.46-0.69**	<0.0001	**0.65, 0.47-0.89**	0.0073	**0.53, 0.42-0.66**	<0.0001	0.82, 0.57-1.18	0.28
ITN use likely	88.4	**0.70, 0.52-0.95**	0.0211	0.72, 0.37-1.41	0.33	0.69, 0.49-0.95	0.0244	0.95, 0.46-1.98	0.90
Any net use	47.5	0.89, 0.67-1.16	0.38	0.73, 0.41-1.30	0.28	0.95, 0.70-1.27	0.71	1.30, 0.69-2.45	0.41
**Americas and Asia, delivery, placental blood**									
		**Any malaria infection vs. no malaria**		**Microscopic vs. no malaria**		**Submicroscopic vs. no malaria**		**Risk of submicroscopic infections among NAAT-positive infections**	
		**Base model** N=4888, 97.7% of available data, 18 sublocations†							
		**aOR, 95% CI**	**p-value**	**aOR, 95% CI**	**p-value**	**aOR, 95% CI**	**p-value**	**aOR, 95% CI**	**p-value**
Age (years)									
<20		1.11, 0.66-1.84	0.70	0.66, 0.27-1.60	0.35	1.36, 0.76-2.44	0.31	2.06, 0.74-5.74	0.17
20-29		1.22, 0.85-1.75	0.29	0.90, 0.48-1.69	0.74	1.36, 0.89-2.06	0.15	1.52, 0.73-3.15	0.26
30+ years		Reference		Reference		Reference		Reference	
Gravidity									
G1		1.12, 0.80-1.58	0.51	1.61, 0.87-2.99	0.13	0.98, 0.66-1.46	0.94	0.61, 0.30-1.23	0.17
G2		1.29, 0.91-1.82	0.15	1.81, 0.97-3.38	0.06	1.14, 0.77-1.69	0.52	0.63, 0.31-1.28	0.20
G3+		Reference		Reference		Reference		Reference	
PfPR_{2-10}		1.00, 0.97-1.02	0.88	0.99, 0.94-1.03	0.56	1.00, 0.97-1.04	0.82	4.30, 0.94-19.75	0.06
Rainy vs. dry season		**1.33, 1.00-1.76**	0.0500	**1.80, 1.11-2.92**	0.0172	1.16, 0.84-1.61	0.36	0.64, 0.37-1.12	0.12
Survey vs. cohort/trial		0.52, 0.16-1.68	0.28	0.71, 0.07-7.05	0.77	0.54, 0.16-1.90	0.34	0.23, 0.05-1.09	0.06
Americas vs. Asia		0.45, 0.13-1.50	0.19	0.15, 0.01-2.16	0.16	0.57, 0.16-2.05	0.39	0.69, 0.07-6.61	0.75
	**Available data (%)**	**Base model with additional variables of interest with limited sample size**							
Preterm delivery	84.0	**1.62, 1.17-2.25**	0.0038	**2.14, 1.24-3.68**	0.0059	1.44, 0.99-2.10	0.06	0.67, 0.36-1.24	0.21
Rural setting	88.6	1.28, 0.91-1.79	0.16	**2.07, 1.14-3.77**	0.0168	1.00, 0.67-1.51	0.99	**0.48, 0.24-0.99**	0.0470
Antimalarial use	92.6	1.27, 0.84-1.92	0.26	**3.34, 1.39-8.01**	0.0069	0.96, 0.60-1.54	0.87	**0.29, 0.11-0.76**	0.0119
Any net use	89.9	1.20, 0.87-1.64	0.27	0.63, 0.36-1.10	0.11	**1.50, 1.05-2.13**	0.0264	**2.39, 1.27-4.48**	0.0069

aOR, adjusted Odds ratio. G1, primigravidae. G2, secundigravidae. G3+, multigravidae (excluding secundigravidae). ITN, insecticide treated net. IRS, indoor residual spraying. Available data: number of participants with an outcome on submicroscopic, microscopic and no malaria. Note: there was insufficient information about IRS to allow useful models. There was Insufficient information on HIV in Asia/Americas. *High transmission: *Pf*PR_{2-10} ≥35%, moderate-to-low transmission *Pf*PR_{2-10} <35%. †Available data: Africa, high transmission N=5391, 11 sublocations (microscopic 750, submicroscopic 738 and no malaria infection 3903 observations). Africa, moderate-to-low transmission N=8048 (microscopic 440, submicroscopic 877 and no malaria infection 6731), 33 sublocations. Asia/Americas N=5001 (microscopic 97, submicroscopic 235, and no malaria infection 4669), 18 sublocations. ‡ Africa high transmission; HIV subgroup analyses: only age and gravidity included because of non-convergence. Setting subgroup analysis: *Pf*PR_{2-10} removed because of non-convergence.

Table S21: Microscopy and fever results at second scheduled study visit in pregnancy after submicroscopic malaria at enrolment

			**Submicroscopic malaria at 2nd scheduled study visit**				**Microscopic malaria at 2nd scheduled study visit**				**No malaria at 2nd scheduled study visit**			
	**N sub-studies**	**N women**	**Pooled estimate*, %, (95% CI)**	**I^2, %**	**Median (%), range**	**Median days difference, range, n**	**Pooled estimate*, %, (95% CI)**	**I^2, %**	**Median (%), range**	**Median days difference, range, n**	**Pooled estimate* %, (95% CI)**	**I^2, %**	**Median (%), range**	**Median days difference, range, n**
Overall	20	1079	18.0 (10.6-26.6)	88.9	15.8, 0.0-56.0	49, 14-125, 277†	6.2 (2.4-11.1)	82.1	6.9, 0.0-37.7	28, 14-101, 126†	74.4 (60.9-86.1)	94.7	77.5, 13.2-100	70, 15-1147, 676†
Asia/Americas	5	70	0.0 (0.0-2.5)	0.0	0.0, 0.0-11.1	27, 1	0.0 (0.0-1.3)	0.0	0.0, 0.0-0.0	n=0	100 (97.5-100)	0.0	100, 88.9-100	32, 22-147, 69
Africa	15	1009	24.0 (15.8-33.1)	89.0	18.5, 7.7-56.0	50, 14-125, 276	8.8 (4.3-14.6)	84.7	8.5, 0.0-37.7	28, 14-101, 126	65.0 (50.5-78.3)	95.0	73.5, 13.2-92.3	71, 15-119, 607
			**Submicroscopic malaria and fever at 2nd scheduled study visit**				**Microscopic malaria and fever at 2nd scheduled study visit**				**No malaria and fever present at 2nd scheduled study visit**			
Overall	7	885	1.1 (0.0-9.1)	57.7	4.1, 0.0-50.0	30, 15-93, 17†	2.2, 0-13.3	44.1	0.0, 0.0-20.0	24, 15-27, 8†	3.1, 0.0-8.9	69.0	9.5, 1.7-50.0	42, 23-98, 17†
Asia/Americas	2	70	0.0, (0.0-98.0)		0.0				0.0		1.4, 0.0-6.9		5.8, 1.7-10.0	44, 32-55, 2
Africa	5	815	3.8, (0.0-13.1)	65.9	5.1, 0.0-50.0	30, 15-93, 17	2.2, 0-13.3	44.1	0.0, 0.0-20.0	24, 15-27, 8	3.8, 0-12.6	77.4	9.5, 1.9-50.0	42, 23-98, 17

Fever was defined as documented fever (<37.5 °C) or a history of fever in the past 1-7 days as per the definition used in the source studies.
*Meta-prop procedure Stata
†Comparing the difference in days using multinomial model (GSEM) with no malaria as reference: p=0.6352 comparing submicroscopic at 2nd visit vs. none, and p=0.3492 comparing microscopic vs. none for overall model. Among participants with fever at 2nd study visit: p=0.1826 and p=0.2943 comparing submicroscopic at 2nd visit vs. none, and microscopic vs. none for overall model, respectively. Comparing difference in days for submicroscopic vs. microscopic: p=0.1869

Table S22: Sensitivity analysis: Comparing IPD and aggregated data for study outcomes

	**Studies contributing individual participant data**					**Studies contributing extracted data**					**Meta-regression**	**Median test†**
	**N sub-studies**	**N study participants**	**Pooled estimate* (%, 95% CI)**	**I^2, %**	**Median, range**	**N sub-studies**	**N study participants**	**Pooled estimate* (%, 95% CI)**	**I^2, %**	**Median**	**p-value**	**p-value**
**Pregnancy**												
Submicroscopic malaria	56	21,624	15.9 (12.9-19.1)	97.8	16.7, 0.0-55.9	10	6028	8.4 (6.0-11.0)	90.7	8.2, 2.0-28.2	0.0708	0.0248
Microscopic malaria	56	21,218	10.1 (6.8-14.0)	98.9	8.3, 0.0-50.6	10	6015	7.9 (4.3-12.3)	96.8	4.9, 2.0-20.9	0.5557	0.7072
Proportion submicroscopic malaria among NAAT-positives	56	7960	64.1 (57.5-70.5)	97.0	58.9, 0.0-100.0	10	1023	52.9 (40.2-65.5)	92.7	53.4, 26.9-73.7	0.2469	0.2995
**Delivery, maternal blood**												
Submicroscopic malaria	58	20,693	10.2 (7.8-12.7)	97.1	7.9, 0.0-57.1	14	4038	9.9 (5.6-15.3)	96.4	9.6, 0.5-36.8	0.5752	0.8700
Microscopic malaria	58	19,316	3.3 (2.3-4.6)	95.0	2.5, 0.0-42.9	14	4053	7.6 (3.0-13.9)	97.8	6.2, 0.0-39.9	0.0719	0.0459
Proportion submicroscopic malaria among NAAT-positives	58	3631	75.4 (70.1-80.4)	90.3	77.2, 32.6-100	14	797	58.4 (47.7-68.7)	85.9	53.7, 16.7-100	0.0043	0.0173
**Delivery, placental blood**												
Submicroscopic malaria	57	17,164	8.0 (6.1-10.1)	95.6	6.1, 0.0-35.7	13	2801	14.0 (7.7-21.7)	96.7	14.0, 1.5-49.5	0.2009	0.0907
Microscopic malaria	57	16,601	3.4 (1.9-5.2)	96.8	2.4, 0.0-35.2	13	2635	4.0 (0.7-9.5)	97.2	3.3, 0.0-30.1	0.8285	0.9096
Proportion submicroscopic malaria among NAAT-positives	57	3137	70.5 (63.8-76.9)	91.2	69.1, 31.3-100	13	616	81.0 (66.9-92.2)	91.3	84.8, 41.6-100	0.1989	0.0732

*Pooled estimate from Stata procedure metaprop
†Wilcoxon rank-sum test (or Mann-Whitney test): this tests the hypothesis that two independent samples are from populations with the same distribution

Table S23: Sensitivity analysis: Effect of quality assessment factor in multivariable models

	Submicroscopic vs. no malaria		Microscopic vs. no malaria		Submicroscopic vs. microscopic malaria	
	**aOR, 95% CI**	**p-value**	**aOR, 95% CI**	**p-value**	**aOR, 95% CI**	**p-value**
**Africa**			**Pregnancy, maternal blood**			
Quality score (continuous, 2-6)	1.31, 0.82-2.12	0.2606	1.18, 0.75-1.86	0.4780	1.12, 0.74-1.69	0.6069
High quality study (5-6 vs. < 5)	1.52, 0.93-2.51	0.0974	1.24, 0.76-2.04	0.3848	1.13, 0.71-1.82	0.5999
**Americas/Asia**						
Quality score (continuous, 2-6)	0.38, 0.19-0.79	0.0097	0.55, 0.21-1.43	0.2182	0.70, 0.23-2.10	0.5255
High quality study (5-6 vs. < 5)	0.35, 0.13-0.92	0.0337	0.59, 0.18-1.99	0.3965	0.64, 0.17-2.40	0.5099
**Americas***						
Quality score (continuous, 2-6)	0.61, 0.14-2.72	0.5203	1.36, 0.46-3.97	0.5770	0.45, 0.08-2.42	0.3532
High quality study (5-6 vs. < 5)	0.61, 0.14-2.72	0.5203	1.36, 0.46-3.97	0.5770	0.45, 0.08-2.42	0.3532
**Asia***						
Quality score (continuous, 2-6)	0.29, 0.17-0.51	<0.0001	0.33, 0.09-1.21	0.0947	0.90, 0.25-3.20	0.866
High quality study (5-6 vs. < 5)	0.18, 0.07-0.46	0.0004	0.21, 0.03-1.45	0.1129	0.90, 0.15-5.60	0.914
**Africa**			**Delivery, maternal blood**			
Quality score (continuous, 2-6)	0.65, 0.35-1.19	0.1624	0.63, 0.33-1.19	0.1522	1.08, 0.69-1.68	0.7493
High quality study (5-6 vs. < 5)	0.86, 0.35-2.13	0.7476	0.63, 0.25-1.59	0.3253	1.38, 0.73-2.61	0.3280
**Americas/Asia**						
Quality score (continuous, 2-6)	0.38, 0.14-1.05	0.0630	0.70, 0.11-4.40	0.7074	0.55, 0.09-3.36	0.5139
High quality study (5-6 vs. < 5)	0.38, 0.14-1.05	0.0630	0.70, 0.11-4.40	0.7074	0.55, 0.09-3.36	0.5139
**Americas***						
Quality score (continuous, 2-6)	No convergence		No convergence		No convergence	
High quality study (5-6 vs. < 5)	No convergence		No convergence		No convergence	
**Asia***						
Quality score (continuous, 2-6)	0.21, 0.09-0.51	0.0005	0.68, 0.10-4.55	0.6899	0.31, 0.06-1.65	0.1719
High quality study (5-6 vs. < 5)	0.21, 0.09-0.51	0.0005	0.68, 0.10-4.55	0.6899	0.31, 0.06-1.65	0.1719
**Africa**			**Delivery, placental blood**			
Quality score (continuous, 2-6)	0.59, 0.35-1.00	0.0515	0.72, 0.36-1.45	0.3616	0.82, 0.48-1.39	0.4577
High quality study (5-6 vs. < 5)	0.60, 0.29-1.26	0.1779	0.62, 0.24-1.58	0.3145	0.97, 0.47-1.99	0.9394
**Americas/Asia**						
Quality score (continuous, 2-6)	0.27, 0.11-0.70*	0.0071	0.24, 0.03-2.14	0.2022	1.13, 0.12-10.68	0.9171

High quality study (5-6 vs. < 5)	0.27, 0.11-0.70*	0.0071	0.24, 0.03-2.14	0.2022	1.13, 0.12-10.68	0.9171
**America***						
Quality score (continuous, 2-6)	No convergence		No convergence		No convergence	
High quality study (5-6 vs. < 5)	No convergence		No convergence		No convergence	
**Asia***						
Quality score (continuous, 2-6)	0.12, 0.05-0.30	<0.0001	0.30, 0.02-4.03	0.3613	0.40, 0.03-5.00	0.4801
High quality study (5-6 vs. < 5)	0.12, 0.05-0.30	<0.0001	0.30, 0.02-4.03	0.3613	0.40, 0.03-5.00	0.4801

aOR, adjusted Odds ratio. Note: adjusted for baseline model as reported in Table 2, S14, and S15
*Interaction noted between continent and submicroscopic malaria: for this reason, data are additionally presented by continent

Table S24: Sensitivity analysis: Effect of quality of blood smear reading on risk factors for submicroscopic and microscopic malaria, multivariable models in pregnancy, IPD

	**Africa, high transmission areas***							
	**Any malaria infection vs. no malaria**		**Microscopic vs. no malaria**		**Submicroscopic vs. no malaria**		**Risk of submicroscopic infections among NAAT-positive infections**	
	**Base model** N= 4489, 66·1% of available data, 6 sublocations†							
	**aOR, 95% CI**	**p-value**	**aOR, 95% CI**	**p-value**	**aOR, 95% CI**	**p-value**	**aOR, 95% CI**	**p-value**
Age (years)								
<20	**2.43, 1.87-3.17**	<0.0001	**3.16, 2.33-4.29**	<0.0001	**1.62, 1.16-2.26**	0.0050	**0.51, 0.36-0.72**	0.0001
20-29	**1.31, 1.06-1.60**	0.0104	**1.57, 1.22-2.02**	0.0004	1.12, 0.89-1.42	0.34	**0.72, 0.55-0.93**	0.0120
30+ years	Reference		Reference		Reference		Reference	
Gravidity								
G1	**1.36, 1.07-1.73**	0.0108	**2.14, 1.65-2.77**	<0.0001	**0.70, 0.52-0.95**	0.0201	**0.32, 0.25-0.42**	<0.0001
G2	1.02, 0.82-1.26	0.89	**1.36, 1.07-1.72**	0.0133	0.80, 0.62-1.03	0.08	**0.58, 0.46-0.74**	<0.0001
G3+	Reference		Reference		Reference		Reference	
*Pf*PR$_{2\text{-}10}$	**1.04, 1.02-1.06**	0.0008	**1.05, 1.03-1.08**	<0.0001	1.03, 1.00-1.05	0.05	**0.98, 0.96-0.99**	0.0032
Rainy vs. dry season	0.86, 0.73-1.00	0.06	0.93, 0.78-1.10	0.37	**0.70, 0.56-0.87**	0.0012	**0.75, 0.61-0.93**	0.0082
First ANC visit‡	Not included		Not included		Not included		Not included	0·11
Definition of negative slide								
No parasite in 100 high power fields	0.42, 0.11-1.60	0.18	0.40, 0.10-1.58	0.19	0.40, 0.09-1.85	0.24	1.02, 0.67-1.54	0.94
No parasite in 200+ high power fields	Reference		Reference				Reference	
Slide readers								
1 reader + 10% quality control	3.21, 0.84-12.24	0.09	3.21, 0.80-12.85	0.10	3.53, 0.76-16.42	0.11	1.09, 0.69-1.70	0.72
2+ readers	Reference		Reference		Reference		Reference	
	**Africa, moderate-to-low transmission areas***							
	**Any malaria infection vs. no malaria**		**Microscopic vs. no malaria**		**Submicroscopic vs. no malaria**		**Risk of submicroscopic infections among NAAT-positive infections**	
	**Base model** N=5177, 62.3·0% of available data, 17 sublocations†							
	**aOR, 95% CI**	**p-value**	**aOR, 95% CI**	**p-value**	**aOR, 95% CI**	**p-value**	**aOR, 95% CI**	**p-value**
Age (years)								
<20	**2.09, 1.60-2.74**	<0.0001	**2.45, 1.56-3.85**	0.0001	**1.92, 1.41-2.61**	<0.0001	0.78, 0.47-1.29	0.33
20-29	1.16, 0.93-1.43	0.19	1.28, 0.87-1.89	0.22	1.10, 0.87-1.40	0.42	0.86, 0.56-1.32	0.49
30+ years	Reference		Reference		Reference		Reference	
Gravidity								
G1	**1.35, 1.07-1.70**	0.0124	**2.39, 1.69-3.39**	<0.0001	0.97, 0.74-1.28	0.85	**0.41, 0.27-0.60**	<0.0001
G2	1.09, 0.88-1.35	0.44	1.36, 0.97-1.91	0.08	0.98, 0.77-1.25	0.87	0.72, 0.49-1.06	0.09
G3+	Reference		Reference		Reference		Reference	
*Pf*PR$_{2\text{-}10}$	**0.94, 0.92-0.97**	<0.0001	0.96, 0.93-1.00	0.06	**0.94, 0.91-0.97**	0.0001	0.97, 0.94-1.01	0.20
Rainy vs. dry season	0.98, 0.85-1.12	0.76	0.99, 0.81-1.20	0.91	0.97, 0.83-1.14	0.70	0.98, 0.78-1.23	0.86

First ANC visit‡	0.44, 0.20-0.95	0.0378	0.99, 0.48-2.05	0.99	**0.38, 0.16-0.91**	0.0307	**0.38, 0.17-0.85**	0.0189
Definition of negative slide								
No parasite in 100 high power fields	**0.27, 0.13-0.56**	0.0005	**0.31, 0.16-0.60**	0.0006	**0.24, 0.10-0.55**	0.0008	0.77, 0.36-1.66	0.51
No parasite in 200+ high power fields	Reference		Reference		Reference		Reference	
Slide readers								
1 reader + 10% quality control	1.16, 0.44-3.04	0.77	**3.75, 1.60-8.80**	0.0024	0.82, 0.28-2.43	0.72	**0.22, 0.08-0.57**	0.0018
2+ readers	Reference		Reference		Reference		Reference	
**Americas and Asia**								
	**Any malaria infection vs. no malaria**		**Microscopic vs. no malaria**		**Submicroscopic vs. no malaria**		**Risk of submicroscopic infections among NAAT-positive infections**	
	**Model** N=9552, 92·7% of available data, 19 sublocations†							
	**aOR, 95% CI**	**p-value**	**aOR, 95% CI**	**p-value**	**aOR, 95% CI**	**p-value**	**aOR, 95% CI**	**p-value**
Age (years)								
<20	**1.61, 1.23-2.09**	0.0004	**2.40, 1.43-4.06**	0.0010	1.41, 1.05-1.91	0.0245	0.59, 0.33-1.07	0.08
20-29	1.18, 0.97-1.43	0.09	1.52, 0.99-2.35	0.06	1.12, 0.91-1.38	0.29	0.74, 0.46-1.19	0.21
30+ years	Reference		Reference		Reference		Reference	
Gravidity								
G1	0.86, 0.71-1.04	0.12	1.13, 0.78-1.64	0.51	0.78, 0.63-0.97	0.0255	0.69, 0.45-1.05	0.08
G2	0.88, 0.73-1.07	0.20	1.00, 0.68-1.48	0.99	0.86, 0.69-1.06	0.16	0.86, 0.55-1.32	0.48
G3+	Reference		Reference		Reference		Reference	
*Pf*PR$_{2-10}$	**1.04, 1.01-1.06**	0.0015	1.00, 0.96-1.04	0.92	1.06, 1.03-1.09	0.0002	**1.06, 1.01-1.12**	0.0134
Rainy vs· dry season	1.02, 0.87-1.19	0.84	**1.38, 1.01-1.87**	0.0410	0.92, 0.77-1.10	0.38	**0.67, 0.47-0.95**	0.0249
First ANC visit‡	0.67, 0.32-1.40	0.29	3.78, 0.72-19.91	0.12	0.40, 0.14-1.12	0.08	0.14, 0.02-1.17	0.07
Americas vs. Asia	**0.39, 0.21-0.73**	0.0028	0.80, 0.21-3.11	0.75	0.29, 0.13-0.68	0.0046	0.53, 0.09-3.16	0.49
Negative slide definition								
100 high power fields	**0.27, 0.11-0.66**	0.0042	0.25, 0.03-1.91	0.18	0.31, 0.09-1.06	0.06	2.11, 0.14-30.75	0.59
200+ high power fields	Reference		Reference		Reference		Reference	
Slide readers								
1 reader + 10% quality control	**0.54, 0.30-0.98**	0.0427	1.15, 0.32-4.11	0.83	**0.38, 0.17-0.88**	0.0247	0.26, 0.05-1.38	0.11
2+ readers	Reference		Reference		Reference		Reference	

aOR, adjusted Odds ratio. CI, confidence interval. G1, primigravidae. G2, secundigravidae. G3+, multigravidae (excluding secundigravidae). Available data: number of participants with an outcome on submicroscopic, microscopic and no malaria. Factors where the p-value is <0·05 are printed in bold. Note that studies in Asia and the Pacific were included under "Asia"; studies in middle or south America were included under "Americas". * High transmission: *Pf*PR$_{2-10}$ ≥35%, moderate-to-low transmission *Pf*PR$_{2-10}$ <35%. † Available data (data with information on microscopic, submicroscopic and no malaria): Africa, high transmission areas N=6790, 9 sublocations; Africa, moderate-to-low transmission areas N=8306, 24 sublocations; Americas/Asia N=10,305, 23 sublocations. †In Africa: High transmission: *Pf*PR$_{2-10}$ ≥35%, moderate and low transmission combined: *Pf*PR$_{2-10}$ <35%: only 1 study <10% in Africa. In the model for America/Asia, one study in Indonesia, 9% of data, had *Pf*PR$_{2-10}$ of 25%, all other studies *Pf*PR$_{2-10}$ 0-5%, with 81% *Pf*PR$_{2-10}$ of <2%. ‡Comparison group for first ANC visit: not first ANC visit or unknown if first visit or not. First ANC visit was not included in high transmission area because of non-convergence for subgroups (cells with 0 values).

Table S25: Sensitivity analysis: Multivariable analyses of factors associated with submicroscopic and microscopic malaria infections in pregnancy by region, using an alternative measure of transmission level*, IPD

				**Africa, high transmission level***					
		**Any malaria infection vs. no malaria**		**Microscopic vs. no malaria**		**Submicroscopic vs. no malaria**		**Risk of submicroscopic infections among NAAT-positive infections**	
				**Base model** N=9356, 98·5% of available data, 18 sublocations†					
		**aOR, 95% CI**	**p-value**	**aOR, 95% CI**	**p-value**	**aOR, 95% CI**	**p-value**	**aOR, 95% CI**	**p-value**
Age (years)									
<20		**2.43, 2.04-2.91**	<0.0001	**3.42, 2.74-4.28**	<0.0001	**1.72, 1.39-2.14**	<0.0001	**0.50, 0.39-0.65**	<0.0001
20-29		**1.34, 1.17-1.54**	<0.0001	**1.66, 1.39-1.99**	<0.0001	1.12, 0.96-1.31	0.16	**0.67, 0.55-0.82**	<0.0001
30+ years		Reference		Reference		Reference		Reference	
Gravidity									
G1		**1.26, 1.08-1.47**	0.0029	**2.00, 1.67-2.39**	<0.0001	**0.73, 0.60-0.88**	0.0012	**0.36, 0.29-0.45**	<0.0001
G2		0.95, 0.83-1.09	0.49	**1.22, 1.04-1.44**	0.0166	**0.75, 0.64-0.88**	0.0006	**0.61, 0.51-0.74**	<0.0001
G3+		Reference		Reference		Reference		Reference	
Alternative indicator transmission level*		**1.04, 1.03-1.06**	<0.0001	**1.05, 1.01-1.09**	0.0079	**1.04, 1.02-1.06**	<0.0001	0.99, 0.95-1.04	0.68
Rainy vs. dry season		**1.14, 1.04-1.26**	0.0080	**1.23, 1.09-1.38**	0.0006	1.03, 0.91-1.17	0.62	**0.84, 0.73-0.97**	0.0146
First ANC visit‡		**0.66, 0.50-0.87**	0.0034	0.63, 0.28-1.45	0.28	0.74, 0.49-1.09	0.13	1.16, 0.40-3.37	0.79
	Available data (%)			**Africa, high transmission level*** **Base model with additional variables of interest with limited sample size**					
Gestational age (weeks)	98·2	0.99, 0.99-1.00	0.15	1·00, 0·99-1·01	0·62	0·99, 0·98-1·00	0·09	0·99, 0·98-1·01	0·29
HIV-infection	72·2	1.32, 0.95-1.84	0.10	**1·54, 1·02-2·33**	0·0394	1·20, 0·81-1·78	0·36	0·78, 0·49-1·23	0·28
Rural setting	35·1	1.24, 0.91-1.67	0.17	1·33, 0·97-1·81	0·08	1·16, 0·87-1·56	0·31	0·88, 0·65-1·19	0·40
Antimalarial use §	18·2	**0.54, 0.37-0.79**	0.0014	**0·34, 0·22-0·51**	<0·0001	**0·61, 0·41-0·92**	0·0174	**1·83, 1·20-2·80**	0·0054
ITN use §	53·0	0.94, 0.83-1.06	0.32	0·98, 0·85-1·13	0·80	0·92, 0·78-1·09	0·33	0·94 0·78-1·12	0·47
Any net use	87·1	0.92, 0.84-1.02	0.12	0·96, 0·86-1·08	0·54	**0.87, 0·77-0·99**	0·0311	0·90, 0·78-1·04	0·15
IRS	57·2	0.99, 0.93-1.06	0.78	0·97, 0·89-1·05	0·39	1·02, 0·95-1·10	0·61	1·06, 0·97-1·15	0·21
				**Africa, Moderate and low transmission level***					
		**Any malaria infection vs. no malaria**		**Microscopic vs. no malaria**		**Submicroscopic vs. no malaria**		**Risk of submicroscopic infections among NAAT-positive infections**	
				**Base model** N=5194, 99·5% of available data, 13 sublocations†					
		**aOR, 95% CI**	**p-value**	**aOR, 95% CI**	**p-value**	**aOR, 95% CI**	**p-value**	**aOR, 95% CI**	**p-value**
Age (years)									
<20		**2.12, 1.63-2.76**	<0.0001	**2.76, 1.80-4.25**	<0.0001	**1.90, 1.40-2.59**	<0.0001	0.69, 0.42-1.12	0.14
20-29		1.18, 0.98-1.42	0.08	**1.44, 1.02-2.03**	0.0367	1.11, 0.90-1.36	0.32	0.77, 0.53-1.12	0.17
30+ years		Reference		Reference		Reference		Reference	
Gravidity									
G1		**1.76, 1.40-2.20**	<0.0001	**2.21, 1.56-3.11**	<0.0001	**1.54, 1.18-2.01**	0.0014	0.70, 0.47-1.04	0.08

G2		**1.41, 1.15-1.73**	0.0008	**1.51, 1.09-2.09**	0.0127	**1.39, 1.10-1.75**	0.0054	0.92, 0.64-1.33	0.66
G3+		Reference		Reference		Reference		Reference	
Alternative indicator transmission level*		**1.09, 1.02-1.17**	0.0072	1.04, 0.98-1.10	0.19	**1.12, 1.04-1.21**	0.0024	**1.08, 1.03-1.14**	0.0027
Rainy vs. dry season		0.89, 0.77-1.02	0.09	0.90, 0.73-1.11	0.33	0.88, 0.75-1.04	0.13	0.98, 0.77-1.25	0.86
First ANC visit‡		1.37, 0.69-2.73	0.37	0.89, 0.49-1.64	0.72	1.66, 0.76-3.63	0.21	**1.85, 1.09-3.14**	0.0224
	Available data (%)	**Africa, Moderate and low transmission level* Base model with additional variables of interest with limited sample size**							
Gestational age (weeks)	84·5	**0.97, 0.96-0.99**	0.0022	**0·95, 0·93-0.98**	0·0002	0·99, 0·97-1·01	0·28	**1·01, 1·01-1·07**	0·0145
HIV-infection	89·7	1.28, 0.90-1.80	0.17	1·48, 0·81-2·72	0·21	1·08, 0·70-1·67	0·72	0·73, 0·35-1·51	0·40
Rural setting §	26·9	1.03, 9.81-1.31	0.80	0·52, 0·10-2·67	0·43	0·86, 0·26-2·79	0·80	1·64, 0·27-9·90	0·59
Antimalarial use §	20·9	**0.58, 0.37-0.88**	0.0116	0·89, 0·52-1·54	0·68	**0·38, 0·21-0·68**	0·0012	**0·44, 0·20-0·95**	0·0377
ITN use	16·7	0.95, 0.62-1.43	0.79	Non-convergence		Non-convergence		Non-convergence	
Any net use	58·9	0.83, 0.68-1.01	0.06	0·75, 0·56-1·00	0·05	0.86, 0·68-1·99	0·22	1·15, 0·81-1·63	0·43
IRS §	36·2	1.39, 0.81-2.37	0.23	1·57, 0·77-3·20	0·22	1·24, 0·62-2·50	0·55	0·79, 0·32-1·99	0·62
		**Americas and Asia**							
		**Any malaria infection vs. no malaria**		**Microscopic vs. no malaria**		**Submicroscopic vs. no malaria**		**Risk of submicroscopic infections among NAAT-positive infections**	
		**Base model** N=10,068, 98·1% of available data, 22 sublocations†							
		**aOR, 95% CI**	**p-value**	**aOR, 95% CI**	**p-value**	**aOR, 95% CI**	**p-value**	**aOR, 95% CI**	**p-value**
Age (years)									
<20		**1.57, 1.22-2.01**	0.0004	**2.75, 1.73-4.38**	<0.0001	1.31, 0.98-1.73	0.07	**0.48, 0.29-0.80**	0.0052
20-29		1.17, 0.98-1.40	0.09	**1.66, 1.13-2.43**	0.0097	1.09, 0.89-1.32	0.42	**0.65, 0.43-0.99**	0.0428
30+ years		Reference		Reference		Reference		Reference	
Gravidity									
G1		0.96, 0.80-1.15	0.68	1.20, 0.87-1.65	0.26	0.88, 0.72-1.08	0.21	0.72, 0.51-1.03	0.07
G2		1.00, 0.84-1.20	0.99	1.03, 0.73-1.46	0.85	0.99, 0.82-1.21	0.96	0.96, 0.66-1.40	0.83
G3+		Reference		Reference		Reference		Reference	
Alternative indicator transmission level*		**1.05, 1.03-1.07**	<0.0001	**1.04, 1.01-1.07**	0.0124	**1.05, 1.03-1.07**	<0.0001	1.01, 0.98-1.04	0.60
Rainy vs. dry season		1.04, 0.89-1.20	0.65	1.23, 0.95-1.60	0.11	0.97, 0.82-1.15	0.75	0.79, 0.60-1.05	0.11
First ANC visit‡		1.48, 0.73-3.00	0.28	**7.13, 1.80-28.24**	0.0051	0.96, 0.39-2.35	0.93	**0.15, 0.04-0.55**	0.0044
Americas vs. Asia		1.28, 0.69-2.37	0.43	2.31, 0.67-7.93	0.18	1.06, 0.48-2.31	0.89	0.45, 0.13-1.52	0.20
	Available data (%)	**Americas and Asia Base model with additional variables of interest with limited sample size ¶**							
Gestational age (weeks)	96·5	1.00, 0.99-1.01	0.48	0·99, 0·97-1·01	0·16	1·00, 0·99-1·01	0·95	1·01, 0·99-1·04	0·19
Rural setting	81·0	**1.47, 1.22-1.78**	**<0.0001**	**1·73, 1·24-2·42**	0·0014	**1·36, 1·09-1·71**	0·0069	0·80, 0·54-1·18	0·26
Antimalarial use	52·7	1.27, 0.93-1.73	0.14	1·38, 0·90-2·13	0·14	1·20, 0·83-1·76	0·34	0·87, 0·52-1·46	0·60
ITN use	53·8	1.04, 0.82-1.31	0.75	1·05, 0·74-1·49	0·77	1·07, 0·80-1·42	0·64	1·02, 0·67-1·55	0·94
Any net use	88·5	1.13, 0.97-1.32	0.12	1·17, 0·88-1·54	0·28	1·11, 0·93-1·32	0·24	0·95, 0·69-1·29	0·74

IRS	36·8	1.03, 0.76-1.39	0.87	0·85, 0·38-1·89	0·69	1·04, 0·75-1·44	0·80	1·23, 0·52-2·88	0·63

aOR, adjusted Odds ratio. CI, confidence interval. G1, primigravidae. G2, secundigravidae. G3+, multigravidae (excluding secundigravidae). ITN, insecticide treated net. IRS, indoor residual spraying. Available data: number of participants with an outcome on submicroscopic, microscopic and no malaria. Factors where the p-value is <0·05 are printed in bold. Note that studies in Asia and the Pacific were included under "Asia"; studies in central or south America were included under "Americas".

*Alternative measure of transmission level: Parasite prevalence among all gravidae, measured in the first trimester by NAAT (PCR or LAMP). Africa, high transmission: alternative measure ≥35%; Africa, moderate-to-low transmission: alternative measure <35%: only 1 study (two sublocations). †Available data (data with information on microscopic, submicroscopic or no malaria): Africa, high transmission areas: N=9479, 19 sublocations; Africa, moderate-to-low transmission areas in Africa; N=5222, 13 sublocations; Americas/Asia: N=10,261, 22 sublocations. ‡Comparison group for first ANC visit: Not first ANC visit or unknown if first visit or not. § Africa, high transmission: The alternative indicator was not included in the model of antimalarial use because of non-convergence. Africa, moderate-to-low transmission: In model for rural setting, first ANC visit not included because of non-convergence. In model for antimalarial use only age and rainy season included in models with no malaria as reference, and only rainy season included in submicroscopic vs. microscopic model because of non-convergence. Model for IRS conducted without indicator of malaria transmission and gravidity collapsed into primigravidae versus multigravidae because of non-convergence. ¶ There was insufficient information on HIV infection in studies in the Americas and Asia.

Supplemental Figures

Figure S1: Map of included studies

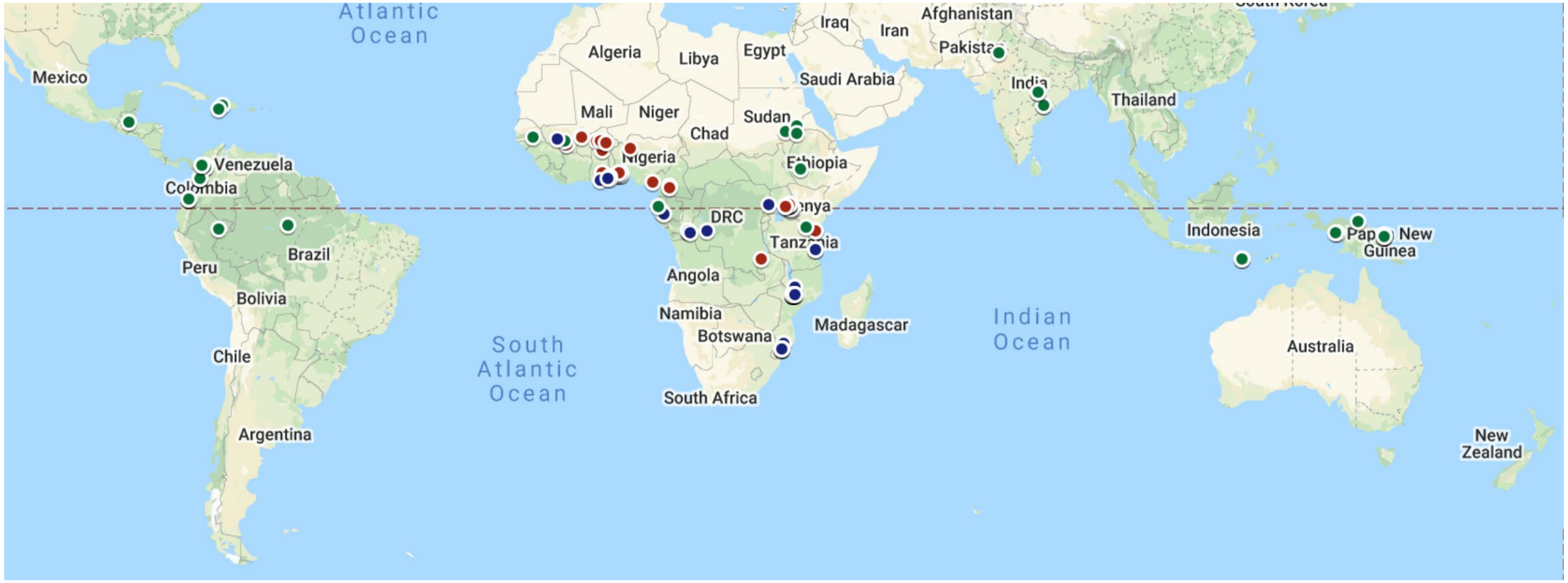

Legend: Green: *Pf*PR$_{2\text{-}10}$ < 10%. Blue: *Pf*PR$_{2\text{-}10}$ 10-34%. Red: *Pf*PR$_{2\text{-}10}$ ≥ 35%.

Figure S2A: Proportion of submicroscopic malaria among NAAT-positive test results by malaria transmission level among first ANC attendees

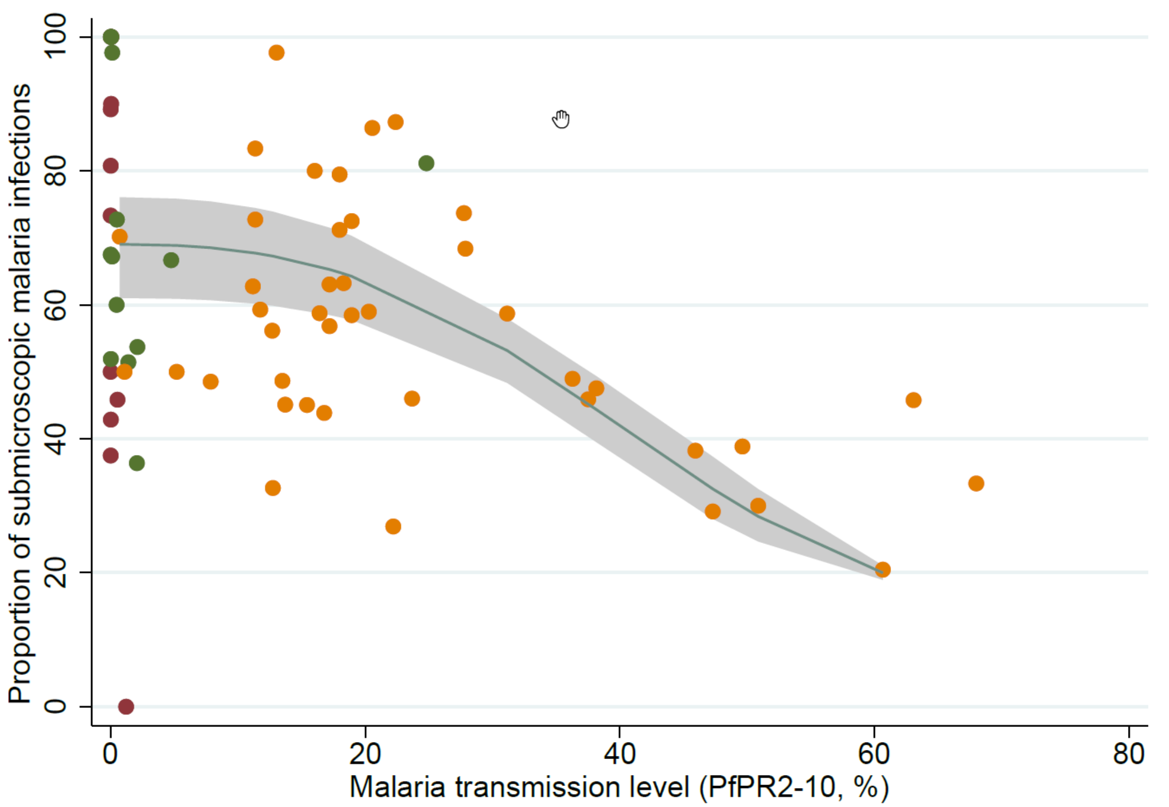

NAAT, Nucleic acid amplification test (PCR or LAMP). *Pf*$PR_{2\text{-}10}$, *Plasmodium falciparum* prevalence among children aged 2-10 years at the year of study visit, as estimated by the Malaria Atlas Project. Note that studies in Asia and the Pacific were combined under "Asia"; studies in middle or south America were combined under "Americas". Markers: Orange: studies in Africa; Green: studies in Asia; Red: studies in the Americas. The relationship and 95% CI were estimated for Africa only using fractional polynomials logistic regression with robust variance (p<0.0001 for comparison with linear and non-covariate model).

Figure S2B: Proportion of submicroscopic malaria among NAAT-positive test results in pregnancy by malaria transmission level by gravidity

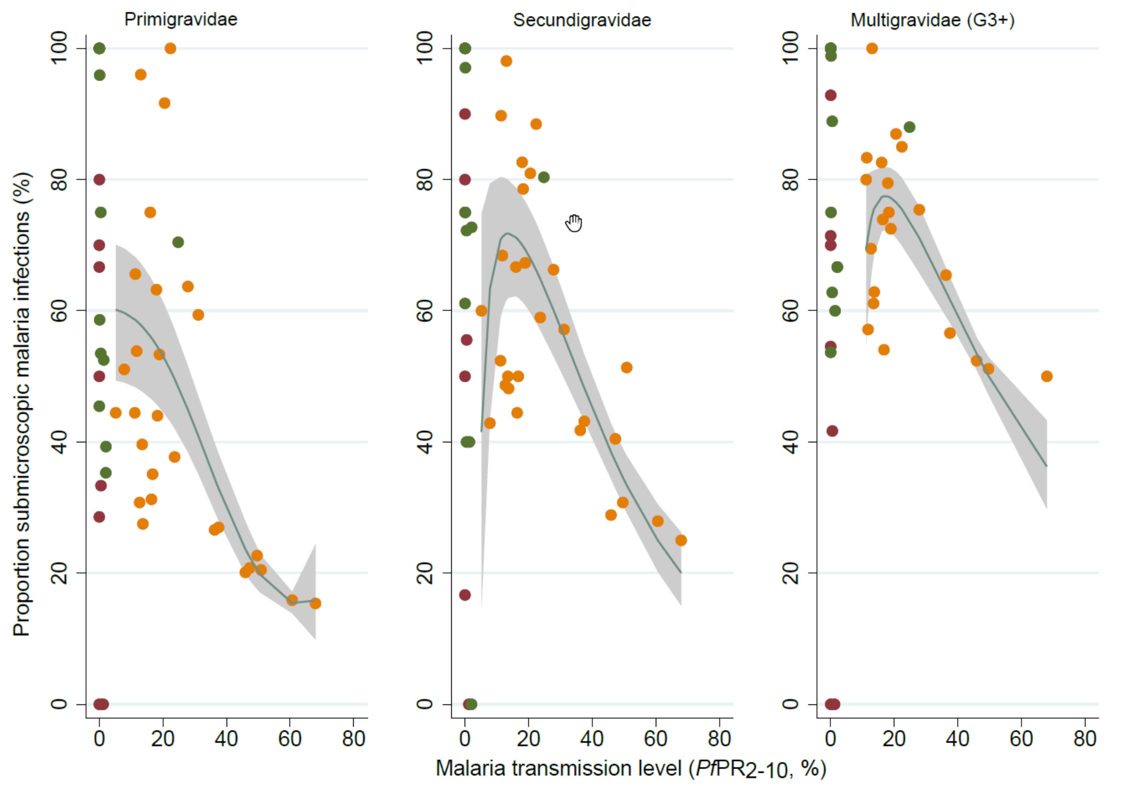

NAAT, Nucleic acid amplification test (PCR or LAMP). *Pf*$PR_{2\text{-}10}$, *Plasmodium falciparum* prevalence among children aged 2-10 years at the year of study visit, as estimated by the Malaria Atlas Project. Note that studies in Asia and the Pacific were combined under "Asia"; studies in middle or south America were combined under "Americas".

Markers: Orange: studies in Africa; Green: studies in Asia; Red: studies in the Americas. The relationship and 95% CI were estimated for Africa only, using fractional polynomials logistic regression with robust variance (primigravidae p=0.004 and p<0.0001 for comparison with linear and non-covariate model, respectively; secundigravidae p=0.008 and p<0.0001, respectively; multigravidae p=0.011 and p<0.0001, respectively)

Figure S1: Probability of fever by malaria status, gestational age and region

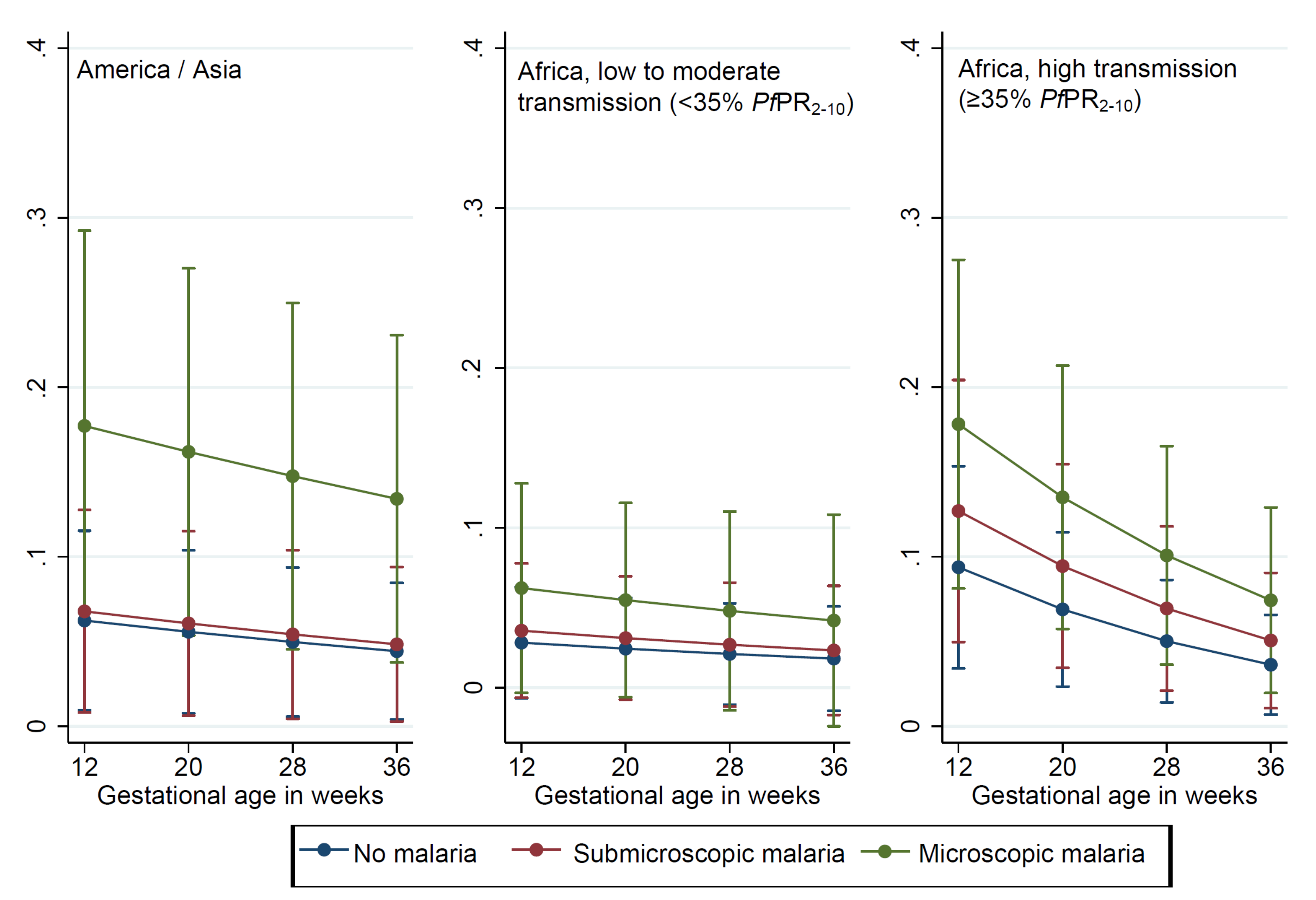

Notes: Individual participant data, using one-stage model for fever during pregnancy. The adjusted odds ratios (aOR) and 95% confidence intervals for gestational age were overall 0.96, 0.94-0.98 (p=0.0007), America/Asia aOR=0.98, 0.97- 0.99s16 (p=0.4816), Africa, moderate-low transmission, aOR=0.98, 0.92-1.04 (p=0.1400), and Africa high transmission, aOR=0.95, 0.93-0.97 (p=0.0007). For additional information, see Table S10.

Figure S2: Prevalence of submicroscopic and microscopic malaria in studies with regular follow up visit in pregnancy by gestational age

<table>
<tr><td colspan="2">Prevalence of microscopic malaria in orange, of submicroscopic malaria in blue, and proportion of submicroscopic among NAAT+ infections in grey</td></tr>
<tr><td>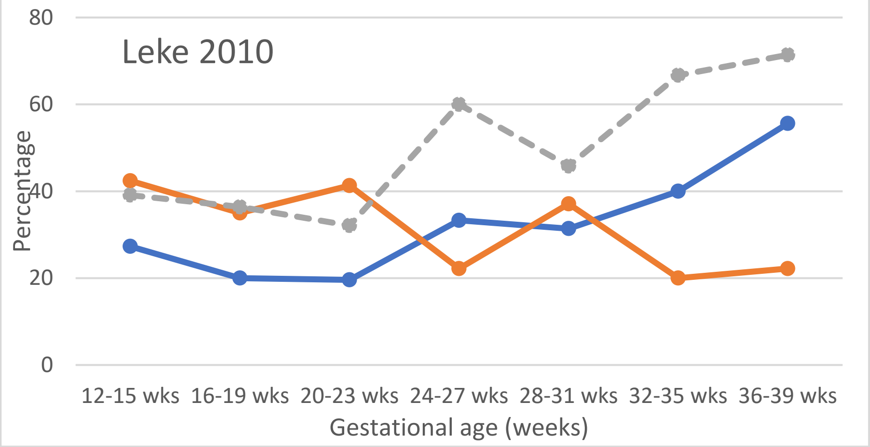
</td><td></td></tr>
<tr><td>Location: Cameroon, 2001-2004, N=91 at enrolment, PfPR$_{2-10}$ 68%, IPTp 0%, ITN or net 0%, 53 (58%) reported antimalarial use in pregnancy.</td><td></td></tr>
<tr><td>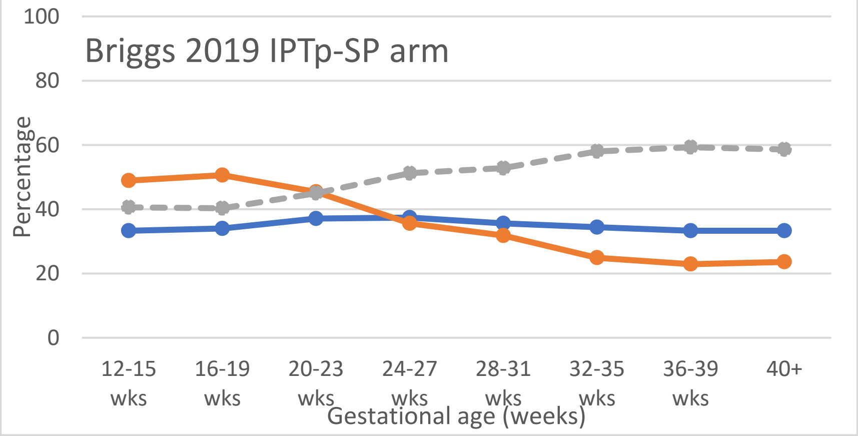
</td><td>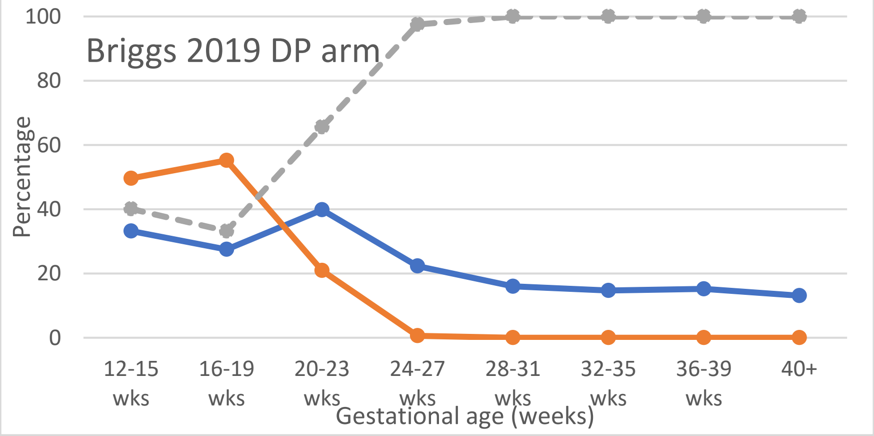
</td></tr>
<tr><td>Location: Uganda, 2016-2017, N=391 for SP arm, PfPR$_{2-10}$ 45.9%, IPTp (2+ doses) 93.1%, ITN received at enrolment</td><td>Location: Uganda, 2016-2017, N=391 for DP arm, PfPR$_{2-10}$ 45.9%, IPTp (2+ doses) 93.9%, ITN received at enrolment</td></tr>
</table>

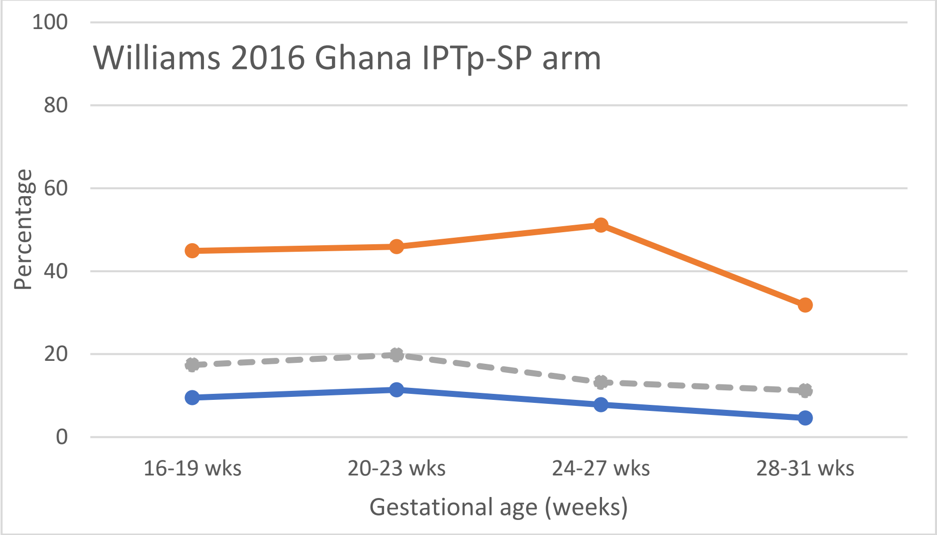

Location: Ghana, 2010-2012, N=653 for SP arm, *Pf*$PR_{2\text{-}10}$ 60.7%, IPTp (2+ doses) 88.7%, ITN received at enrolment

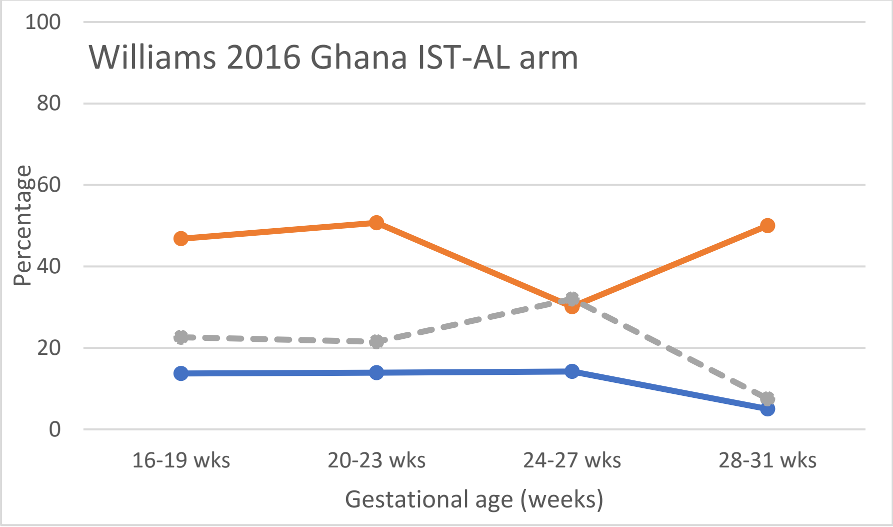

Location: Ghana, 2010-2012, N=653 for IST arm, *Pf*$PR_{2\text{-}10}$ 60.7%, ≥3 cRDTs received: 61.4%, ITN received at enrolment

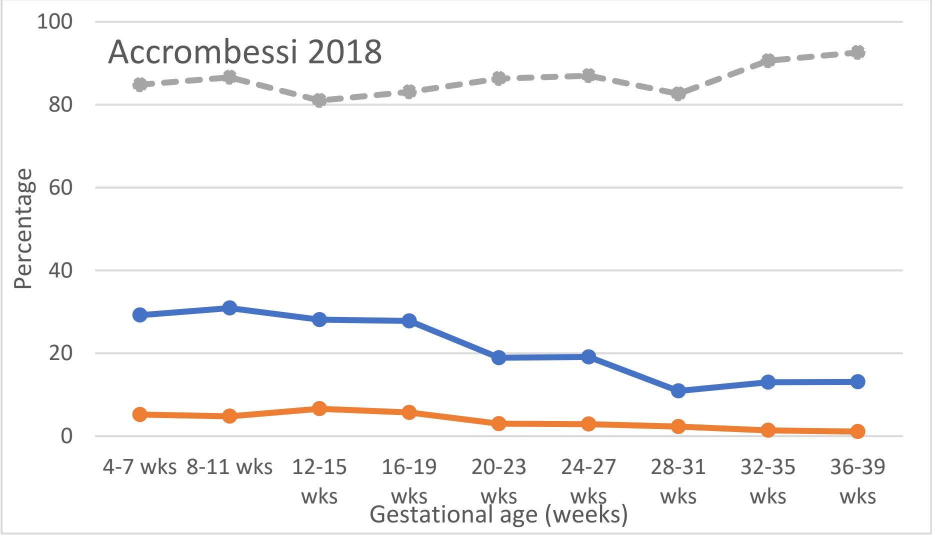

Location: Benin, 2014-2017, N=393, *Pf*$PR_{2\text{-}10}$ 20.6%, IPTp (2+ doses) 76.5%, ITN received at enrolment

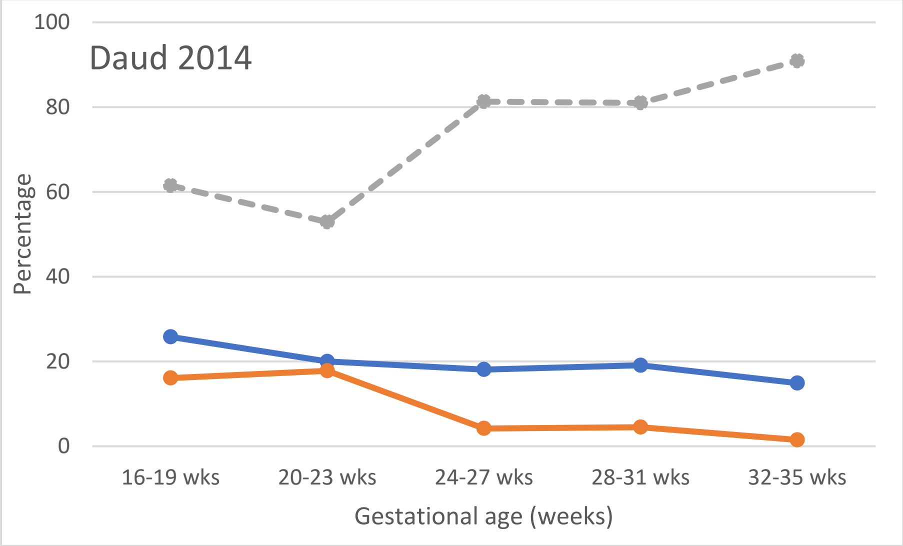

Location: Kenya, 2011-2012, N=111, *Pf*$PR_{2\text{-}10}$ 20.3%, IPTp (2+ doses) provided but no information, no information on ITN use

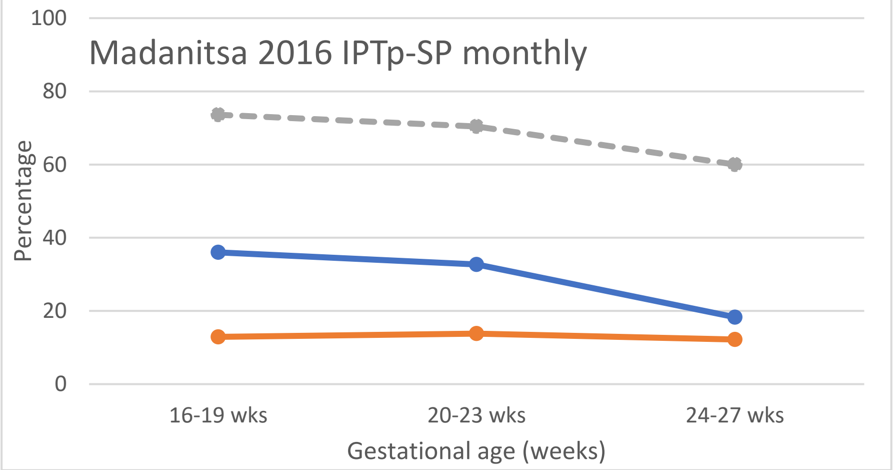

Location: Malawi, 2011-2013, N=921 for SP arm, *Pf*PR$_{2-10}$ 16.1%, IPTp (2+ doses) 96.2%, ITN received at enrolment

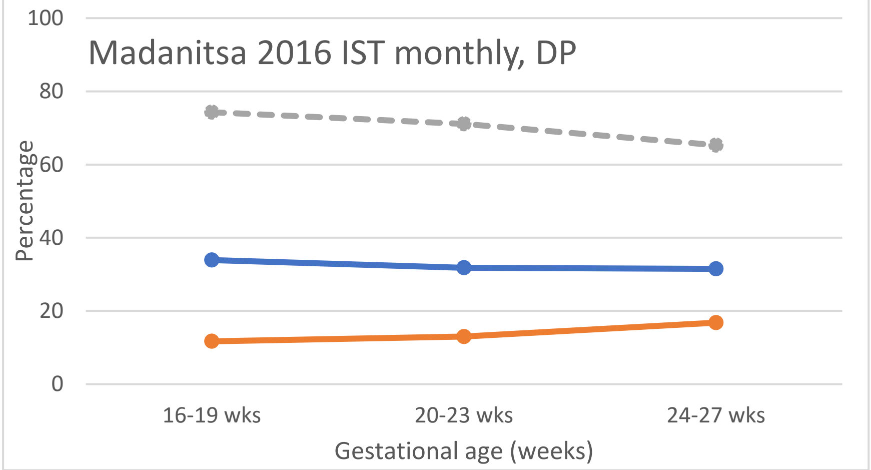

Location: Malawi, 2011-2013, N=923 for IST arm, *Pf*PR$_{2-10}$ 16.1%, ≥3 cRDTs received: 89.9%, ITN received at enrolment

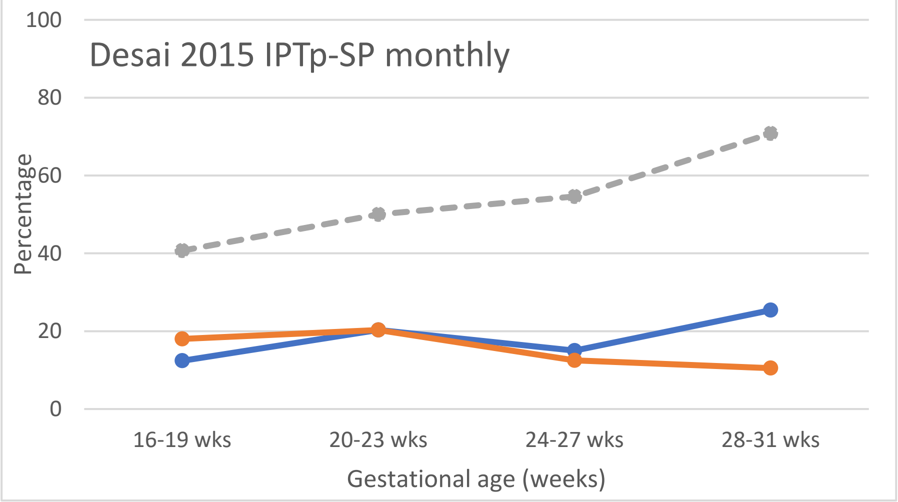

Location: Kenya, 2012-2014, N=514 for SP arm, *Pf*PR$_{2-10}$ 13.8%, IPTp (2+ doses) 90.7%, ITN received at enrolment

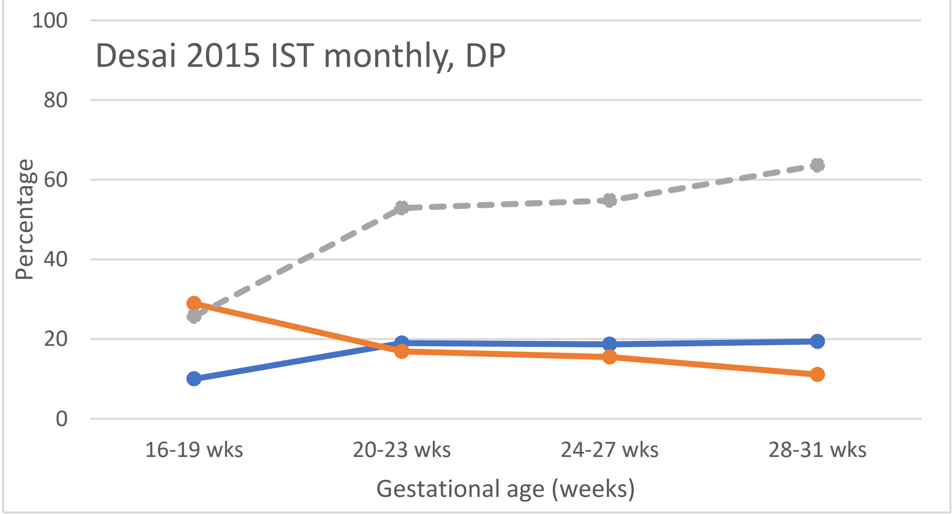

Location: Kenya, 2012-2014, N=514 for IST arm, *Pf*PR$_{2-10}$ 13.8%, ≥3 cRDTs received: 80.7%, ITN received at enrolment

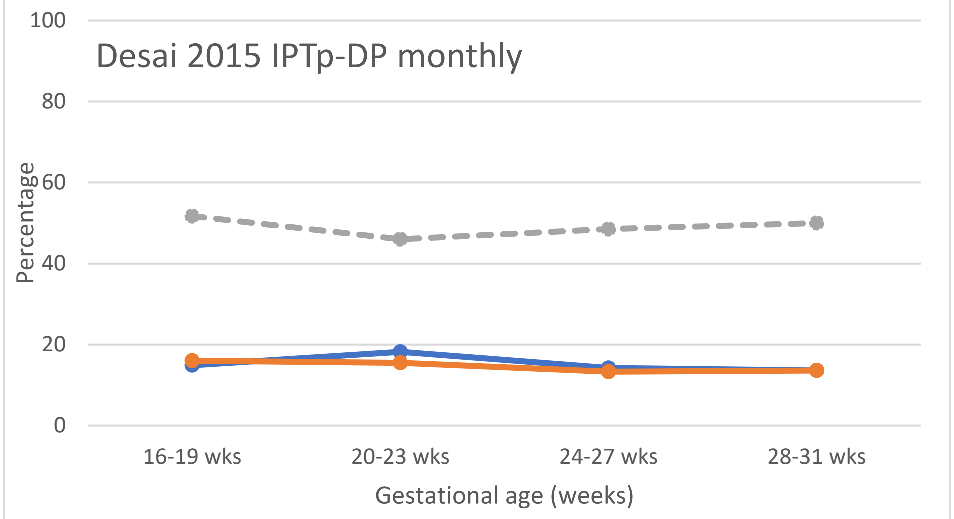

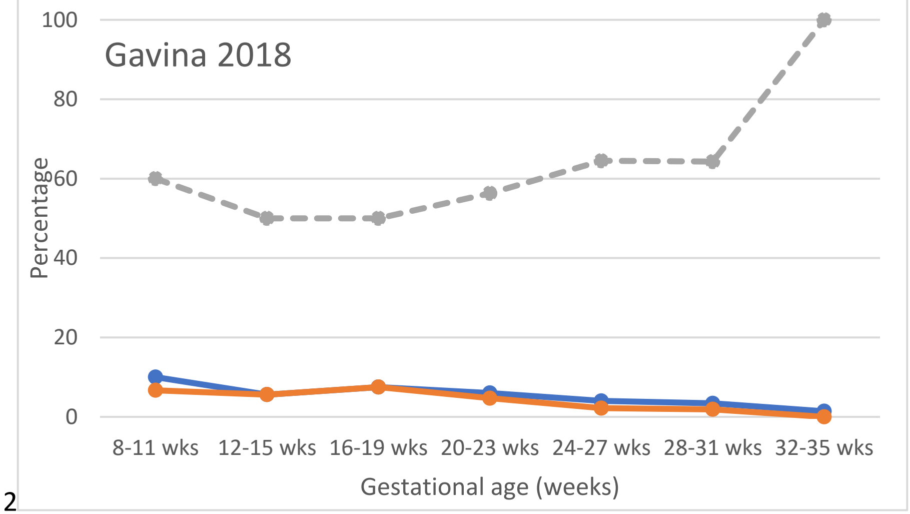

Ble 2

Location: Kenya, 2012-2014, N=515 for DP arm, *Pf*PR_{2-10} 13.8%, IPTp (2+ doses) 92.4%, ITN received at enrolment

Location: Columbia, 2014-2017, N=186, *Pf*PR_{2-10} 0.0%, no IPTp policy, ITN 52.9%, antimalarial use 94.9%

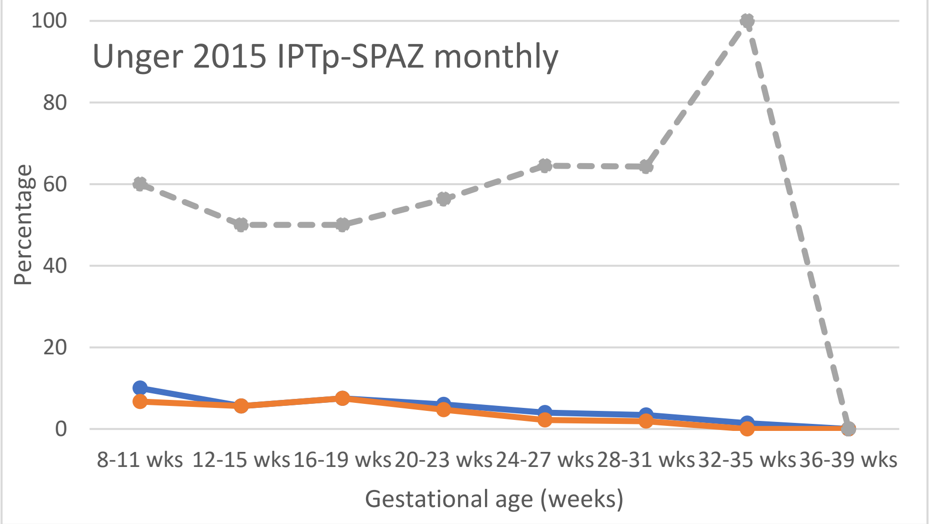

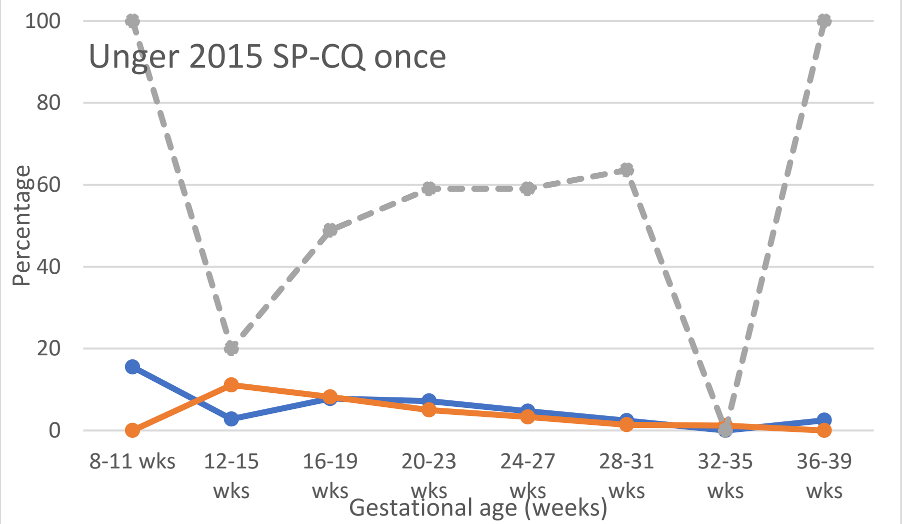

Location: Papua New Guinea, 2014-2017, N=1125 for SPAZ arm, *Pf*PR_{2-10} 1.1%, IPTp (2+ doses) 95.2%, ITN 92.0%

Location: Papua New Guinea, 2014-2017, N=1117 for SP-CQ arm, *Pf*PR_{2-10} 1.1%, ITN 92.0%

Abbreviations: AL, artemether-lumefantrine. AZ, azithromycin. CQ, chloroquine. DP, dihydroartemisinin–piperaquine**.** IPTp, intermittent preventive treatment in pregnancy. IST, intermittent screening and treatment. ITN, insecticide treated nets. *Pf*PR_{2-10}, *Plasmodium falciparum* prevalence in children 2-10 years of age as an indicator of level of malaria transmission at the midyear of study, Malaria Atlas Project. SP: sulfadoxine-pyrimethamine

Figure S3: Proportion of submicroscopic malaria (forest plot), microscopic malaria (column) and no malaria (column) at a subsequent scheduled follow up visit in pregnancy among women with submicroscopic malaria at enrolment, by region

Study	Location	Country	*Pf*PR2-10	No malaria visit 2, submicroscopic visit 1	Microscopic visit 2, submicroscopic visit 1	Submicroscopic visit 2 and visit 1	Fraction (95% CI) Submicroscopic at visit 2	% Weight
**Asia/Americas**								
Gavina 2018	Puerto Libertador	Colombia	0.00	100.0 (10/10)	0.0 (0/10)	0.0 (0/10)	0.00 (0.00, 0.31)	3.90
Unger 2015	Madang	PNG	0.47	100.0 (41/41)	0.0 (0/41)	0.0 (0/41)	0.00 (0.00, 0.09)	5.47
Unger 2015	Mugil	PNG	0.49	100.0 (6/6)	0.0 (0/6)	0.0 (0/6)	0.00 (0.00, 0.46)	3.16
Unger 2015	Alexishafen-U	PNG	1.39	88.9 (8/9)	0.0 (0/9)	11.1 (1/9)	0.11 (0.00, 0.48)	3.75
Unger 2015	Yagaum	PNG	2.06	100.0 (4/4)	0.0 (0/4)	0.0 (0/4)	0.00 (0.00, 0.60)	2.59
Subtotal (I^2 = 0.0%, p = 0.51)							0.00 (0.00, 0.03)	18.87
**Africa**								
Desai 2015	Lwak	Kenya	11.17	73.5 (50/68)	8.8 (6/68)	17.6 (12/68)	0.18 (0.09, 0.29)	5.78
Madanitsa 2016	Chikwawa, G1-2	Malawi	11.35	77.5 (62/80)	6.3 (5/80)	16.3 (13/80)	0.16 (0.09, 0.26)	5.85
Madanitsa 2016	Chikwawa, G3-5	Malawi	11.35	92.3 (12/13)	0.0 (0/13)	7.7 (1/13)	0.08 (0.00, 0.36)	4.27
Desai 2015	Madiany	Kenya	13.49	78.9 (30/38)	7.9 (3/38)	13.2 (5/38)	0.13 (0.04, 0.28)	5.41
Desai 2015	Siaya	Kenya	13.71	74.1 (20/27)	7.4 (2/27)	18.5 (5/27)	0.19 (0.06, 0.38)	5.11
Desai 2015	Bondo	Kenya	16.77	78.7 (37/47)	8.5 (4/47)	12.8 (6/47)	0.13 (0.05, 0.26)	5.56
Madanitsa 2016	Mpemba, G3-5	Malawi	17.98	83.3 (55/66)	1.5 (1/66)	15.2 (10/66)	0.15 (0.08, 0.26)	5.76
Madanitsa 2016	Mpemba, G1-2	Malawi	17.98	73.3 (129/176)	5.7 (10/176)	21.0 (37/176)	0.21 (0.15, 0.28)	6.10
Madanitsa 2016	Madziabango, G1-2	Malawi	18.93	50.9 (29/57)	8.8 (5/57)	40.4 (23/57)	0.40 (0.28, 0.54)	5.68
Madanitsa 2016	Madziabango, G3-5	Malawi	18.93	62.5 (30/48)	6.3 (3/48)	31.3 (15/48)	0.31 (0.19, 0.46)	5.58
Daud 2014	Chulaimbo	Kenya	20.28	54.5 (12/22)	9.1 (2/22)	36.4 (8/22)	0.36 (0.17, 0.59)	4.91
Accrombessi 2018	Soava and Akassato	Benin	20.55	35.2 (32/91)	8.8 (8/91)	56.0 (51/91)	0.56 (0.45, 0.66)	5.91
Briggs 2019	Busia district	Uganda	45.93	13.2 (21/159)	37.7 (60/159)	49.1 (78/159)	0.49 (0.41, 0.57)	6.08
Williams 2016	Navrongo	Ghana	60.65	77.5 (86/111)	13.5 (15/111)	9.0 (10/111)	0.09 (0.04, 0.16)	5.98
Leke 2010	Ngali II	Cameroon	67.99	33.3 (2/6)	33.3 (2/6)	33.3 (2/6)	0.33 (0.04, 0.78)	3.16
Subtotal (I^2 = 89.0%, p < 0.0001)							0.24 (0.16, 0.33)	81.13
Heterogeneity between groups: p < 0.0001								
Overall (I^2 = 88.91%, p < 0.0001)							0.18 (0.11, 0.27)	100.00

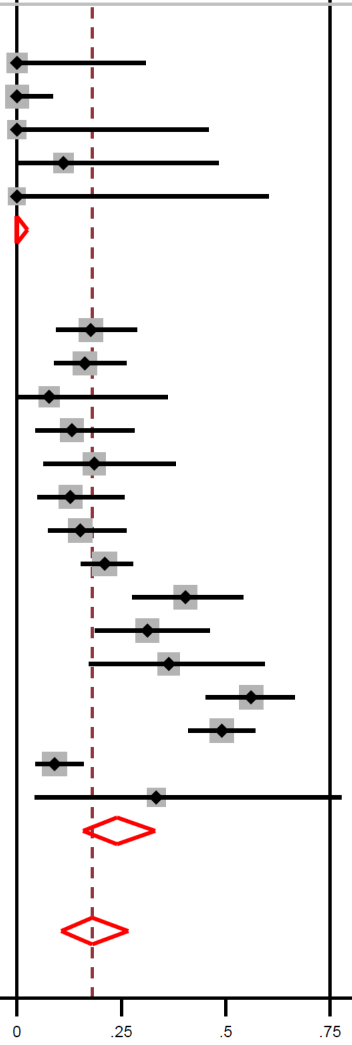

CI, confidence interval. PNG, Papua New Guinea. *Pf*PR$_{2\text{-}10}$, *Plasmodium falciparum* prevalence in children 2-10 years of age as an indicator of level of malaria transmission at the midyear of study, Malaria Atlas Project.

Figure S4: Probability and 95% confidence interval of microscopic and submicroscopic malaria by gestational age

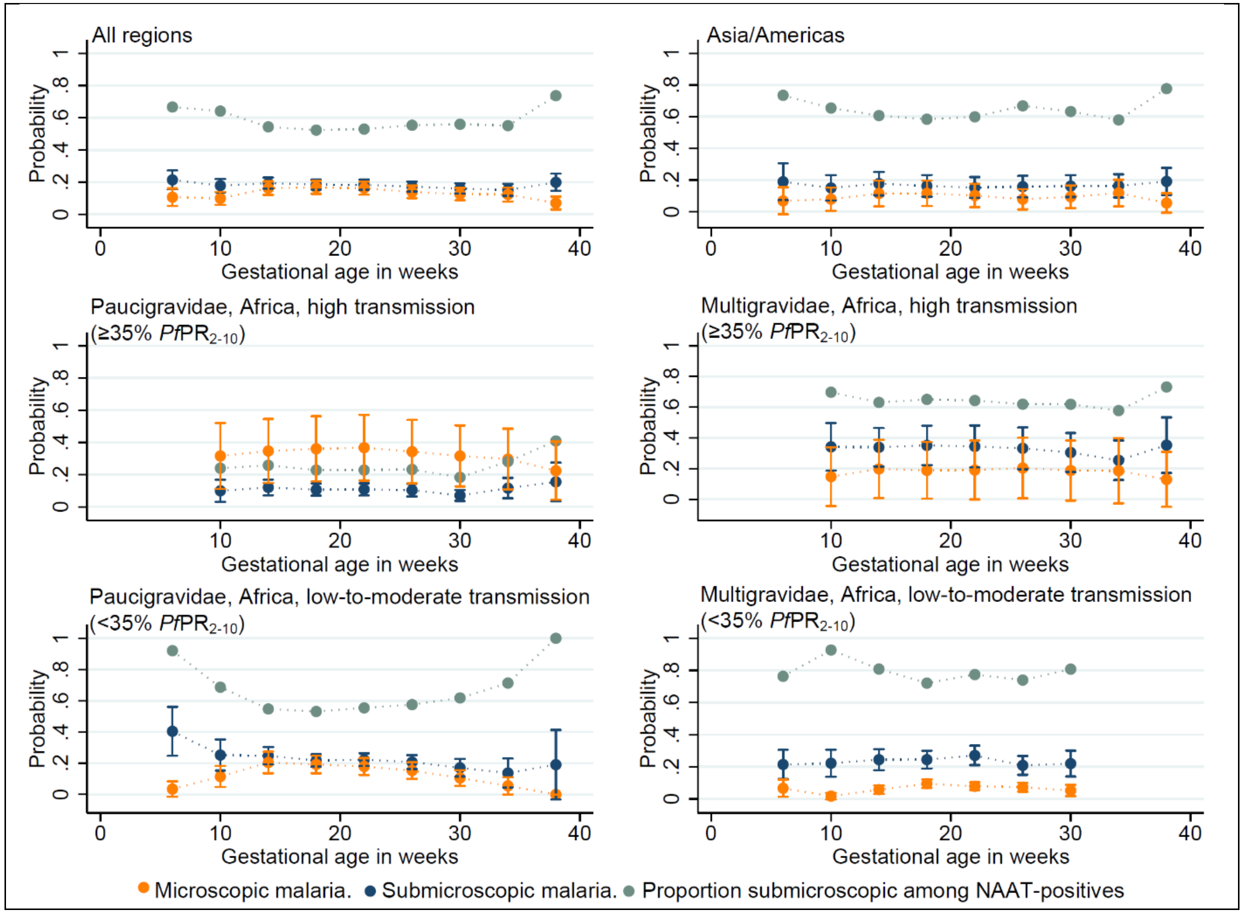

*Pf*PR$_{2\text{-}10}$, *Plasmodium falciparum* prevalence in children 2-10 years of age as an indicator of level of malaria transmission at the midyear of study, Malaria Atlas Project. Estimates were from models adjusted for age, type of gestational age assessment, and year of study.

Notes: In high malaria transmission areas there was not sufficient information before 10 weeks of gestational age. For G3+ in moderate-to-low transmission areas, there was insufficient information after 30 weeks of gestational age.

Figure S7: Funnel plot for studies with information on submicroscopic malaria infection during pregnancy

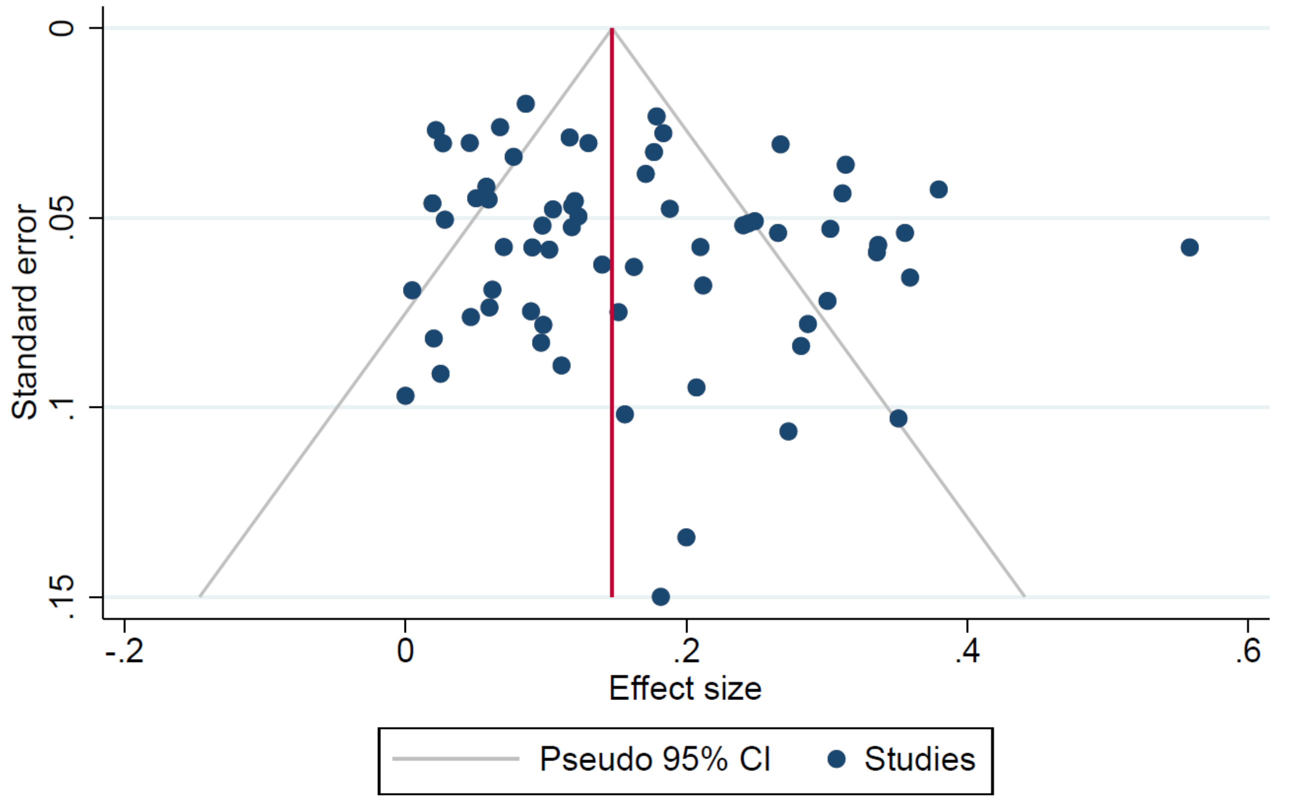

Egger test (a test for asymmetry of the funnel plot): p=0.43.

References

1. Bardaji A, Martinez-Espinosa FE, Arevalo-Herrera M, et al. Burden and impact of *Plasmodium vivax* in pregnancy: A multi-centre prospective observational study. *PLoS Negl Trop Dis* 2017; **11**(6): e0005606.
2. Elbadry MA, Tagliamonte MS, Raccurt CP, et al. Submicroscopic malaria infections in pregnant women from six departments in Haiti. *Trop Med Int Health* 2017; **22**(8): 1030-6.
3. Gavina K, Gnidehou S, Arango E, et al. Clinical outcomes of submicroscopic infections and correlates of protection of VAR2CSA antibodies in a longitudinal study of pregnant women in Colombia. *Infect Immun* 2018; **86**(4).
4. Vasquez AM, Medina AC, Tobon-Castano A, et al. Performance of a highly sensitive rapid diagnostic test (HS-RDT) for detecting malaria in peripheral and placental blood samples from pregnant women in Colombia. *PLoS One* 2018; **13**(8): e0201769.
5. Vásquez AM, Vélez G, Medina A, et al. Evaluation of highly sensitive diagnostic tools for the detection of *P. falciparum* in pregnant women attending antenatal care visits in Colombia. *BMC Pregnancy Childbirth* 2020; **20**(1): 440.
6. Parekh FK, Davison BB, Gamboa D, Hernandez J, Branch OH. Placental histopathologic changes associated with subclinical malaria infection and its impact on the fetal environment. *Am J Trop Med Hyg* 2010; **83**(5): 973-80.
7. Arango EM, Samuel R, Agudelo OM, Carmona-Fonseca J, Maestre A, Yanow SK. Molecular detection of malaria at delivery reveals a high frequency of submicroscopic infections and associated placental damage in pregnant women from northwest Colombia. *Am J Trop Med Hyg* 2013; **89**(1): 178-83.
8. Agudelo O, Arango E, Maestre A, Carmona-Fonseca J. Prevalence of gestational, placental and congenital malaria in north-west Colombia. *Malar J* 2013; **12**(1): 341.
9. Stanisic DI, Moore KA, Baiwog F, et al. Risk factors for malaria and adverse birth outcomes in a prospective cohort of pregnant women resident in a high malaria transmission area of Papua New Guinea. *Trans R Soc Trop Med Hyg* 2015; **109**(5): 313-24.
10. Singh N, Bharti PK, Singh MP, et al. What is the burden of submicroscopic malaria in pregnancy in central India? *Pathog Glob Health* 2015; **109**(1): 30-8.
11. Unger HW, Ome-Kaius M, Wangnapi RA, et al. Sulphadoxine-pyrimethamine plus azithromycin for the prevention of low birthweight in Papua New Guinea: a randomised controlled trial. *BMC Med* 2015; **13**(1): 9.
12. Pava Z, Burdam FH, Handayuni I, et al. Submicroscopic and asymptomatic *Plasmodium* parasitaemia associated with significant risk of anaemia in Papua, Indonesia. *PLoS One* 2016; **11**(10): e0165340.
13. Ahmed R, Poespoprodjo JR, Syafruddin D, et al. Efficacy and safety of intermittent preventive treatment and intermittent screening and treatment versus single screening and treatment with dihydroartemisinin-piperaquine for the control of malaria in pregnancy in Indonesia: a cluster-randomised, open-label, superiority trial. *Lancet Infect Dis* 2019; **19**(9): 973-87.
14. Ahmed R, Asih PPB, Noviyanti R, et al. The clinical burden of microscopically patent and sub-microscopic *P. falciparum* and *P. vivax* malaria in pregnancy in Indonesia. Challenges in malaria research: Core science and innovation, Oxford, UK 22-24 September 2014. p. P2.
15. Ahmed R, Levy EI, Maratina SS, et al. Performance of four HRP-2/pLDH combination rapid diagnostic tests and field microscopy as screening tests for malaria in pregnancy in Indonesia: a cross-sectional study. *Malar J* 2015; **14**(1): 420.
16. Walker-Abbey A, Djokam RR, Eno A, et al. Malaria in pregnant Cameroonian women: the effect of age and gravidity on submicroscopic and mixed-species infections and multiple parasite genotypes. *Am J Trop Med Hyg* 2005; **72**(3): 229-35.
17. Mockenhaupt FP, Rong B, Till H, et al. Submicroscopic *Plasmodium falciparum* infections in pregnancy in Ghana. *Trop Med Int Health* 2000; **5**(3): 167-73.
18. Mockenhaupt FP, Ulmen U, von Gaertner C, Bedu-Addo G, Bienzle U. Diagnosis of placental malaria. *J Clin Microbiol* 2002; **40**(1): 306-8.

19. Malhotra I, Dent A, Mungai P, et al. Can prenatal malaria exposure produce an immune tolerant phenotype? A prospective birth cohort study in Kenya. *PLoS Med* 2009; **6**(7): e1000116.
20. Leke RF, Bioga JD, Zhou J, et al. Longitudinal studies of *Plasmodium falciparum* malaria in pregnant women living in a rural Cameroonian village with high perennial transmission. *Am J Trop Med Hyg* 2010; **83**(5): 996-1004.
21. Adegnika AA, Verweij JJ, Agnandji ST, et al. Microscopic and sub-microscopic *Plasmodium falciparum* infection, but not inflammation caused by infection, is associated with low birth weight. *Am J Trop Med Hyg* 2006; **75**(5): 798-803.
22. Menendez C, Bardaji A, Sigauque B, et al. A randomized placebo-controlled trial of intermittent preventive treatment in pregnant women in the context of insecticide treated nets delivered through the antenatal clinic. *PLoS ONE* 2008; **3**(4): e1934.
23. Luntamo M, Rantala AM, Meshnick SR, et al. The effect of monthly sulfadoxine-pyrimethamine, alone or with azithromycin, on PCR-diagnosed malaria at delivery: a randomized controlled trial. *PLoS ONE* 2012; **7**(7): e41123.
24. Landis SH, Lokomba V, Ananth CV, et al. Impact of maternal malaria and under-nutrition on intrauterine growth restriction: a prospective ultrasound study in Democratic Republic of Congo. *Epidemiol Infect* 2009; **137**(2): 294-304.
25. Dobano C, Berthoud T, Manaca MN, et al. High production of pro-inflammatory cytokines by maternal blood mononuclear cells is associated with reduced maternal malaria but increased cord blood infection. *Malar J* 2018; **17**(1): 177.
26. Cottrell G, Moussiliou A, Luty AJ, et al. Submicroscopic *Plasmodium falciparum* infections are associated with maternal anemia, premature births, and low birth weight. *Clin Infect Dis* 2015; **60**(10): 1481-8.
27. Oduwole OA, Ejezie GC, Odey FA, et al. Congenital malaria in calabar, Nigeria: the molecular perspective. *Am J Trop Med Hyg* 2011; **84**(3): 386-9.
28. Cohee LM, Kalilani-Phiri L, Boudova S, et al. Submicroscopic malaria infection during pregnancy and the impact of intermittent preventive treatment. *Malar J* 2014; **13**(1): 274.
29. Natureeba P, Ades V, Luwedde F, et al. Lopinavir/Ritonavir-based antiretroviral treatment (ART) versus Efavirenz-based ART for the prevention of malaria among HIV-infected pregnant women. *J Infect Dis* 2014; **210**(12): 1938-45.
30. Patel JC, Mwapasa V, Kalilani L, et al. Absence of association between sickle trait hemoglobin and placental malaria outcomes. *Am J Trop Med Hyg* 2016; **94**(5): 1002-7.
31. Kattenberg JH, Tahita CM, Versteeg IA, et al. Evaluation of antigen detection tests, microscopy, and polymerase chain reaction for diagnosis of malaria in peripheral blood in asymptomatic pregnant women in Nanoro, Burkina Faso. *Am J Trop Med Hyg* 2012; **87**(2): 251-6.
32. Gonzalez R, Mombo-Ngoma G, Ouedraogo S, et al. Intermittent preventive treatment of malaria in pregnancy with mefloquine in HIV-negative women: A multicentre randomized controlled trial. *PLoS Med* 2014; **11**(9): e1001733.
33. Gonzalez R, Desai M, Macete E, et al. Intermittent preventive treatment of malaria in pregnancy with mefloquine in HIV-infected women receiving cotrimoxazole prophylaxis: A multicenter randomized placebo-controlled trial. *PLoS Med* 2014; **11**(9): e1001735.
34. Denoeud-Ndam L, Zannou DM, Fourcade C, et al. Cotrimoxazole prophylaxis versus mefloquine intermittent preventive treatment to prevent malaria in HIV-infected pregnant women: two randomized controlled trials. *J Acquir Immune Defic Syndr* 2013; **65**(2): 198-206.
35. Williams J, Njie F, Cairns M, et al. Non-falciparum malaria infections in pregnant women in West Africa. *Malar J* 2016; **15**(1): 53.
36. Mahamar A, Andemel N, Swihart B, et al. Malaria infection is common and associated with perinatal mortality and preterm delivery despite widespread use of chemoprevention in Mali: An observational study 2010 to 2014. *Clin Infect Dis* 2021; **73**(8): 1355-61.
37. Arinaitwe E, Ades V, Walakira A, et al. Intermittent preventive therapy with sulfadoxine-pyrimethamine for malaria in pregnancy: a cross-sectional study from Tororo, Uganda. *PLoS ONE* 2013; **8**(9): e73073.

38. Daud II, Opinya FO, Midem D, et al. Improved pregnancy outcomes in a prospective study of pregnant women enrolling in an antenatal clinic in Western Kenya. *Health* 2015; **6**: 2651-6.
39. Madanitsa M, Kalilani L, Mwapasa V, et al. Scheduled intermittent screening with rapid diagnostic tests and treatment with dihydroartemisinin-piperaquine versus intermittent preventive therapy with sulfadoxine-pyrimethamine for malaria in pregnancy in Malawi: An open-label randomized controlled trial. *PLoS Med* 2016; **13**(9): e1002124.
40. Nkhoma M, Ashorn P, Ashorn U, et al. Providing lipid-based nutrient supplement during pregnancy does not reduce the risk of maternal *P falciparum* parasitaemia and reproductive tract infections: a randomised controlled trial. *BMC Pregnancy Childbirth* 2017; **17**(1): 35.
41. Mosha D, Chilongola J, Ndeserua R, Mwingira F, Genton B. Effectiveness of intermittent preventive treatment with sulfadoxine-pyrimethamine during pregnancy on placental malaria, maternal anaemia and birthweight in areas with high and low malaria transmission intensity in Tanzania. *Trop Med Int Health* 2014; **19**(9): 1048-56.
42. Francine N, Damien B, Anna F, Michael K, Christevy VJ, Felix KK. Characterization of asymptomatic *Plasmodium falciparum* infection and its risk factors in pregnant women from the Republic of Congo. *Acta Trop* 2016; **153**: 111-5.
43. Desai M, Gutman J, L'lanziva A, et al. Intermittent screening and treatment or intermittent preventive treatment with dihydroartemisinin-piperaquine versus intermittent preventive treatment with sulfadoxine-pyrimethamine for the control of malaria during pregnancy in western Kenya: an open-label, three-group, randomised controlled superiority trial. *Lancet* 2015; **386**(10012): 2507-19.
44. Chaponda EB, Chandramohan D, Michelo C, Mharakurwa S, Chipeta J, Chico RM. High burden of malaria infection in pregnant women in a rural district of Zambia: a cross-sectional study. *Malar J* 2015; **14**: 380.
45. Braun V, Rempis E, Schnack A, et al. Lack of effect of intermittent preventive treatment for malaria in pregnancy and intense drug resistance in western Uganda. *Malar J* 2015; **14**: 372.
46. Arnaldo P, Rovira-Vallbona E, Langa JS, et al. Uptake of intermittent preventive treatment and pregnancy outcomes: health facilities and community surveys in Chokwe district, southern Mozambique. *Malar J* 2018; **17**(1): 109.
47. Kakuru A, Jagannathan P, Muhindo MK, et al. Dihydroartemisinin-piperaquine for the prevention of malaria in pregnancy. *N Engl J Med* 2016; **374**(10): 928-39.
48. Mbouamboua Y, Koukouikila-Koussounda F, Ntoumi F, et al. Sub-microscopic *Plasmodium falciparum* infections in matched peripheral, placental and umbilical cord blood samples from asymptomatic Congolese women at delivery. *Acta Trop* 2019; **193**: 142-7.
49. Accrombessi M, Fievet N, Yovo E, et al. Prevalence and associated risk factors of malaria in the first trimester of pregnancy: A preconceptional cohort study in Benin. *J Infect Dis* 2018; **217**(8): 1309-17.
50. Briggs J, Ategeka J, Kajubi R, et al. Impact of microscopic and submicroscopic parasitemia during pregnancy on placental malaria in a high-transmission setting in Uganda. *J Infect Dis* 2019; **220**(3): 457-66.
51. Singer LM, Newman RD, Diarra A, et al. Evaluation of a malaria rapid diagnostic test for assessing the burden of malaria during pregnancy. *Am J Trop Med Hyg* 2004; **70**(5): 481-5.
52. Perrault SD, Hajek J, Zhong K, et al. Human immunodeficiency virus co-infection increases placental parasite density and transplacental malaria transmission in Western Kenya. *Am J Trop Med Hyg* 2009; **80**(1): 119-25.
53. Adam I, Elbasit IE, Salih I, Elbashir MI. Submicroscopic *Plasmodium falciparum* infections during pregnancy, in an area of Sudan with a low intensity of malaria transmission. *Ann Trop Med Parasitol* 2005; **99**(4): 339-44.
54. Bouyou-Akotet MK, Nzenze-Afene S, Ngoungou EB, et al. Burden of malaria during pregnancy at the time of IPTp/SP implementation in Gabon. *Am J Trop Med Hyg* 2010; **82**(2): 202-9.

55. Kapito-Tembo AP. Malaria and anemia in HIV-infected pregnant women in Malawi: associations with cotrimoxazole prophylaxis, submicroscopic malaria, iron supplementation and iron deficiency: The University of North Carolina at Chapel Hill; 2010.
56. Newman PM, Wanzira H, Tumwine G, et al. Placental malaria among HIV-infected and uninfected women receiving anti-folates in a high transmission area of Uganda. *Malar J* 2009; **8**(1): 254.
57. Nwaefuna EK, Afoakwah R, Orish VN, Egyir-Yawson A, Boampong JN. Effectiveness of intermittent preventive treatment in pregnancy with sulphadoxine-pyrimethamine against submicroscopic falciparum malaria in Central Region, Ghana. *J Parasitol Res* 2015; **2015**: 959427.
58. Elbashir HM, Salih MM, Elhassan EM, Mohmmed AM, Elbashir MI, Adam I. Polymerase chain reaction and histology in diagnosis of placental malaria in an area of unstable malaria transmission in Central Sudan. *Diagn Pathol* 2011; **6**(1): 128.
59. Kyabayinze DJ, Zongo I, Cunningham J, et al. HRP2 and pLDH-based rapid diagnostic tests, expert microscopy, and PCR for detection of malaria infection during pregnancy and at delivery in areas of varied transmission: A prospective cohort study in Burkina Faso and Uganda. *PLoS ONE* 2016; **11**(7): e0156954.
60. Kashif AH, Adam GK, Mohmmed AA, Elzaki SE, Abdelhalim AM, Adam I. Reliability of rapid diagnostic test for diagnosing peripheral and placental malaria in an area of unstable malaria transmission in eastern Sudan. *Diagn Pathol* 2013; **8**(1): 59.
61. Matangila JR, Lufuluabo J, Ibalanky AL, Inocencio da Luz RA, Lutumba P, van Geertruyden JP. Asymptomatic *Plasmodium falciparum* infection is associated with anaemia in pregnancy and can be more cost-effectively detected by rapid diagnostic test than by microscopy in Kinshasa, Democratic Republic of the Congo. *Malar J* 2014; **13**: 132.
62. Lamptey H, Ofori MF, Kusi KA, et al. The prevalence of submicroscopic *Plasmodium falciparum* gametocyte carriage and multiplicity of infection in children, pregnant women and adults in a low malaria transmission area in Southern Ghana. *Malar J* 2018; **17**(1): 331.
63. Fadlelseed OE, Osman ME, Shamseldin NM, Elhussein AB, Adam I. *Plasmodium falciparum* genotypes in matched peripheral, placental and umbilical cord blood in an area characterised by unstable malaria transmission in eastern Sudan. *Heliyon* 2017; **3**(6): e00326.
64. Ruh E, Bateko JP, Imir T, Taylan-Ozkan A. Investigation of pregnancy-associated malaria by microscopy, rapid diagnostic test and PCR in Bandundu, the Democratic Republic of Congo. *Trans R Soc Trop Med Hyg* 2018; **112**(1): 8-13.
65. Natureeba P, Kakuru A, Muhindo M, et al. Intermittent preventive treatment with dihydroartemisinin-piperaquine for the prevention of malaria among HIV-infected pregnant women. *J Infect Dis* 2017; **216**(1): 29-35.
66. Quakyi I, Tornyigah B, Houze P, et al. High uptake of intermittent preventive treatment of malaria in pregnancy is associated with improved birth weight among pregnant women in Ghana. *Sci Rep* 2019; **9**(1): 19034.
67. Samuels AM, Towett O, Seda B, et al. Diagnostic performance of ultra-sensitive rapid diagnostic tests for malaria in pregnant women attending antenatal clinics in western Kenya. 68th annual meeting of the American Society of Tropical Medicine and Hygiene, November 20-24, National Harbor, Maryland, USA; 2019: Am J Trop Med Hyg; 2019. p. 276.
68. Tadesse G, Kamaliddin C, Doolan C, et al. Active case detection of malaria in pregnancy using loop-mediated amplification (LAMP): a pilot outcomes study in South West Ethiopia. *Malar J* 2020; **19**(1): 305.
69. Chauvin P, Menard S, Iriart X, et al. Prevalence of *Plasmodium falciparum* parasites resistant to sulfadoxine/pyrimethamine in pregnant women in Yaounde, Cameroon: emergence of highly resistant pfdhfr/pfdhps alleles. *J Antimicrob Chemother* 2015; **70**(9): 2566–71.
70. Enosse S, Magnussen P, Abacassamo F, et al. Rapid increase of *Plasmodium falciparum* dhfr/dhps resistant haplotypes, after the adoption of sulphadoxine-pyrimethamine as first line treatment in 2002, in southern Mozambique. *Malar J* 2008; **7**: 115.
71. Gutman J, Mwandama D, Wiegand RE, et al. In vivo efficacy of sulphadoxine-pyrimethamine for the treatment of asymptomatic parasitaemia in pregnant women in Machinga District, Malawi. *Malar J* 2015; **14**: 197.

72. Mita T, Venkatesan M, Ohashi J, et al. Limited geographical origin and global spread of sulfadoxine-resistant dhps alleles in *Plasmodium falciparum* populations. *J Infect Dis* 2011; **204**(12): 1980-8.
73. Moussiliou A, De Tove YS, Doritchamou J, et al. High rates of parasite recrudescence following intermittent preventive treatment with sulphadoxine-pyrimethamine during pregnancy in Benin. *Malar J* 2013; **12**: 195.
74. Esu E, Tacoli C, Gai P, et al. Prevalence of the Pfdhfr and Pfdhps mutations among asymptomatic pregnant women in Southeast Nigeria. *Parasitol Res* 2018; **117**(3): 801-7.
75. Artimovich E, Schneider K, Taylor TE, et al. Persistence of sulfadoxine-pyrimethamine resistance despite reduction of drug pressure in Malawi. *J Infect Dis* 2015; **212**(5): 694-701.
76. Tumwebaze P, Tukwasibwe S, Taylor A, et al. Changing antimalarial drug resistance patterns identified by surveillance at three sites in Uganda. *J Infect Dis* 2017; **215**(4): 631-5.
77. Tahita MC, Tinto H, Erhart A, et al. Prevalence of the dhfr and dhps mutations among pregnant women in rural Burkina Faso five years after the introduction of intermittent preventive treatment with sulfadoxine-pyrimethamine. *PLoS One* 2015; **10**(9): e0137440.
78. Guerra M, Neres R, Salgueiro P, et al. *Plasmodium falciparum* genetic diversity in continental Equatorial Guinea before and after introduction of artemisinin-based combination therapy. *Antimicrob Agents Chemother* 2017; **61**(1).
79. Gupta H, Macete E, Bulo H, et al. Drug-resistant polymorphisms and copy numbers in *Plasmodium falciparum*, Mozambique, 2015. *Emerg Infect Dis* 2018; **24**(1): 40-8.
80. Lucchi NW, Okoth SA, Komino F, et al. Increasing prevalence of a novel triple-mutant dihydropteroate synthase genotype in *Plasmodium falciparum* in western Kenya. *Antimicrob Agents Chemother* 2015; **59**(7): 3995-4002.
81. Ndiaye D, Dieye B, Ndiaye YD, et al. Polymorphism in dhfr/dhps genes, parasite density and ex vivo response to pyrimethamine in *Plasmodium falciparum* malaria parasites in Thies, Senegal. *Int J Parasitol Drugs Drug Resist* 2013; **3**: 135-42.
82. Desai M, Gutman J, Taylor SM, et al. Impact of sulfadoxine-pyrimethamine resistance on effectiveness of intermittent preventive therapy for malaria in pregnancy at clearing infections and preventing low birth weight. *Clin Infect Dis* 2016; **62**(3): 323–33.
83. Diawara F, Steinhardt LC, Mahamar A, et al. Measuring the impact of seasonal malaria chemoprevention as part of routine malaria control in Kita, Mali. *Malar J* 2017; **16**(1): 325.
84. Baraka V, Ishengoma DS, Fransis F, et al. High-level *Plasmodium falciparum* sulfadoxine-pyrimethamine resistance with the concomitant occurrence of septuple haplotype in Tanzania. *Malar J* 2015; **14**: 439.
85. Nkoli Mandoko P, Rouvier F, Matendo Kakina L, et al. Prevalence of *Plasmodium falciparum* parasites resistant to sulfadoxine/pyrimethamine in the Democratic Republic of the Congo: emergence of highly resistant pfdhfr/pfdhps alleles. *J Antimicrob Chemother* 2018; **73**(10): 2704-15.
86. Baraka V, Delgado-Ratto C, Nag S, et al. Different origin and dispersal of sulfadoxine-resistant *Plasmodium falciparum* haplotypes between Eastern Africa and Democratic Republic of Congo. *Int J Antimicrob Agents* 2017; **49**(4): 456-64.
87. Conrad MD, Mota D, Foster M, et al. Impact of intermittent preventive treatment during pregnancy on *Plasmodium falciparum* drug resistance-mediating polymorphisms in Uganda. *J Infect Dis* 2017; **216**(8): 1008-17.
88. Huijben S, Macete E, Mombo-Ngoma G, et al. Counter-selection of antimalarial resistance polymorphisms by intermittent preventive treatment in pregnancy. *J Infect Dis* 2020; **221**(2): 293-303.
89. Chico RM, Chaponda EB, Ariti C, Chandramohan D. Sulfadoxine-Pyrimethamine exhibits dose-response protection against adverse birth outcomes related to sexually transmitted and reproductive tract infections. *Clin Infect Dis* 2017; **64**(8): 1043-51.

90. Unger HW, Aho C, Ome-Kaius M, et al. Impact of intermittent preventive treatment in pregnancy with azithromycin-containing regimens on maternal nasopharyngeal carriage and antibiotic sensitivity of Streptococcus pneumoniae, Haemophilus influenzae, and Staphylococcus aureus: a cross-sectional survey at delivery. *J Clin Microbiol* 2015; **53**(4): 1317-23.
